# Anticancer activity and toxicity of new quaternary ammonium geldanamycin derivative salts and their mixtures with potentiators

**DOI:** 10.1080/14756366.2021.1960829

**Published:** 2021-08-04

**Authors:** Natalia Skrzypczak, Krystian Pyta, Piotr Ruszkowski, Przemysław Mikołajczak, Małgorzata Kucińska, Marek Murias, Maria Gdaniec, Franz Bartl, Piotr Przybylski

**Affiliations:** a Faculty of Chemistry, Adam Mickiewicz University, Poznan, Poland; b Department of Pharmacology, Poznan University of Medical Sciences, Poznan, Poland; c Department of Toxicology, Poznan University of Medical Sciences, Poznań, Poland; d Lebenswissenschaftliche Fakultät, Institutfür Biologie, Biophysikalische Chemie Humboldt-Universität zu Berlin Invalidenstrasse 42, Berlin, Germany

**Keywords:** Ansamycins, benzoquinones, chaperone Hsp90, anticancer, SAR

## Abstract

Geldanamycin (**GDM**) has been modified by different type neutral/acidic/basic substituents (**1**–**7**) and by quinuclidine motif (**8**), transformed into ammonium salts (**9**–**13**) at C(17). These compounds have been characterised by spectroscopic and x-ray methods. Derivative **8** shows better potency than **GDM** in MCF-7, MDA-MB-231, A549 and HeLa (IC_50_s = 0.09–1.06 µM). Transformation of **8** into salts **9**–**13** reduces toxicity (by 11-fold) at attractive potency, e.g. MCF-7 cell line (IC_50_∼2 µM). Our studies show that higher water solubility contributes to lower toxicity of salts than **GDM** in healthy CCD39Lu and HDF cells. The use of **13** mixtures with potentiators PEI and DOX enhanced anticancer effects from IC_50_∼2 µM to IC_50_∼0.5 µM in SKBR-3, SKOV-3, and PC-3 cancer cells, relative to **13**. Docking studies showed that complexes between quinuclidine-bearing **8**–**13** and Hsp90 are stabilised by extra hydrophobic interactions between the C(17)-arms and K58 or Y61 of Hsp90.

## Introduction

**Figure 1.**
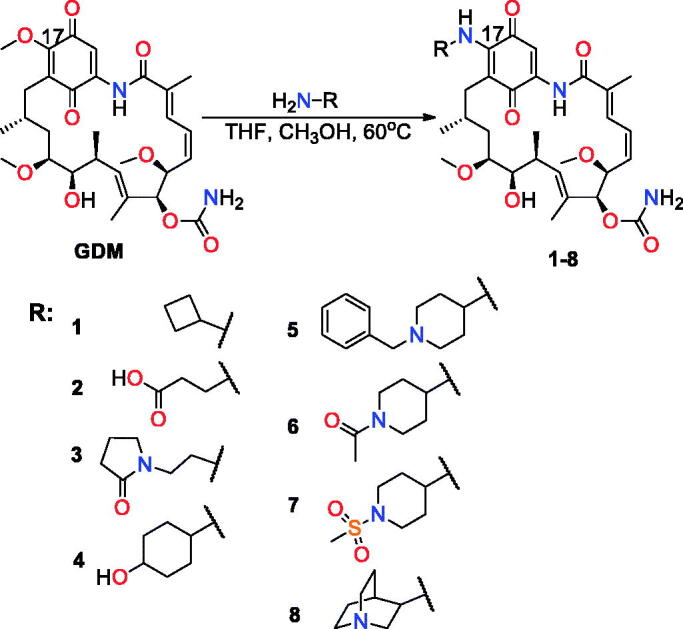
Geldanamycin (**GDM**) and its C(17)- analogues.

Geldanamycin (**GDM**, Figure 1), a natural ansa-macrolide produced by *Streptomyces hygroscopicus*, has two unique structural features, namely a rigid benzoquinone ring and an aliphatic *ansa*-bridge that are linked together to form a characteristic basket-like structure.^1^ Similar to other *ansa*-macrolides with benzene or benzoquinone core, it shows potent activity in different cancer cell lines.^2–4^ Unfortunately, the significant toxicity of **GDM** impedes its future medical applications. This toxicity is believed to originate from 1,4-Michael conjugate addition/aromatisation/oxidation cascade with glutathione, yielding a C(19)-adduct.^5–7^ In search for more active and less toxic benzoquinone and non-benzoquinone ansamycins as well as those with an expanded macrocyclic system, **GDM** was modified at the C(17), C(19) positions and at the methoxy, urethane or hydroxyl groups.^2^^,^^8–10^ The most promising candidates among the C(17)-derivatives of **GDM** are amine derivatives 17-DMAG and 17-AAG that are currently under consideration or are in various phases of clinical trials.^2^^,^^11–13^ Non-quinone analogues of **GDM** (reblastatin analogues, Bioteca, Cambridge, UK) or those containing halogens, saccharides, phenol groups at the core, were obtained by sequences of mutasynthetic, semi-synthetic, and total synthetic approaches. These analogues showed attractive activities against the cancer cell lines, such as human breast adenocarcinoma (SKBR-3, MCF-7, and MDA-MB-231), ovarian adenocarcinoma (SKOV-3), lung adenocarcinoma (A-549), and prostate adenocarcinoma (PC-3).^2^^,^^4^^,^^14–20^ Moreover, modifications at the *ansa*-macrolide correlated to the preparation of conjugates with biotin, foliate, or within corporated triazole bridge, that gained molecular probe features of improved biocompatibility.^21–24^ Overall, the *ansa*-bridge modifications of **GDM** led to a less effective anticancer potency than those performed at the rigid benzene or benzoquinone cores.^25–33^ This can be attributed to a restriction of the *ansa*-bridge flexibility that is necessary for binding the **GDM** analogues to their molecular target, i.e. heat shock proteins Hsp90. Hsp90 shows an ATPase activity, which is required to fold proteins in cells in order to achieve their full functionality.^2^^,^^34–36^ Crystal structures of **GDM** and **17-DMAG** complexed with NBD of Hsp90 showed that the ligand conformation significantly differs from that of unbound molecule in solutions.^27^^,^^28^ Interestingly, modifications of **GDM** at C(17) and C(19) positions sometimes resulted in antiviral potency against herpes (HSV), hepatitis B and C (HCV, HBV) or HIV-1.^37–39^ Additionally, for **GDM** amine analogues where the C(17) substituent was bridged with the neighbouring quinone group at C(18), despite their good water solubility (e.g. guanidine-like derivatives), it was noticed that the bulkiness of the incorporated alkyl or aryl substituent was important for anticancer potency.^40^ Ge et al. obtained reduced (hydroquinone) and highly water-soluble **GDM** derivatives with allylamine or *N*,*N*-dimethylethylamine in the form of salts that are considered in cancer therapy in phase I clinical trials.^41^

**Figure 2.**
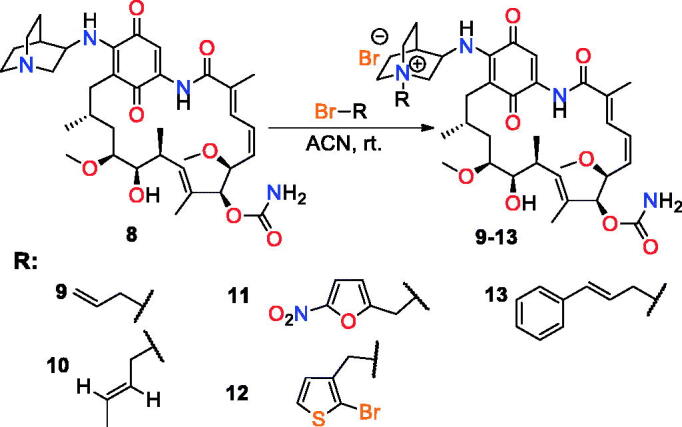
Quaternisation of the quinuclidine *N* atom within **8** leading to analogues **9–13**.

Recently, we have reported synthesis and anticancer activity of a series of C(17)-**GDM** derivatives and we found that rigidity of the C(17)-arm incorporating carbonyl group and lipophilicity are essential factors influencing an efficient binding of **GDM** analogues to Hsp90 and their anticancer potency.^7^ Here, we report the anticancer activity studies of the **GDM** amine-quinuclidine analogue **8 **with C(17)-arm of basic, rigid, and bulky nature. The data have been determined for nine cancerous (MDA-MB-231, MCF-7, HeLa, HepG2, SKBR-3, SKOV-3, PC-3, U-87, and A-549) and two healthy (human lung fibroblasts CCD39Lu, human dermal fibroblasts HDF) cell lines. The activities of **8** are compared with the **GDM** derivatives **1**–**7** of acidic, basic, and neutral nature (Figure 1)^7^^,^^23^^,^^40^. Taking into account the fact that improved water solubility may lead to better bioavailability, derivative **8** was transformed into quaternary *N*-alkylammonium salts **9**–**13** of different *N*-tail structure (Figure 2). Moreover, **GDM** quinuclidine salts were also studied in mixtures with potentiators towards their anticancer potency and in order to obtain information about their toxicity.

## Results and discussion

### Synthesis of GDM analogues

**GDM** derivatives **1**–**8** were obtained according to the earlier reported protocol^7^ and their structures were confirmed by ^1^H, ^13 ^C NMR, and 2D NMR, FT-IR, and ESI MS methods (Supplementary Material). Derivatives **1**–**3** formed single crystals that allowed to determine their structures by X-ray analysis (Table 1S, Figures 53S–59S, Supplemental Material). Comparison of the ^1^H and ^13 ^C NMR spectra of **8** and its *N*-alkylammonium salts (**9**–**13**) with those of **1**–**7** showed only minor differences in the position of the proton and the carbon atom resonances assigned to the quinone and the *ansa*-bridge parts (Figure 60S; Supplemental Material). The NOESY contacts for **GDM** analogues indicated *trans*-configuration of the lactam group with the lactam proton directed into the macrocyclic cavity, as shown in Figure 60S. As found for **8** and **9**–**13** salts, the absolute value of the H(29)-C(29)-N(17)-H(17) torsion angle is in the range of 120–130° (^3^*J*_N(17)H-H(29)_∼6.5–8 Hz,) and the *N*-alkyl substituent of nitrogen *N* + (30) is oriented away from the quinone core (Figure 60S). The nitrogen atom N(17), linking the **GDM** and quinuclidine parts, seems to be present in a distorted *sp*^2^ hybridisation, as suggested by DFT calculations (Figure 60S). The above data indicated similar conformations of **GDM** derivatives in its “free form” in solution and solid, with only small changes in orientation of the C(17)-amine part relative to the quinone core.

### Anticancer potency and toxicity in normal cells of GDM analogues

Quinuclidine derivative **8** and its quaternary *N*-alkylammonium salts (**9**–**13**) were initially tested in MDA-MB-231, MCF-7, HeLa, and HepG2 and healthy CCD39LU cell lines, and compared with biological test results of typical **GDM** C(17)-derivatives (**1**–**7**) (Table 1). Although **GDM** belongs to the known and active anticancer agents, its potency against breast cancer cells (MCF-7) is the lowest among the studied analogues (Table 1, IC_50_ = 3.51 µM). A comparison of **GDM** activity and its analogues in the MCF-7 indicates that derivatives **1, 3,** and **8** are markedly more potent than **GDM**, which is reflected by their low IC_50_∼0.1 µM values. Furthermore, **1, 3,** and **8** show higher SI indexes (∼77, 75, and 10) than **GDM** for the MCF-7 cell line. Derivatives **5**–**7** were also more potent than **GDM** in the MCF-7 cell line (IC_50_∼1 µM), with still favourable SIs∼10. Lipophilicity of the most promising analogues (**1**, **3**, and **5**–**8**) is relatively good (*c*log*P* = 0.7–3.4) and comparable with that of **GDM** (*c*log*P*∼2). Compound **2**, bearing a terminal acidic group, showed the lowest activity in the MCF-7 cell line at a less favourable SI compared to **GDM**. Taking into account the binding models of **GDM** and its derivatives with Hsp90, one should expect that the carboxylic group might improve the binding strength as a result of the interaction with a positively charged K58^7^^,^^27^^,^^28^. However, it is likely that in solution a flexible C(17) arm of **2** takes part in competitive intramolecular interactions (see Supplemental Material, Figure 51S), preventing the expected stabilising interaction with K58 of Hsp90. The existence of the H-bonded structures of **2** in solution and its relatively low lipophilicity (*c*log*P*∼0.4, Table 1), might explain the lower anticancer activity of **2** compared to the analogues **1**, **3**, and **8**. In addition to low anticancer potency, compound **2** exhibits also very low toxicity when compared with **GDM** (IC_50 (CCD39Lu)_ = 20.8 µM, Table 1). As for other biological data for **1**–**8**, the quinuclidine analogue **8** shows the most beneficial potency in MDA-MB-231 (IC_50_ = 0.14 µM) among all ansamycins studied. The activity of **8** relative to **GDM** was improved, not only in MDA-MB-231 but also in other studied cancer cell lines (Table 1). When compared with **8**, **GDM** was only more active in HepG2 cells. Lower activity than that of **GDM** was also observed for all other studied derivatives (**1**–**7**) and salts (**9**–**13**) in the HepG2 cancer cell line. The most promising quinuclidine analogue **8** exhibited relatively high toxicity in healthy cells CCD39Lu (IC_50_ = 0.87 µM) and had only low SI indexes (SIs < 1, Table 1).

**Table 1.** The IC_50_ values [(µM) ± SD], selectivity indexes [SI_CCD39Lu_] as well as *c*log*P* and solubility $S_{H_{2}O}$ parameters for **GDM** and its analogues **1**–**8** and salts (**9**–**13**) obtained in MDA-MB-231, MCF-7, HeLa, HepG2, and CCD39Lu (1st panel) cell lines.

Cmpd.	MDA-MB-231	MCF-7	HeLa	HepG2	CCD39Lu	*c*log*P*	Solubility $S_{H_{2}O}$[mg/mL]
**GDM**	1.04 ± 0.63 [SI 8.17]	3.51 ± 0.21 [SI 2.42]	1.42 ± 0.10 [SI 5.99]	0.74 ± 0.32 [SI 11.48]	8.50 ± 0.25	0.43/ 2.15*	–
**1**	2.45 ± 0.78 [SI > 4.08]	0.13 ± 0.01 [SI > 76.92]	1.67 ± 0.54 [SI > 5.99]	1.75 ± 0.04 [SI > 5.71]	>10	2.24	–
**2**	4.27 ± 1.54 [SI 4.87]	7.62 ± 2.35 [SI 2.73]	3.48 ± 0.51 [SI 5.98]	3.04 ± 0.47 [SI 6.84]	20.80 ± 5.14	0.43	1.47
**3**	1.84 ± 0.33 [SI 4.08]	0.10 ± 0.21 [SI 75.00]	1.25 ± 0.22 [SI 6.00]	1.31 ± 0.54 [SI 5.73]	7.50 ± 0.55	0.67	0.05
**4**	>10 [SI > 1]	>10 [SI > 1]	>10 [SI > 1]	>10 [SI > 1]	>10	1.87	0.04
**5**	2.28 ± 0.54 [SI 6.71]	1.84 ± 0.57 [SI 8.32]	1.56 ± 0.14 [SI 9.81]	1.63 ± 0.49 [SI 9.39]	15.30 ± 0.12	3.40	–
**6**	3.16 ± 0.89 [SI > 3.16]	0.61 ± 0.14 [SI > 16.39]	4.75 ± 0.35 [SI > 2.11]	3.38 ± 0.13 [SI > 2.96]	>10	1.36	–
**7**	2.36 ± 0.35 [SI 5.34]	0.96 ± 0.51 [SI 13.13]	1.61 ± 0.61 [SI 7.83]	1.68 ± 0.32 [SI 7.50]	12.60 ± 0.21	1.38	–
**8**	0.14 ± 0.01 [SI 6.21]	0.09 ± 0.01 [SI 9.67]	1.06 ± 0.26 [SI 0.82]	1.25 ± 0.22 [SI 0.70]	0.87 ± 0.22	2.23	0.15
**9**	4.65 ± 2.65 [SI > 2.15]	2.34 ± 0.56 [SI > 4.27]	9.25 ± 0.98 [SI > 1.08]	>10 [SI > 1]	>10	−1.10	11.9
**10**	4.64 ± 1.51 [SI > 2.16]	2.51 ± 1.03 [SI > 3.98]	7.43 ± 3.21 [SI > 1.35]	>10 [SI > 1]	>10	−0.85	8.09
**11**	3.93 ± 1.60 [SI > 2.54]	4.67 ± 2.00 [SI > 2.14]	8.91 ± 3.19 [SI > 1.12]	8.86 ± 0.17 [SI > 1.13]	>10	−0.81	2.30
**12**	5.01 ± 2.15 [SI > 2.00]	2.65 ± 1.09 [SI > 3.77]	9.12 ± 1.81 [SI > 1.10]	>10 [SI > 1]	>10	0.37	4.50
**13**	4.66 ± 2.49 [SI > 2.15]	2.31 ± 0.97 [SI > 4.33]	8.14 ± 0.64 [SI > 1.23]	>10 [SI > 1]	>10	0.61	5.03

*c*log*P* calculated by *Molinspiration*^42^.

Selectivity index [SI_HDF_] = IC_50_ normal cell line CCD39Lu/IC_50_ respective cancer cell line.

**GDM**: geldanamycin.

Considering the above results, we decided to further test compound **8** using the 2nd panel of cancer cell lines: SKBR-3, SKOV-3, PC-3, U-87, and A-549 and healthy cells HDF and to compare biological data with those of exemplary derivative **1** (Table 2). Our studies demonstrated that **8** is very active in the 2nd panel of cancer cell lines and its activity is even slightly better than that of **GDM** in the A-549 cell line (IC_50_ = 0.99 µM). The potency of **8** in SKBR-3, SKOV-3, PC-3, and U-87 cancer cell lines was lower or comparable to **GDM** (Table 2). In contrast with the results obtained in normal CCD39LU cell line, the toxicity of **8** in healthy cells HDF, is significantly lower than that for **GDM** (Table 2). The potency of low-cytotoxic **1** (IC_50(HDF)_ = 30 µM; IC_50(CCD39Lu)_ = > 10 µM), especially in the 2nd panel of SKBR-3, SKOV-3, PC-3, U-87, and A-549 cancer cell lines, was relatively low (IC_50_∼7.5 µM). Taking into regard the fact that another analogue of low cytotoxicity, **2 **(IC_50(CCD39Lu)_ = 20.8 µM), shows the best water solubility among **1–8** derivatives (Table 1), we decided to transform the most active quinuclidine analogue **8** into its better water-soluble *N*-alkylammonium salts to investigate the influence of a polar structure of this type on anticancer activity *vs*. toxicity. Biological tests of the salts **9**–**13** in the 1st panel of cancer cell lines (Table 1) revealed that similar to **8**, they were generally more active than **GDM** in the MCF-7 cells (except **11**, Table 1). A comparison of IC_50_ values between **8** and **9**–**13**, indicated higher activity of the former. Interestingly, the transformation of **8** into its salts **9**–**13** was beneficial regarding low toxicities of **9**–**12** in normal cell line CCD39Lu (Table 1). Among quaternary salts, analogue **13** with a bulky *N*-cinnamyl quinuclidine moiety showed the best activity in MCF-7 with IC_50_ = 2.31 µM and with twice the value of SI index (SI = 4.33) when compared to **GDM**. Very good results in MCF-7 cells were also obtained for salts **9 **(IC_50_ = 2.34 µM) and **10** (IC_50_ = 2.51 µM), which, like **13**, had no heteroatoms in the tail attached to the quinuclidine unit (Table 1). Other biological data for the 1st panel of cell lines showed that quaternisation of quinuclidine nitrogen in **8** significantly decreased potency in MDA-MB-231, HeLa and HepG2 cancer cell lines while toxicity was reduced in normal CCD39Lu cells (Table 1). Anticancer studies of salts **9**–**13** in the 2nd panel of cell lines (Table 2) revealed that the most active analogue is compound **10 **with no heteroatoms in the *N*-quinuclidine tail. Its IC_50_ values were oscillating around 1.7 µM (1.29 − 1.94 µM). The highest potency of **10 **was registered in the A-549 cancer cell line (IC_50_ = 1.29 µM). Although a comparison of anticancer activities of **GDM**, **8** and **10** (Table 2) showed the lowest potency for **10**, its activity remains attractive in the 2nd panel of cancer cells, at lower cytotoxicity than that of **GDM** in HDF and CCD39Lu cell lines. Salts **9** and **13**, with substituents of similar nature as **10**, also showed good anticancer activities with IC_50_∼2–3 µM and with a markedly reduced toxicity in HDF (IC_50_∼5.29 and 3.81 µM, Table 2). To conclude, considering the biological results for **9**–**13** obtained in the 1st and the 2nd panel of cancer cell lines, when compared to the activity data of **GDM** and **8**, the most promising among the reported salts is salt **13** as it shows the best balance between the anticancer potency and toxicity in normal cells. It is relatively low toxic in both normal cell lines HDF (IC_50_ = 3.81 µM) and CCD39LU (IC_50_ > 10 µM) at still attractive level of anticancer potency IC_50_∼2 µM in MCF-7, SKBR-3, SKOV-3, PC-3, U-87, and A-549 cell lines.

**Table 2.** Anticancer activities [IC_50_ (µM) ± SD] of **GDM** and its **1** and **8** analogues and salts (**9**–**13**) in SKBR-3, SKOV-3, PC-3, U-87, A-549 cells (2nd panel), and toxicity in human dermal fibroblasts (HDF) cell line [IC_50_ (µM) ± SD] together with selectivity indexes [SI_HDF_].

Cmpd.	SKBR-3	SKOV-3	PC-3	U-87	A-549	HDF
**GDM**	0.87 ± 0.17 [SI 2.45]	0.94 ± 0.09 [SI 2.27]	0.73 ± 0.01 [SI 2.92]	0.81 ± 0.12 [SI 2.62]	0.99 ± 0.01 [SI 2.15]	2.13 ± 0.11
**1**	7.44 ± 0.09 [SI 4.03]	8.04 ± 0.24 [SI 3.73]	7.02 ± 0.06 [SI 4.28]	7.32 ± 0.49 [SI 4.10]	9.01 ± 0.63 [SI 3.33]	30.02 ± 1.03
**8**	1.49 ± 0.29 [SI 2.16]	1.16 ± 0.92 [SI 2.78]	1.47 ± 0.31 [SI 2.19]	1.08 ± 0.16 [SI 3.43]	0.94 ± 0.03 [SI 3.43]	3.22 ± 0.18
**9**	3.12 ± 0.01 [SI 1.70]	3.86 ± 0.05 [SI 1.37]	3.01 ± 0.11 [SI 1.76]	3.69 ± 0.08 [SI 1.43]	3.09 ± 0.01 [SI 1.71]	5.29 ± 0.17
**10**	1.94 ± 0.52 [SI 1.53]	1.61 ± 0.13 [SI 1.84]	1.88 ± 0.02 [SI 1.57]	1.84 ± 0.11 [SI 1.61]	1.29 ± 0.07 [SI 2.29]	2.96 ± 0.36
**11**	6.49 ± 0.07 [SI 1.22]	6.04 ± 0.38 [SI 1.31]	6.22 ± 0.13 [SI 1.27]	6.06 ± 0.58 [SI 1.31]	6.83 ± 0.32 [SI 1.16]	7.91 ± 0.91
**12**	8.49 ± 0.36 [SI 1.64]	8.02 ± 0.17 [SI 1.73]	7.94 ± 0.03 [SI 1.75]	8.58 ± 0.19 [SI 1.62]	8.94 ± 0.27 [SI 1.56]	13.91 ± 0.42
**13**	2.02 ± 0.11 [SI 1.89]	2.17 ± 0.06 [SI 1.76]	2.05 ± 0.09 [SI 1.86]	2.26 ± 0.15 [SI 1.67]	2.06 ± 0.01 [SI 1.85]	3.81 ± 0.11

Selectivity index [SI_HDF_] = IC_50_ normal cell line HDF/IC_50_ respective cancer cell line.

### Binding mode of quinuclidine analogues of GDM and 9–13 salts with Hsp90

**Figure 3.**
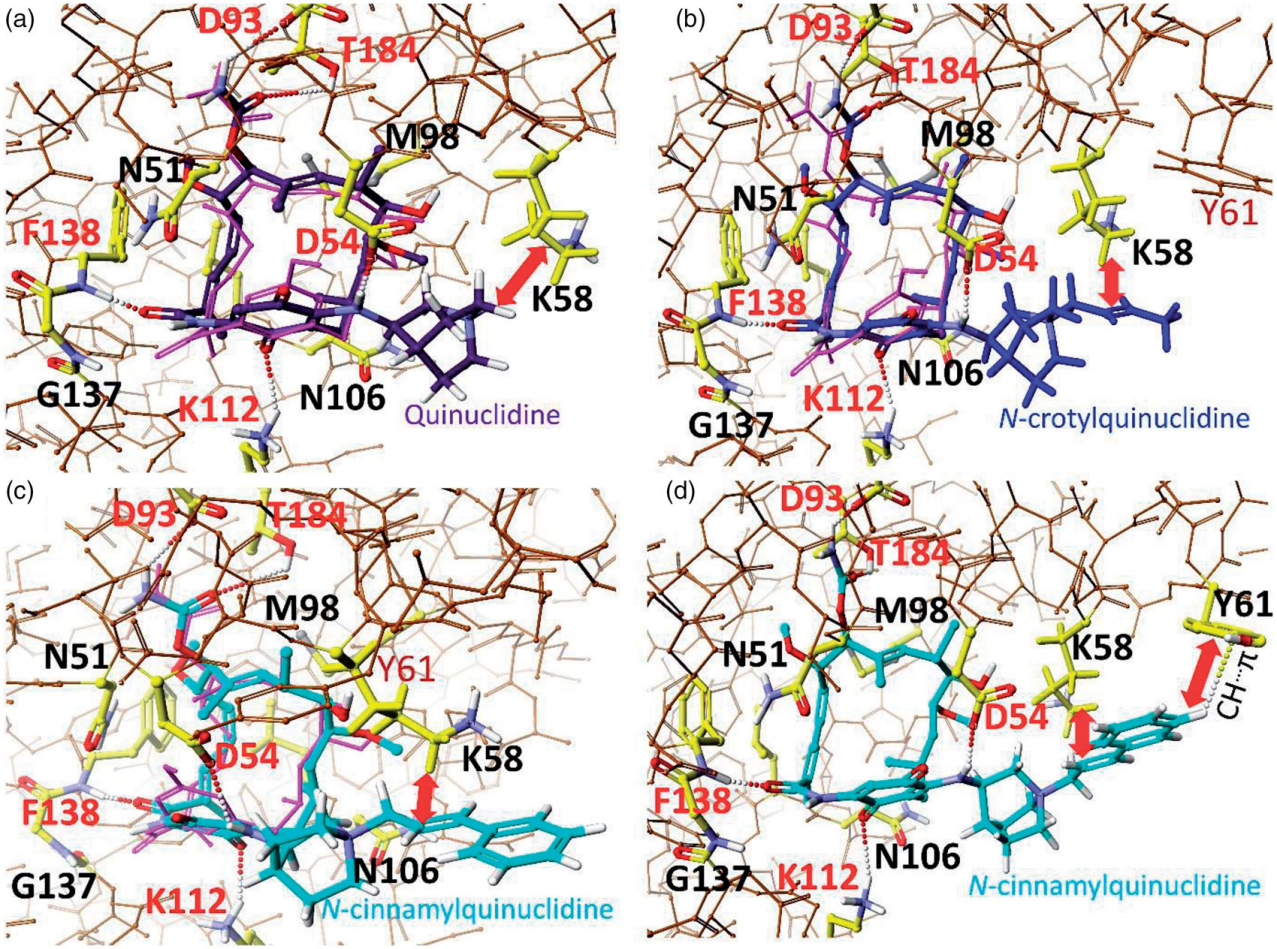
Binding models revealing interactions between *N*-binding domain (NBD) of Hsp90 (PDB 3Q5J[43]) and new ansamyc in derivatives: **8/**ΔH°_f_ = −47 kcal/mol/(a), **10/**ΔH°_f_ = −39 kcal/mol/(b) and **13** in binding mode I **/**ΔH°_f_ = −32 kcal/mol/(c), and **13** in binding mode II **/**ΔH°_f_ = −37 kcal/mol/(d), compared to binding mode of macbecin II (PDB2VW5^35^, pink), and optimised by MOG-PM6 method (*Scigress* F.J. 2.6, EU 3.1.9);^46^ amino acids of Hsp90 ATP-binding pocket are marked by yellow; intermolecular interactions (H-bonds) are marked by dots.

According to the earlier reports,^7^^,^^26–28^^,^^32^^,^^43^ we assumed that docking of **8**, **10**, and **13** into the target, i.e. ATP-binding pocket of Hsp90, requires *trans*/*cis* isomerisation of the lactam group and drastic conformational changes within the *ansa*-bridge owing to atropisomerisation process, i.e. flipping of the *ansa*-bridge from one side of the benzoquinone ring to the other (Figure 62S, Supplemental Material). DFT calculated energy barrier is 36.97 kcal/mol for the total atropisomerisation process of **10** requiring *trans*-*cis* lactam isomerisation crucial for binding with NBD of Hsp90 (Figures 3(b) and Figure 62S; Supplemental Material). This value is higher than that estimated from earlier studies of **GDM** (E = 16–21 kcal/mol).^44^^,^^45^ Structural comparison between complexes of Hsp90 with **GDM** and **8**, showed that the mutual arrangements of the *ansa*-bridges and quinone cores relative to the Hsp90 key amino acids are similar in both cases.^7^^,^^27^ Atropisomerisation of active **GDM** analogues from the *trans*-lactam form (dominating in solution) into the *cis*-lactam one (bound with Hsp90) is evoked by the mutual induced fitting of human Hsp90 and the **GDM** derivative. The **8**-Hsp90 complex is stabilised by the formation of intermolecular H-bonds between the ansamycin and F138, D54, D93, K112, and T184 of Hsp90, whereas, e.g. M98 and K58 are involved in hydrophobic interactions of the ligand in the ATP-binding pocket (Figure 3). Thus, the above binding mode of **8** is slightly improved relative to that known for **GDM** by an extra hydrophobic stabilisation of quinuclidine basket of **8** with K58, contributing also to improved anticancer potency of **8** relative to **GDM**. Comparison between calculated binding models of the most active derivative **8** and salts **10** and **13** of decreased cytotoxicity shows for the latter ones also possibility of hydrophobic stabilisation with K58 of Hsp90 (Figure 3). Comparison of binding energies of **10** (ΔH°_f_**_(10)_** = − 39 kcal/mol) or **8** (ΔH°_f(_**_8_**_)_ = − 47 kcal/mol) with Hsp90 indicates the privileged formation of **8**-Hsp90 complex. This result is in line with higher anticancer potency of higher lipophilic **8** (*c*log*P* = 2.23; $S_{H_{2}O}$= 0.15 mg/mL, Table 1) when compared to better water-soluble **10** (*c*log*P* = −0.85; $S_{H_{2}O}$= 8.09 mg/mL, Table 1). Compound **13**, owing to the presence of its lengthy and bulky quinuclidine arm at C(17), has two alternative binding modes to Hsp90 (Figure 3(c,d)). The binding mode I of **13**, is analogous to that of **8** and **10** (the interaction with K58, Figure 3(c)). The binding mode II of **13** to Hsp90 is realised *via* a conformational change within the quinuclidine basket, where the *N*^+^-cinnamyl tail is involved in an intermolecular C-H^…^π interaction with Y61 of Hsp90 (Figure 3(d)). A more favourable binding mode II to Hsp90 is excluded for **8** and **10 **due to shorter quinuclidine arms at C(17). Overall, the higher anticancer activity of **8** compared to **10** and **13** can be explained by a higher energetic profit of binding **8** to Hsp90 (ΔH°_f _= − 47 kcal/mol) than for **10** (ΔH°_f _= − 39 kcal/mol) and **13** (ΔH°_f _= − 37 kcal/mol). Changes in lipophilicity (*c*log*P*) do not explain the differences in the anticancer effect of the tested salts (**9**–**13**). In turn, the better water solubility of salts **9**, **10,** and **13** ($S_{H_{2}O}$ > 5 mg/mL) seems to be in line with their better anticancer effects than **11** ($S_{H_{2}O}$ = 2.30 mg/mL) and **12** ($S_{H_{2}O}$ = 4.5 mg/mL), especially in the 2nd panel of tested cancer cell lines.

### Anticancer tests of the most active quinuclidine analogues with potentiators

**Figure 4.**
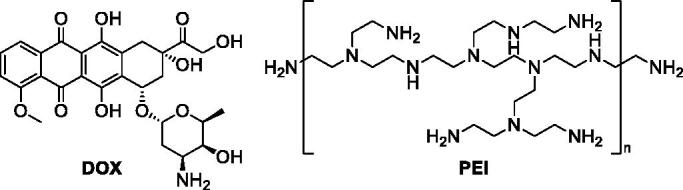
Structures of tested selected potentiators of quinuclidine analogues of **GDM**.

It is known that the effectiveness and potency of various antibiotics can be improved when they are used in cocktails with adjuvants and potentiators allowing, e.g. effective transportation of a drug into the target site of action.^47–49^ In order to evaluate the influence of the addition of doxorubicin (**DOX;** Figure 4) or branched polyethylenediamine – (**PEI,** polyethylenimine; Figure 4) on the activity of our lead compounds **8** and **13**, tests were performed in SKBR-3, PC-3, SKOV-3, and HDF cells for their 1:1 equimolar mixtures (Table 3).

**Table 3.** Anticancer activities [IC_50_ (µM) ± SD] of **8** and **13** and their equimolar 1:1 mixtures with **DOX** and **PEI** in SKBR-3, SKOV-3, and PC-3, compared with toxicity determined in normal human dermal fibroblasts (HDF) cell line [IC_50_ (µM) ± SD] and selectivity indexes [SI_HDF_].

Cmpd.	SKBR-3	SKOV-3	PC-3	HDF
**8**	1.49 ± 0.29 [SI 2.16]	1.16 ± 0.92 [SI 2.78]	1.47 ± 0.31 [SI 2.19]	3.22 ± 0.18
**8 + DOX**	1.06 ± 0.08 [SI 2.42]	1.74 ± 0.02 [SI 1.48]	1.71 ± 0.11 [SI 1.50]	2.57 ± 0.07
**8 + PEI**	2.61 ± 0.11 [SI 1.53]	2.04 ± 0.01 [SI 1.96]	2.68 ± 0.03 [SI 1.49]	3.99 ± 0.22
**13**	2.02 ± 0.11 [SI 1.89]	2.17 ± 0.06 [SI 1.76]	2.05 ± 0.09 [SI 1.86]	3.81 ± 0.11
**13 + DOX**	1.33 ± 0.21 [SI 2.40]	1.49 ± 0.16 [SI 2.14]	1.09 ± 0.01 [SI 2.93]	3.19 ± 0.31
**13 + PEI**	0.55 ± 0.02 [SI 1.93]	0.62 ± 0.05 [SI 1.71]	0.59 ± 0.01 [SI 1.80]	1.06 ± 0.06
**DOX**	0.72 ± 0.04 [SI 2.11]	0.74 ± 0.06 [SI 2.05]	0.68 ± 0.01 [SI 2.24]	1.52 ± 0.03
**PEI**	0.53 ± 0.05 [SI 1.55]	0.69 ± 0.01 [SI 1.19]	0.51 ± 0.02 [SI 1.61]	0.82 ± 0.11

Selectivity index [SI_HDF_] = IC_50_ normal cell line HDF/IC_50_ respective cancer cell line.

As shown in Table 3, **DOX** reveals a comparable activity at IC_50_∼0.7 µM towards all studied cancer cell lines at enhanced toxicity in the HDF normal cell line, relative to those of **GDM** (Table 2). Compounds **8** and **13** are of lower potency in studied cancer cell lines, although they simultaneously exhibit reduced toxic effects in HDF cell line, when referred to **DOX** and **PEI**. With the addition of **PEI** to **8** and **13** opposite changes in the potency were noted. The equimolar mixture **13 + PEI** showed significantly improved potency relative to that of **13** (IC_50(_**_13+PEI_**_)_∼0.6 µM *vs.* IC_50(_**_13_**_)_∼2 µM). Interestingly **13 + PEI** mixture showed even slightly higher anticancer potency (IC_50_ = 0.62 µM) than **PEI** itself in SKOV-3 cells. Unfortunately, with the increased potency of mixture **13 + PEI**, its toxicity also showed a 3-fold increase in HDF cells in comparison with **13**. In turn, the combined use of **8** with **PEI** resulted in decreased anticancer activity and cytotoxicity, both relative to **8** and **PEI**. The use of **8**+**DOX** and **13**+**DOX** mixtures improved potency in SKBR-3 cells relative to **8** and **13**, respectively. For the **13**+**DOX** mixture, the analogous result was also obtained in SKOV-3 and PC-3, in contrast to **8**+**DOX**. The markedly enhanced anticancer activities of **13**+**DOX** in studied cancer cell lines relative to that of **13**, is accompanied by nearly preserved toxicity in HDF cells (IC_50 _level∼3 µM). Thus, our studies showed that the use of better water-soluble salt **13** than compound **8**, in an equimolar mixture with **PEI** and **DOX**, contribute to markedly improved anticancer activity in SKBR-3, SKOV-3, and PC-3 (IC_50_∼3 µM), however, at the expense of increased toxicity in normal cells, when referred to **13**. Furthermore, tests with **13**+**DOX** mixture showed some available compromise between relatively preserved cytotoxicity (at the level IC_50_∼3 µM) and enhanced anticancer potency to IC_50_∼1 µM, especially in the PC-3 cell line (SI∼3).

## Conclusions

The quinuclidine analogue **8** of **GDM** and its *N*-alkylammonium salts **9**–**13** have been synthesised and tested in nine cancer (MDA-MB-231, MCF-7, HeLa, HepG2, SKBR-3, SKOV-3, PC-3, U-87, and A-549) and two normal (CCD39Lu and HDF) cell lines. The biological activities of these compounds were compared with those of **GDM** and C(17)-analogues **1**–**7**. Structural studies of **1**–**13** using 1D and 2D NMR and x-ray crystallography methods revealed nearly identical conformation of the *ansa*-bridge, the *trans* configuration of the lactam group and similar arrangement of the C(17) substituents relative to the quinone core in solution and in solid. The MTT assay revealed that **8** is the most active derivative among studied C(17)-analogues of **GDM** in the 1st panel of MDA-MB-231, MCF-7, HeLa and HepG2 cancer cell lines at low IC_50s_∼0.1–1 µM. The anticancer activities of **8** in these cancer cell lines were markedly higher than **GDM**, except for the HepG2 cell line. A higher potency of **8** than **GDM** was noted also in the A-549 cell line of the 2nd panel. Unfortunately, alongside the attractive potency of **8**, higher and lower toxicities than **GDM** were noted in CCD39Lu and HDF normal cell lines, respectively. Studies in MCF-7 cancer cell line revealed improved activities of *N*-alkylammonium salts **9**–**13** when compared to **GDM** (e.g. IC_50(_**_13_**_)_ = 2.31 µM *vs.* IC_50(_**_GDM_**_)_ = 3.51 µM), at together reduced toxic effects in HDF and CCD39Lu normal cell lines (IC_50_ even > 10 µM). Our studies showed that the limited toxicity of **9**–**13** salts can be linked with their low *c*log*P* and improved water solubility relative to **GDM** (almost water-insoluble). In studied cancer cell lines, among **9**–**13** salts the most potent were those without heteroatoms in the attached tail at the nitrogen of C(17)-quinuclidine basket. Molecular docking of the most potent salts **8**, **10**, and **13** indicated the intermolecular hydrophobic stabilisation of the quaternary C(17)-quinuclidine arm with K58 or with Y61 of Hsp90. A more beneficial binding energy of the **8**-Hsp90 complex than that for **10** or **13** complexes explains a higher anticancer activity of **8 **than **10** and **13**. Our studies also showed that quaternisation of the nitrogen within C(17)-quinuclidine containing arm can be a useful strategy in decreasing the toxicity of **GDM** analogues in normal cells, at a simultaneously improved or preserved anticancer activity in MCF-7 and A-549 cancer cell lines, respectively (e.g. compound **10**). The use of **13** with potentiator **PEI** leads to improved or comparable anticancer activities relative to those of the salt and **PEI**, respectively. However, this beneficial anticancer effect was observed at the expense of increased toxicity in normal cells, when referred to **13**.

## Supplementary Material

Supplemental Material

## References

[1] Baksh A, Kepplinger B, Isah HA, et al. Production of 17-O-demethyl-geldanamycin, a cytotoxic ansamycin polyketide, by streptomyces hygroscopicus DEM20745. Nat Prod Res 2017;31:1895–900. 27966376 10.1080/14786419.2016.1263854

[2] Franke J, Eichner S, Zeilinger C, Kirschning A. Targeting heat-shock-protein 90 (Hsp90) by natural products: geldanamycin, a show case in cancer therapy. Nat Prod Rep 2013;30:1299–323. 23934201 10.1039/c3np70012g

[3] Fukuyo Y, Hunt CR, Horikoshi N. Geldanamycin and its anti-cancer activities. Cancer Lett 2010;290:24–35. 19850405 10.1016/j.canlet.2009.07.010

[4] Hermane J, Eichner S, Mancuso L, et al. New geldanamycin derivatives with anti Hsp properties by mutasynthesis. Org Biomol Chem 2019;17:5269–78. 31089638 10.1039/c9ob00892f

[5] Guo W, Reigan P, Siegel D, Ross D. Enzymatic reduction and glutathione conjugation of benzoquinone ansamycin heat shock protein 90 inhibitors: relevance for toxicity and mechanism of action. Drug Metab Dispos 2008;36:2050–7. 18635747 10.1124/dmd.108.022004 PMC2574845

[6] Cysyk RL, Parker RJ, Barchi JJ, et al. Reaction of geldanamycin and C17-substituted analogues with glutathione: product identifications and pharmacological implications. Chem Res Toxicol 2006;19:376–81. 16544941 10.1021/tx050237e

[7] Skrzypczak N, Pyta K, Ruszkowski P, et al. Synthesis, structure and anticancer activity of new geldanamycin amine analogs containing C(17)- or C(20)- flexible and rigid arms as well as closed or open ansa-bridges. Eur J Med Chem 2020;202:112624. 32663707 10.1016/j.ejmech.2020.112624

[8] Kitson RRA, Moody CJ. An improved route to 19-substituted geldanamycins as novel Hsp90 inhibitors-potential therapeutics in cancer and neurodegeneration. Chem Commun (Camb) 2013;49:8441–3. 23770604 10.1039/c3cc43457e PMC3835074

[9] Li Z, Jia L, Wang J, et al. Discovery of diamine-linked 17-aroylamido-17-demethoxygeldanamycins as potent Hsp90 inhibitors. Eur J Med Chem 2014;87:346–63. 25277067 10.1016/j.ejmech.2014.09.078

[10] Eichner S, Eichner T, Floss HG, et al. Broad substrate specificity of the amide synthase in *S. hygroscopicus-new 20-membered macrolactones derived from geldanamycin*. J Am Chem Soc 2012;134:1673–9. 22136518 10.1021/ja2087147 PMC3292439

[11] Mellatyar H, Talaei S, Pilehvar-Soltanahmadi Y, et al. Targeted cancer therapy through 17-DMAG as an Hsp90 inhibitor: overview and current State of the art. Biomed Pharmacother 2018;102:608–17. 29602128 10.1016/j.biopha.2018.03.102

[12] Talaei S, Mellatyar H, Asadi A, et al. Spotlight on 17-AAG as an Hsp90 inhibitor for molecular targeted cancer treatment. Chem Biol Drug Design 2019;93:760–86. 10.1111/cbdd.13486 30697932

[13] Glaze ER, Lambert AL, Smith AC, et al. Preclinical toxicity of a geldanamycin analog, 17-(dimethylaminoethylamino)-17-demethoxygeldanamycin (17-DMAG), in rats and dogs: potential clinical relevance. Cancer Chemother Pharmacol 2005;56:637–47. 15986212 10.1007/s00280-005-1000-9

[14] Wu CZ, Jang JH, Woo M, et al. Enzymatic glycosylation of nonbenzoquinone geldanamycin analogs *via* bacillus UDP-glycosyltransferase. Appl Environ Microbiol 2012;78:7680–6. 22923401 10.1128/AEM.02004-12 PMC3485733

[15] Hermane J, Bułyszko I, Eichner S, et al. New, non-Quinone fluorogeldanamycin derivatives strongly inhibit Hsp90. ChemBioChem 2015;16:302–11. 25572106 10.1002/cbic.201402375

[16] Mohammadi-Ostad-Kalayeh S, Stahl F, Scheper T, et al. Heat shock proteins revisited: using a mutasynthetically generated reblastatin library to compare the inhibition of human and *Leishmania* Hsp90s. ChemBioChem 2018;19:562–74. 29265716 10.1002/cbic.201700616

[17] Kirschning A, Hahn F. Merging chemical synthesis and biosynthesis: a new chapter in the total synthesis of natural products and natural product libraries. Angew Chem Int Ed Engl 2012;51:4012–22. 22441812 10.1002/anie.201107386

[18] Bułyszko I, Dräger G, Klenge A, Kirschning A. Evaluation of the synthetic potential of an AHBA knockout mutant of the rifamycin producer *Amycolatopsis Mediterranei*. Chemistry 2015;21:19231–42. 26559164 10.1002/chem.201503548

[19] Eichner S, Knobloch T, Floss HG, et al. The interplay between mutasynthesis and semisynthesis: generation and evaluation of an ansamitocin library. Angew Chem Int Ed Engl 2012;51:752–7. 22135226 10.1002/anie.201106249

[20] Mancuso L, Jürjens G, Hermane J, et al. Bioreduction of aryl azides during mutasynthesis of new ansamitocins. Org Lett 2013;15:4442–5. 23981134 10.1021/ol401989e

[21] Harmrolfs K, Mancuso L, Drung B, et al. Preparation of New Alkyne-Modified Ansamitocins by Mutasynthesis. Beilstein J Org Chem 2014;10:535–43. 24605171 10.3762/bjoc.10.49 PMC3943755

[22] Schax E, Walter JG, Märzhäuser H, et al. Microarray-based screening of heat shock protein inhibitors. J Biotechnol 2014;180:1–9. 24667540 10.1016/j.jbiotec.2014.03.006

[23] Tian ZQ, Liu Y, Zhang D, et al. Synthesis and biological activities of novel 17-aminogeldanamycin derivatives. Bioorg Med Chem 2004;12:5317–29. 15388159 10.1016/j.bmc.2004.07.053

[24] Greish K, Ray A, Bauer H, et al. Anticancer and antiangiogenic activity of HPMA copolymer-aminohexylgeldanamycin-RGDfK conjugates for prostate cancer therapy. J Control Release 2011;151:263–70. 21223983 10.1016/j.jconrel.2010.12.015 PMC3095727

[25] Li L, Wang L, You QD, Xu XL. Heat shock protein 90 inhibitors: an update on achievements, challenges, and future directions. J Med Chem 2020;63:1798–822. 31663736 10.1021/acs.jmedchem.9b00940

[26] Raman S, Singh M, Tatu U, Suguna K. First structural view of a peptide interacting with the nucleotide binding domain of heat shock protein 90. Sci Rep 2015;5:17015–0. 26599366 10.1038/srep17015 PMC4657054

[27] Stebbins CE, Russo AA, Schneider C, et al. Crystal structure of an Hsp90-geldanamycin complex: targeting of a protein chaperone by an antitumor agent. Cell 1997;89:239–50. 9108479 10.1016/s0092-8674(00)80203-2

[28] Jez JM, Chen JCH, Rastelli G, et al. Crystal structure and molecular modeling of 17-DMAG in complex with human Hsp90. Chem Biol 2003;10:361–8. 12725864 10.1016/s1074-5521(03)00075-9

[29] Rastelli G, Tian ZQ, Wang Z, et al. Structure-based design of 7-carbamate analogs of geldanamycin. Bioorg Med Chem Lett 2005;15:5016–21. 16165354 10.1016/j.bmcl.2005.08.013

[30] Immormino RM, Metzger LE, Reardon PN, et al. Different poses for ligand and chaperone in inhibitor-bound Hsp90 and GRP94: implications for paralog-specific drug design. J Mol Biol 2009;388:1033–42. 19361515 10.1016/j.jmb.2009.03.071 PMC2692672

[31] Sawarkar R, Roy N, Rao S, et al. Heat shock protein 90 regulates development in dictyostelium discoideum. J Mol Biol 2008;383:24–35. 18718841 10.1016/j.jmb.2008.08.006

[32] Zhang MQ, Gaisser S, Nur-E-Alam M, et al. Optimizing natural products by biosynthetic engineering: discovery of nonquinone Hsp90 inhibitors ^†^. J Med Chem 2008;51:5494–7. 18800759 10.1021/jm8006068

[33] Wernimont AK, Tempel W, Lin YH, et al. Crystal structure of the amino-terminal domain of HSP90 from leishmania major, LMJF33.0312:M1-K213 in the presence of 17-DMAP-geldanamycin. Available from: 10.2210/pdb3q5j/pdb

[34] Bhat R, Tummalapalli SR, Rotella DP. Progress in the discovery and development of heat shock protein 90 (Hsp90) inhibitors. J Med Chem 2014;57:8718–28. 25141341 10.1021/jm500823a

[35] Massey AJ. ATPases as drug targets: insights from heat shock proteins 70 and 90. J Med Chem 2010;53:7280–6. 20608738 10.1021/jm100342z

[36] Kitson RRA, Moody CJ. Learning from nature: advances in geldanamycin- and radicicol-based inhibitors of Hsp90. J Org Chem 2013;78:5117–41. 23496136 10.1021/jo4002849

[37] Shan G, Peng Z, Li Y, et al. A novel class of geldanamycin derivatives as HCV replication inhibitors targeting on Hsp90: synthesis, structure-activity relationships and anti-HCV activity in GS4.3 replicon cells. J Antibiot (Tokyo) 2011;64:177–82. 21179047 10.1038/ja.2010.161

[38] Li YP, Shan GZ, Peng ZG, et al. Synthesis and biological evaluation of heat-shock protein 90 inhibitors: geldanamycin derivatives with broad antiviral activities. Antivir Chem Chemother 2010;20:259–68. 20710066 10.3851/IMP1631

[39] Anderson I, Low JS, Weston S, et al. Heat shock protein 90 controls HIV-1 reactivation from latency. Proc Natl Acad Sci USA 2014;111:E1528–E1537. 24706778 10.1073/pnas.1320178111 PMC3992654

[40] Schnur RC, Corman ML, Gallaschun RJ, et al. Inhibition of the oncogene product P185erbB-2 in vitro and in vivo by geldanamycin and dihydrogeldanamycin derivatives. J Med Chem 1995;38:3806–12. 7562911 10.1021/jm00019a010

[41] Ge J, Normant E, Porter JR, et al. Design, synthesis, and biological evaluation of hydroquinone derivatives of 17-amino-17-demethoxygeldanamycin as potent, water-soluble inhibitors of Hsp90. J Med Chem 2006;49:4606–15. 16854066 10.1021/jm0603116

[42] Molinspiration V2018.10. Available from: https://www.Molinspiration.Com/

[43] Bank, RPD. RCSB PDB - 3Q5J: crystal structure of the amino-terminal domain of HSP90 from Leishmania major, LMJF33.0312: M1-K213 in the presence of 17-DMAP-geldanamycin Available from: https://www.rcsb.org/structure/3Q5J [accessed 7 May 2020].

[44] Thepchatri P, Eliseo T, Cicero DO, et al. Relationship among ligand conformations in solution, in the solid state, and at the Hsp90 binding site: geldanamycin and radicicol. J Am Chem Soc 2007;129:3127–34. 17323946 10.1021/ja064863p

[45] Lee YS, Marcu MG, Neckers L. Quantum chemical calculations and mutational analysis suggest heat shock protein 90 catalyzes trans-cis isomerization of Geldanamycin. Chem Biol 2004;11:991–8. 15271357 10.1016/j.chembiol.2004.05.010

[46] Scigress package FJ 2.6/EU 3.1.9./2008–2019; Japan: Fujitsu.

[47] Douafer H, Andrieu V, Phanstiel O, Brunel JM. Antibiotic adjuvants: make antibiotics great again!. J Med Chem 2019;62:8665–81. 31063379 10.1021/acs.jmedchem.8b01781

[48] Liu TY, Hussein WM, Giddam AK, et al. Polyacrylate-based delivery system for self-adjuvanting anticancer peptide vaccine. J Med Chem 2015;58:888–96. 25489968 10.1021/jm501514h

[49] Domalaon R, Idowu T, Zhanel GG, Schweizer F. Antibiotic hybrids: the next generation of agents and adjuvants against gram-negative pathogens? Clin Microbiol Rev 2018;31:e00077–17. 29540434 10.1128/CMR.00077-17 PMC5967690

