# Supplementary material for: Anticancer activity and toxicity of new quaternary ammonium geldanamycin derivative salts and their mixtures with potentiators
Source: J Enzyme Inhib Med Chem. 2021 Aug 4;36(1):1898–904. doi: 10.1080/14756366.2021.1960829 (PMC8344233; doi:10.1080/14756366.2021.1960829)
Supplement: Supplemental Material [file IENZ_A_1960829_SM9512.pdf]

Supplementary Material

Anticancer activity and toxicity of new quaternary ammonium geldanamycin derivative salts and their mixtures with potentiators

Natalia Skrzypczak,[1] Krystian Pyta,[1] Piotr Ruszkowski,[2] Przemysław Mikołajczak,[2] Małgorzata Kucińska,[3] Marek Murias,[3] Maria Gdaniec,[1] Franz Bartl,[4] Piotr Przybylski[1]*

[1]*Faculty of Chemistry, Adam Mickiewicz University, Uniwersytetu Poznanskiego 8, 61-614, Poznań, Poland*

[2]*Department of Pharmacology, Poznan University of Medical Sciences, Rokietnicka 5a, 60-806 Poznań, Poland*

[3]*Department of Toxicology, Poznan University of Medical Sciences, Poznań, Poland*

[4]*Lebenswissenschaftliche Fakultät, Institut für Biologie, Biophysikalische Chemie Humboldt-Universität zu Berlin Invalidenstrasse 42 10099 Berlin, Germany*

*corresponding author e-mail: piotrp@amu.edu.pl

Figure 1S. Cytotoxic activity of tested compounds against (A) MDA-MB-231, (B) MCF-7, (C) HeLa, (D) HepG2 and (E) CCD39Lu cell lines. 21
Figure 2S. Cytotoxic activity of tested compounds against (A) MDA-MB-231, (B) MCF-7, (C) HeLa, (D) HepG2 and (E) CCD39Lu cell lines. 21
Figure 3S. ^{1}H NMR spectrum of compound **1** in $CDCl_3$. 22
Figure 4S. ^{13}C NMR spectrum of compound **1** in $CDCl_3$. 23
Figure 5S. FT-IR spectrum of compound **1** (in KBr). 24
Figure 6S. ^{1}H NMR spectrum of compound **2** in $CDCl_3$. 25
Figure 7S. ^{13}C NMR spectrum of compound **2** in $CDCl_3$. 26
Figure 8S. FT-IR spectrum of compound **2** (in KBr). 27
Figure 9S. ^{1}H NMR spectrum of compound **3** (in $CDCl_3$). [5] 28
Figure 10S. ^{13}C NMR spectrum of compound **3** (in $CDCl_3$). [5] 29
Figure 11S. FT-IR spectrum of compound **3** (in KBr).[5] 30
Figure 12S. ^{1}H NMR spectrum of compound **4** (in $CDCl_3$). [5] 31
Figure 13S. ^{13}C NMR spectrum of compound **4** (in $CDCl_3$). [5] 32
Figure 14S. FT-IR spectrum of compound **4** (in KBr). [5] 33
Figure 15S. ^{1}H NMR spectrum of compound **5** (in $CDCl_3$). [5] 34
Figure 16S. ^{13}C NMR spectrum of compound **5** (in $CDCl_3$). [5] 35
Figure 17S. FT-IR spectrum of compound **5** (in KBr). [5] 36
Figure 18S. ^{1}H NMR spectrum of compound **6** (in $CDCl_3$). [5] 37
Figure 19S. ^{13}C NMR spectrum of compound **6** (in $CDCl_3$). [5] 38
Figure 20S. FT-IR spectrum of compound **6** (in KBr). [5] 39
Figure 21S. ^{1}H NMR spectrum of compound **7** (in $CDCl_3$). [5] 40
Figure 22S. ^{13}C NMR spectrum of compound **7** (in $CDCl_3$). [5] 41
Figure 23S. FT-IR spectrum of compound **7** (in KBr).[5] 42
Figure 24S. ^{1}H NMR spectrum of compound **8** in $CDCl_3$. 43
Figure 25S. ^{13}C NMR spectrum of compound **8** in $CDCl_3$. 44
Figure 26S. FT-IR spectrum of compound **8** (in KBr). 45
Figure 27S. ^{1}H NMR spectrum of compound **9** in $CDCl_3$. 46
Figure 28S. ^{13}C NMR spectrum of compound **9** in $CDCl_3$. 47
Figure 29S. FT-IR spectrum of compound **9** (in KBr). 48
Figure 30S. ESI-MS^{+} spectrum of **9**. 49
Figure 31S. ^{1}H-^{1}H NOESY contacts recorded for compound **9**. 50
Figure 32S. ^{1}H-^{1}H NOESY contacts recorded for compound **9**. 51
Figure 33S. ^{1}H-^{1}H NOESY contacts recorded for compound **9**. 52
Figure 34S. ^{1}H NMR spectrum of compound **10** in $CDCl_3$. 53
Figure 35S. ^{13}C NMR spectrum of compound **10** in $CDCl_3$. 54
Figure 36S. FT-IR spectrum of compound **10** (in KBr). 55
Figure 37S. ESI-MS^{+} spectrum of **10**. 56
Figure 38S. ^{1}H NMR spectrum of compound **11** in CD_3CN. 57
Figure 39S. ^{13}C NMR spectrum of compound **11** in CD_3CN. 58
Figure 40S. FT-IR spectrum of compound **11** (in KBr). 59
Figure 41S. ESI-MS^{+} spectrum of **11**. 60
Figure 42S. ^{1}H NMR spectrum of compound **12** in $CDCl_3$. 61

Figure 43S. ^{13}C NMR spectrum of compound **12** in $CDCl_3$. 62
Figure 44S. FT-IR spectrum of compound **12** (in KBr). 63
Figure 45S. ESI-MS$^+$ spectrum of **12**. 64
Figure 46S. ^{1}H NMR spectrum of compound **13** in $CDCl_3$. 65
Figure 47S. ^{13}C NMR spectrum of compound **13** in $CDCl_3$. 66
Figure 48S. FT-IR spectrum of compound **13** (in KBr). 67
Figure 49S. ESI-MS$^+$ spectrum of **13**. 68
Figure 50S. Structures of **1,2,8-13** derivatives presented in ChemDraw Ultra 12.0. 69
Figure 51S. Two possibilities of an alternative intramolecular H-bonding within structure of derivative **2** together with xyz coordinates (see below**),** visualized by DFT B88-LYP method. [1] 70
Figure 52S. Structure and xyz coordinates (see below) of derivative **9** (Fig. 2 – manuscript), calculated by DFT B88-LYP method [1] 78
Figure 53S. Energy barrier calculations for atropisomerization of **10**, performed by BLYP GGA (DZP) DFT theoretical method (ADF package) [3,4] 83
Figure 54S. Molecular structure of **1**. Displacement ellipsoids are shown at the 50% probability level. Hydrogen atoms from N-H and O-H groups are shown and those from C-H groups were omitted for clarity. 86
Figure 55S. Molecular structure of **2**. Displacement ellipsoids are shown at the 50% probability level. Hydrogen atoms from N-H and O-H groups are shown and those from C-H groups were omitted for clarity. The carboxylic group of C17 substituent is disordered over two positions. 87
Figure 56S. Molecular structure of **3 in 3_1**. Displacement ellipsoids are shown at the 50% probability level. Hydrogen atoms from N-H and O-H groups are shown and those from C-H groups were omitted for clarity. The C17 substituent of the A molecule is disordered over two positions. 89
Figure 57S. Molecular structure of **3 in 3_2**. Displacement ellipsoids are shown at the 50% probability level. The hydrogen atoms from N-H and O-H groups are shown and those from C-H groups were omitted for clarity. The ethylene fragment of the C17 substituent is disordered over two positions. 89
Figure 58S. Side (a) and top (b) views of a two dimensional supramolecular assembly formed reproducibly via hydrogen bonds in C-17 substituted geldanamycin derivatives. 90
Figure 59S. Comparision of crystal packing of 2D supramolecular hydrogen-bonded assemblies in **1** (a), **2** (b), **3-1** (c) and **3-2** (d) viewed along the corresponding direction in the crystal lattice 91
Figure 60S. General conformation of **GDM** quaternary ammonium salts in solution, determined on the basis of experimentally recorded key ^{1}H-^{1}H NOESY contacts, and calculated on that basis *via* B88 LYP (GGA) DFT method (*Scigress* package [1]); N(1)H contacts (violet), H(15) contacts (red), H(19) contacts (green). 92
Figure 61S. Illustration of the A conformer of C(17) **GDM** analog on an example of one of the symmetry independent molecules in the crystal structure of **2**. The amino N(17)-H group is oriented towards the C(18)=O(18) carbonyl group. 93
Figure 62S. Comparison of salt **10** conformations: bound with Hsp90 (*cis*-lactam, violet) and "free" in solution (*trans*-lactam, orange) calculated by B3LYP GGA (DZP) DFT (ADF package)[3,4], and visualized by Scigress (*Scigress* F.J. 2.6, EU 3.1.9)[1]. 94

Table 1S. Crystal data and details of structure refinement. 85

General Experimental:

Geldanamycin was purchased from Carbosynth, batch number AG236511801. Solvents $CDCl_3$ for NMR spectroscopic measurements as well as, methanol, acetone, cyclobutylamine, β-alanine, (R)-(+)-3-aminoquinuclidine dihydrochloride, allyl bromide, crotyl bromide, 2-(bromomethyl)-5-nitrofuran, 2-bromo-3-(bromomethyl)thiophene, 3-bromo-1-phenyl-1-propene, ACN, TEA and THF used for the syntheses of **GDM** derivatives were purchased from Sigma-Aldrich. While methanol, methylene chloride and acetone used for column chromatography were purchased from Chemsolve. H_2O HPLC gradient grade and CH_3CN HPLC gradient grade were purchased from J. T. Baker.

HPLC measurements:

The purity of the **GDM** analogs **1-8**, found in all cases as ≥ 95%, was determined by HPLC method using Dionex Ultimate 3000 equipped with an LPG-3400 SD gradient pump using Thermo GOLD C18 150×4.6 mm (5 μm) and Accucore XL column, TCC-3000SD thermostat to columns (column temp. equal 25 ºC) and Dionex VWD- 3400RS variable wavelength UV-vis detector (detection at λ_{max}=220 and 260 nm); the flow rates were 0.5 mL/min with injection volumes of 10 μL in acetonitrile mixtures and the mobile phase: 35:65 H_2O/CH_3CN.

FT-IR measurements:

The FT-IR spectra of **GDM** and its analogs **1-13** were recorded in KBr pellet. FT-IR measurements were performed at spectrometer equipped with a DTGS detector and two-columnar purge gas generator at resolution 1 cm^{-1}, NSS = 150, range 4000-400 cm^{-1}. The Happ-Genzel apodization function was used.

NMR measurements:

The ^{1}H and ^{13}C measurements of derivatives of **GDM** (**1-13**) were performed in $CDCl_3$ using Varian Mercury 400 MHz and 500 MHz Bruker BioSpin GmBH spectrometers. The operating frequencies for ^{1}H measurements were 401.15, 500.25 MHz; example parameters for a 500

MHz: spectral width, sw = 11520.7 Hz; acquisition time at = 2.8443 s; relaxation delay d_1 = 1.0 s; T = 298.0 K; TMS was used as the internal standard. No window function or zero filling were used. Digital resolution was 0.2 Hz/point. ^{13}C NMR spectra were recorded at the operating frequency 125.80 MHz; sw = 31250.0 Hz, at = 1.0486 s, d_1 = 1.0 s, T = 298.0 K, and TMS as the internal standard. Line broadening parameters of 0.5 or 1 Hz were applied. ^{1}H and ^{13}C NMR resonances unambiguously assigned on the basis of the ^{1}H–^{13}C HMBC, ^{1}H–^{13}C HSQC, and ^{1}H–^{1}H COSY couplings.

ESI MS analyses:

The mass spectra were recorded on ZQ Waters spectrometer using Electrospray ionization method in positive ion detection mode (range of m/z range from 100 to 2000).

Elemental analyses:

The elemental analyses of new **GDM** analogs **1-13** were carried out on Vario ELIII (Germany).

MO-G PM6 molecular docking, single molecule B88 LYP (GGA) DFT calculations and energetic barrier estimation on the basis of BLYP GGA (DZP) DFT calculations

Model of *N*-quinuclidine–crotyl salt **10** was initially built for single molecule calculations on the basis of our x-ray structures of **1-3** *via* replacing of C(17)-substituent. Next some mutual relative arrangements of the *ansa*-bridge groups and C(17)-substituent were assumed according to recorded ^{1}H-^{1}H NOESY proton-proton contacts. Such prepared structure has been subjected to B88 LYP (GGA) DFT calculations (*Scigress F.J. 2.6, EU 3.1.9* package, Fujitsu[1]). Similar approach has been used for calculations of an alternative structures in solution for compound 2 (Fig. 51S). Docking of **8, 10** and **13** salts were performed using x-ray structure of *N*-binding domain of chaperone Hsp90 (PDB 3Q5J[2]) replacing R97 into K112 amino acid, analogous as in structure of human orthologs of Hsp90. Available x-ray structure was enriched by addition of H-atoms and parametrized by introducing of hybridization of all atoms in the system. Initial models of our **8**, **10** and **13** analogs at Hsp90 pocket have been built using our x-ray structures

of compounds **1**, **2** and **3** (see x-ray studies) of which C(17) substituent was modified and a *cis*-lactam atropisomers were assumed and calculated. Docking of **12, 20** and **25** structures was carried out by "dock into active site" function using coordinates of **17-DMAP** bound to Hsp90 (PDB 3Q5J[2], *Scigress F.J. 2.6, EU 3.1.9* package)[1]. Then, a more distanced amino acids from the site occupied by **GDM** analog at the pocket (>8 Å) were locked at carbon and nitrogen atoms of the main polypeptide chain. The intermolecular interactions (H-bonds, hydrophobic) between molecules of **GDM** analogs and the key amino acid residues (first–shell up to 8 Å) building ATP-binding pocket within NBD of Hsp90 were optimized: N51, D54, K58, D93, M98, L107, N106, K112, F138 and T184 using MO-G PM6 semi-empirical method and MOZYME algorithm dedicated to huge molecules, at energy gradient not exceeding 7 kcal/mol at one step. The calculation procedure at the first stage was based on the holding of Hsp90's atoms locked and the *ansa*-macrolide unlocked and at the second stage the ligand atoms were kept locked at unlocked atoms of Hsp90. In a result of this approach - gradual mutual fitting between **GDM** salts (**8**, **10** and **13**) and NBD of Hsp90 docking poses were obtained as shown in Fig. 3.

Energetic barrier of atropisomerization was calculated for model of salt **13**, which was initially built analogously as to those of the other above-described salts. The two linear transit sequences for **13** were applied using XC functional GGA:BLYP and DZP basis set with the changing dihedral angles within the whole aliphatic *ansa*-bridge in order to obtain transformation from the *trans*-lactam atropisomer (structure in solution) into the *cis*-lactam one (structure bonded to Hsp90) using ADF[3,4]. Crucial points of bond rotation within the *ansa*-bridge were near lactam, quinone and diene moieties. From the obtained graphs E *vs.* type of conformer, where energy was expressed in Hartree units, the value of energy barrier was recalculated into kcal/mol units *via* x*627.509391 kcal/mol.

Biological assays :

Human cancer cells SKBR-3 (human breast cancer cell line) and SKOV-3 (ovarian cancer cell line) were cultured in McCoy's Modified Medium. Human cancer cells PC-3 (human prostate cancer cell line) were cultured in F-12K medium, as well as A549 cell line (lung cancer cell line). U-87MG cells (glioblastoma cell line) were cultured in Eagle's Minimal Essential Medium. Human Dermal Fibroblasts cell line (HDF) was cultured in Fibroblast Basal Medium. Each medium was supplemented with 10% fetal bovine serum, 1% L-glutamine, and 1% penicillin/streptomycin solution. The cell lines were kept in the incubator at 37°C. The optimal plating density of cell lines was determined to be 5×10^4. All the cell lines and mediums were obtained from American Type Culture Collection (ATCC) supplied by LGC-Standards. The protein-staining SRB (Sigma-Aldrich) microculture colorimetric assay, developed by the National Cancer Institute (USA) for in vitro antitumor screening was used in this study, to estimate the cell number by providing a sensitive index of total cellular protein content, being linear to cell density. The monolayer cell culture was trypsinized and the cell count was adjusted to 5×10^4 cells. To each well of the 96 well microtiter plate, 0.1 mL of the diluted cell suspension (approximately 10,000 cells) was added. After 24 hours, when a partial monolayer was formed, the supernatant was washed out and 100 μL of six different compound concentrations (0.1, 0.2, 1, 2, 10, and 20 μM) were added to the cells in microtitre plates. The tested compounds were dissolved in DMSO (containing 10 % of water) (100 μL) and the content of DMSO did not exceed 0.1%; this concentration was found to be nontoxic to the cell lines. The cells were exposed to compounds for 72 hours at 37 °C in a humidified atmosphere (90% RH) containing 5% CO_2. After that, 25 μL of 50 % trichloroacetic acid was added to the wells and the plates were incubated for 1 hour at 4°C. The plates were then washed out with the distilled water to remove traces of medium and next dried by the air. The air-dried plates were stained with 100 μL of 0.4% sulforhodamine B (prepared in 1 % acetic acid) and kept for 30 minutes at room temperature. The unbound dye was removed by rapidly washing with 1%

acetic acid and then air dried overnight. The protein-bound dye was dissolved in 100 μL of 10 mM unbuffered Tris base (pH 10.5) for optical density determination at 490 nm. All cytotoxicity experiments were performed three times. Cell survival was measured as the percentage absorbance compared to the control (nontreated cells). Cytarabine (**ara-C**) and **GDM** were used as the internal standards. Additionally biological assays were performed in Human Dermal Fibroblasts cell line (HDF) in aim to evaluate cytotoxicity of **GDM** and its **1-13** analogs in healthy cells. Results of anticancer studies of novel analogs of **GDM** are shown in Table 2. SI indexes were calculated from equation SI = $IC_{50\ \text{normal cell line HDF}}/IC_{50\ \text{respective cancerous cell line}}$ (Table 2). A beneficial SI > 1.0 indicates a compound with efficacy against tumor cell greater than the toxicity against normal cells.

Cell culture

All experiments were carried out with human breast adenocarcinoma (MDA-MB-231 and MCF-7), hepatocellular carcinoma (HepG2), human cervical adenocarcinoma (HeLa) cancer cell lines, and human normal lung fibroblast (CCD39Lu) cell line. The cell line was purchased from the European Collection of Cells Cultures (ECACC, Salisbury, UK), and cultured in DMEM with phenol red supplemented with 10% (v/v) FBS, 1% (v/v) penicillin/streptomycin, and 1% (v/v) L-glutamine at 37 °C, in a humidified atmosphere containing 5% CO_2.

Tested compounds

The tested compounds were dissolved in DMSO to the final concentration 10 mM and stored in the dark at -20 °C.

Cytotoxicity assay

The cytotoxic effect of the tested compounds was determined by MTT assay. MDA-MB-231, MCF-7, HepG2, HeLa, and CCD39Lu cells were seeded in 96 wells plates at density $2x10^4$ cells/well and incubated overnight under cell culture condition. Next, cells were treated with tested compounds at concentrations of 10 μM; 5 μM; 2.5 μM; 1.2 μM; 0.6 μM; 0.3 μM; 0.15

µM. Moreover, GDM (for MCF-7) and 8 (for MCF-7 and MDA-MB-231) due to high cytotoxicity, were additionally tested at a lower concentration range of 2.5 µM; 1.2 µM; 0.6 µM; 0.3 µM; 0.15µM; 0.07 µM; 0.03 µM. DMSO was used as a control, and the concentration in medium did not exceed 0.1%. Tested compounds were added to cells in the medium without phenol red. Cells were incubated 72 h at 37 °C in a humidified atmosphere containing 5% CO_2. Then, the medium was aspirated and MTT solution at a concentration of 0.59 mg/mL in cell culture medium was added to each well. Plates were incubated for 1.5 hours at 37 °C, and then plates were centrifuged for 3 minutes. The formazan crystals were dissolved in 200 µl DMSO and plates were agitated on a plate shaker at 300–500 rpm for 10 minutes. Absorbance at 570 nm was measured using a plate reader (Biotek Instruments, Elx-800). Data were normalized to the mean absorption of control cells. All experiments were performed at least three times.

X-ray studies of 1-3 crystals:

1-3 analogs (~ 0.1 mg) were placed in an Eppendorf tubes and dissolved in a mixtures of 50 µL of ACN and 50 µL of n-hexane for **1**, 50 µL of ACN and 50 µL of acetone for **2**, 100 µL of acetone for **3**. Solutions were kept at 5 °C for slow evaporation of the solvent. Diffraction experiments for analogs **1-3**, were carried out at low temperature (130 K) with an Oxford Diffraction SuperNova diffractometer using Cu Kα radiation. For more details concerning x-ray structural analyses see section 'x-ray crystallography' of Supplementary Material. CIF files for **1**, **2**, **3-1** and **3-2** were deposited with Cambridge Crystallographic Data Centre (CCDC) and have deposition numbers CCDC 2035723-2035726.

Synthetic procedures and spectral characteristic of GDM analogs:

Synthetic procedures of **GDM** and derivatives **3-7** with the analytical data of them (HRMS, HPLC and elemental data) are included in Ref. [5].

(4E,6Z,8S,9S,10E,12S,13R,14S,16R)-19-(Cyclobutylamino)-13-hydroxy-8,14-dimethoxy-4,10,12,16-tetramethyl-3,20,22-trioxo-2-azabicyclo[16.3.1]docosa-1(21),4,6,10,18-pentaen-9-yl carbamate (compound **1**): 50 mg (0.09 mmol) of **GDM** was dissolved in a 3.3 mL mixture of THF/MeOH (10:1) and then a four-fold excess of cyclobutylamine was added (51 mg, 0.36 mmol). Then, to the each mixture, 0.5 mL of TEA was added. The mixtures were stirred at 60 °C for a 3 hours and after that the solvent was evaporated. Product was purified by column chromatography on silica gel (25 cm × 1 cm, silica gel 60, 0.040−0.063 mm/230−400 mesh ASTM, Fluka) with methylene chloride/acetone (10:1) as an eluent. The product was obtained as a violet powder. Yield 91 %, mp 135-137 ºC, HPLC R_t= 10.746 min. Anal. Calcd for $C_{32}H_{45}N_3O_8$: C, 64.09; H, 7.56; N, 7.01. Found: C, 64.10; H, 7.56; N, 7.02; HRMS (ESI-TOF) m/z: $[M + H]^+$ Calcd 599.3207; Found 599.3204. FT-IR (KBr): $\nu_{as}(N\text{-}H)_{carbamate}$=3484.03 cm^{-1}, $\nu_s(N\text{-}H)_{carbamate}$=3433.07 cm^{-1}, $\nu_s(N\text{-}H)_{lactam}$=3327.32 cm^{-1}, ν(O-H) =3207.82 cm^{-1}, $\nu_s(C\text{-}H)$ =2935.83 cm^{-1}, $\nu(C{=}O)_{carbamate}$ =1734.83 cm^{-1}, $\nu(C{=}O)_{lactam}$=1680.39 cm^{-1}, $\nu(C{=}O)_{quinone}$=1648.10 cm^{-1}, ν(C=C) =1616.10cm^{-1} , $\delta(N\text{-}H)^*_{lactam,substituent}$ =1589.70 cm^{-1} , $\delta(N\text{-}H)_{carbamate}$ =1494.43 cm^{-1}, $\nu(C\text{-}N)_{carbamate}$ =1333.12 cm^{-1}, $\nu(C\text{- }O\text{-}C)_{carbamate}$ =1199.21 cm^{-1}, $\nu(C\text{-}O\text{-}C)_{methoxy}$ =1058.27cm^{-1} , $\gamma(N\text{-}H)_{carbamate}$ =784.19 cm^{-1}. ^{1}H NMR (500 MHz, $CDCl_3$) δ 9.15 (s, 1H, 1-NH), 7.25 (s, 1H, 19-H), 6.94 (d, $J^3_{H\text{-}3,H\text{-}4}$ = 11.7 Hz, 1H, 3-H), 6.57 (td, $J^3_{H\text{-}3,H\text{-}4}$ = 11.4 Hz, $J^4_{H\text{-}4,H\text{-}22}$ = 1.2 Hz, 1H, 4-H), 6.46 (d, $J^3_{NH\text{-}17,H\text{-}29}$ = 7.1 Hz, 1H, 17-NH), 5.92 – 5.85 (m, 1H, 9-H), 5.84 (t, $J^3_{H\text{-}4,H\text{-}5}$ = 10.5 Hz, 1H, 5-H), 5.17 (s, 1H, 7-H), 4.82 (s, 2H, 24-NH_2), 4.35 – 4.31 (m, 1H, 29-H), 4.33 – 4.27 (m, 1H, 6-H), 4.22 (vbs, 1H, 11-OH), 3.59 – 3.52 (m, 1H, 11-H), 3.47 – 3.41 (m, 1H, 12-H), 3.35 (s, 3H, 27-H), 3.26 (s, 3H, 23-H), 2.80 – 2.69 (m, 1H, 10-H), 2.62 (d, J^2 = 13.8 Hz, 1H, 15-H), 2.57 – 2.46 (m, 1H, 30-H), 2.40 – 2.29 (m, 1H, 32-H), 2.24 (dd, J^2 = 13.9 Hz, $J^3_{H\text{-}14,H\text{-}15}$ = 10.5 Hz, 1H, 15-H), 2.09 – 2.04 (m, 1H, 30-H), 2.01 (d, $J^4_{H\text{-}4,H\text{-}22}$ = 1.4 Hz, 3H, 22-H), 1.98 – 1.93 (m, 1H, 32-H), 1.91 – 1.82 (m, 1H, 31-H), 1.85 – 1.78 (m, 1H, 31-H), 1.81 – 1.76 (m, 5H, 13,25-H), 1.76 – 1.69 (m, 1H, 14-H), 0.99 (d, $J^3_{H\text{-}10,H\text{-}}$

$_{26}$ = 6.9 Hz, 3H, 26-H), 0.94 (d, $J^3_{H-14,H-28}$ = 6.7 Hz, 3H, 28-H). ^{13}C NMR (126 MHz, $CDCl_3$) δ 184.2 (18-C), 180.9 (21-C), 168.6 (1-C), 156.3 (24-C), 144.1 (17-C), 141.6 (20-C), 136.1 (5-C), 135.2 (2-C), 134.1 (9-C), 133.0 (8-C), 127.2 (3-C), 126.8 (4-C), 109.0 (16-C), 108.8 (19-C), 82.0 (7-C), 81.8 (12-C), 81.5 (6-C), 72.9 (11-C), 57.4 (23-C), 57.0 (27-C), 49.8 (29-C), 35.3 (13-C), 34.7 (15-C), 33.3 (32-C), 32.6 (10-C), 31.5 (30-C), 28.8 (14-C), 23.2 (28-C), 15.0 (31-C), 13.0 (22-C), 12.9 (25-C), 12.6 (26-C).

3-(((4E,6Z,8S,9S,10E,12S,13R,14S,16R)-9-(Carbamoyloxy)-13-hydroxy-8,14-dimethoxy-4,10,12,16-tetramethyl-3,20,22-trioxo-2-azabicyclo[16.3.1]docosa-1(21),4,6,10,18-pentaen-19-yl)amino)propanoic acid (compound **2**): 50 mg (0.09 mmol) of **GDM** was dissolved in a 3.3 mL mixture of THF/MeOH (10:1) and then a two-fold excess of β-Alanine was added (15.9 mg, 0.18 mmol). Then, to the each mixture, 0.5 mL of TEA was added. The mixtures were stirred at 60 °C for a 3 hours and after that the solvent was evaporated. Product was purified by column chromatography on silica gel (25 cm × 1 cm, silica gel 60, 0.040−0.063 mm/230−400 mesh ASTM, Fluka) with methylene chloride/methanol (10:1) as an eluent. The product was obtained as a violet powder. Yield 85 %, mp 159-161 ºC, HPLC R_t= 3.915 min. Anal. Calcd for $C_{31}H_{43}N_3O_{10}$: C, 60.28; H, 7.02; N, 6.80. Found: C, 60.27; H, 7.01; N, 6.81; HRMS (ESI-TOF) m/z: $[M + H]^+$ Calcd 617.2948; Found 617.2948. FT-IR (KBr): ν_s(N-H)$_{carbamate}$=3463.14 cm^{-1}, ν_s(N-H)$_{lactam}$=3325.07 cm^{-1}, ν(O-H) =3189.65 cm^{-1}, ν_s(C-H) =2925.35 cm^{-1}, ν(C=O)$_{carbamate+substituent}$=1727.55 cm^{-1}, ν(C=O)$_{lactam}$=1690.69 cm^{-1}, ν(C=O)$_{quinone}$=1652.53 cm^{-1}, ν(C=C) =1611.12cm^{-1} , δ(N-H)*$_{lactam,substituent}$ =1574.97 cm^{-1} , δ(N-H)$_{carbamate}$ =1489.02 cm^{-1}, ν(C-N)$_{carbamate}$ =1375.83 cm^{-1}, ν(C- O-C)$_{carbamate}$ =1188.58 cm^{-1}, ν(C-O-C) $_{methoxy}$ =1098.73cm^{-1} , γ(N-H)$_{carbamate}$ =785.25 cm^{-1} . ^{1}H NMR (500 MHz, $CDCl_3$) δ 9.12 (s, 1H, 1-NH), 7.24 (s, 1H, 19-H), 6.92 (d, $J^3_{H-3,H-4}$ = 11.7 Hz, 1H, 3-H), 6.60 (d, $J^3_{NH-17,H-29}$ = 5.9 Hz, 1H, 17-NH), 6.57 (t, $J^3_{H-3,H-4}$ = 11.4 Hz, 1H, 4-H), 5.91 – 5.86 (m, 1H, 9-H), 5.83 (t,

$J^3_{H-4,H-5}$ = 10.5 Hz, 1H, 5-H), 5.20 (s, 1H, 7-H), 5.03 (bs, 2H, 24-NH_2), 4.32 – 4.28 (m, 1H, 6-H), 3.89 – 3.79 (m, 2H, 29-H), 3.58 – 3.54 (m, 1H, 11-H), 3.47 – 3.43 (m, 1H, 12-H), 3.35 (s, 3H, 27-H), 3.26 (s, 3H, 23-H), 2.76 – 2.70 (m, 1H, 10-H), 2.70 – 2.67 (m, 2H, 30-H), 2.66 – 2.63 (m, 1H, 15-H), 2.42 – 2.38 (m, 1H, 11-OH), 2.38 – 2.32 (m, 1H, 15-H), 2.03 (s, 4H, 22-H, 31-OH), 1.78 (s, 5H, 13,25-H), 1.70 (s, 1H, 14-H), 0.99 (d, $J^3_{H-10,H-26}$ = 6.9 Hz, 3H, 26-H), 0.97 (d, $J^3_{H-14,H-28}$ = 6.7 Hz, 3H, 28-H). ^{13}C NMR (126 MHz, $CDCl_3$) δ 184.0 (18-C), 180.9 (21-C), 173.7 (31-C), 168.5 (1-C), 156.8 (24-C), 145.0 (17-C), 141.3 (20-C), 135.8 (5-C), 135.2 (2-C), 133.9 (9-C), 132.8 (8-C), 127.0 (3-C), 126.8 (4-C), 109.1* (16, 19-C), 82.1 (7-C), 81.5 (12-C), 81.3 (6-C), 72.8 (11-C), 57.3 (23-C), 56.9 (27-C), 41.3 (29-C), 35.2 (13-C), 34.5 (15-C), 34.0 (30-C), 32.4 (10-C), 28.7 (14-C), 23.0 (28-C), 12.9 (22-C), 12.8 (25-C), 12.5 (26-C).

(4E,6Z,8S,9S,10E,12S,13R,14S,16R)-13-Hydroxy-8,14-dimethoxy-4,10,12,16-tetramethyl-3,20,22-trioxo-19-((1S,4S)-quinuclidin-3-ylamino)-2-azabicyclo[16.3.1]docosa-1(21),4,6,10,18-pentaen-9-yl carbamate (compound **8**): 400 mg (0.71 mmol) of **GDM** was dissolved in a 16.5 mL mixture of THF/MeOH (10:1) and then a four-fold excess of (R)-(+)-3-Aminoquinuclidine dihydrochloride was added (568.26 mg, 2.84 mmol). Then, to the each mixture, 2.5 mL of TEA was added. The mixtures were stirred at 60 °C for a 5 hours and after that the solvent was evaporated. Product was purified by column chromatography on silica gel (25 cm × 1 cm, silica gel 60, 0.040−0.063 mm/230−400 mesh ASTM, Fluka) with methylene chloride/methanol (20:1) as an eluent. The product was obtained as a violet powder. Yield 94 %, mp 151-154 ºC, HPLC R_t= 4.619 min. Anal. Calcd for $C_{35}H_{50}N_4O_8$: C, 64.20; H, 7.70; N, 8.56. Found: C, 64.21; H, 7.71; N, 8.56; HRMS (ESI-TOF) m/z: $[M + H]^+$ Calcd 654.3629; Found 654.3628. FT-IR (KBr): $\nu_{as}(N\text{-}H)_{carbamate}$=3474.82 cm^{-1}, $\nu_s(N\text{-}H)^*_{carbamate}$, $\nu_s(N\text{-}H)^*_{lactam}$=3310.30cm^{-1}, ν(O-H) =3193.70 cm^{-1}, $\nu_s(C\text{-}H)$ =2931.99 cm^{-1}, $\nu(C{=}O)_{carbamate}$ =1728.34 cm^{-1}, $\nu(C{=}O)_{lactam}$=1691.06 cm^{-1}, $\nu(C{=}O)_{quinone}$=1648.18 cm^{-1}, ν(C=C) =1608.97cm^{-}

[1] , δ(N-H)*$_{lactam,substituent}$ =1576.16 cm^{-1} , δ(N-H)$_{carbamate}$ =1487.91 cm^{-1}, ν(C-N)$_{carbamate}$ =1323.17 cm^{-1}, ν(C- O-C)$_{carbamate}$ =1189.91 cm^{-1}, ν(C- O-C)$_{methoxy}$ =1054.32cm^{-1} , γ(N-H)$_{carbamate}$ =783.23 cm^{-1} . ^{1}H NMR (500 MHz, $CDCl_3$) δ 9.14 (s, 1H, 1-NH), 7.30 (s, 1H, 19-H), 6.95 (d, $J^3_{H-3,H-4}$ = 12.1 Hz, 1H, 3-H), 6.57 (t, $J^3_{H-3,H-4}$ = 11.7 Hz, 1H, 4-H), 6.39 (d, $J^3_{NH-17,H-29}$ = 8.4 Hz, 1H, 17-NH), 5.89 (d, $J^3_{H-9,H-10}$ = 7.9 Hz, 1H, 9-H), 5.85 (t, $J^3_{H-4,H-5}$ = 10.9 Hz, 1H, 5-H), 5.18 (s, 1H, 7-H), 4.85 (bs, 2H, 24-NH_2), 4.35 – 4.27 (m, 1H, 6-H), 4.12 – 4.02 (m, 2H, 29-H, 11-OH), 3.74 – 3.62 (m, 1H, 35-H), 3.61 – 3.54 (m, 1H, 11-H), 3.50 – 3.41 (m, 2H, 12,30-H), 3.36 (s, 3H, 27-H), 3.26 (s, 3H, 23-H), 2.99 – 2.85 (m, 1H, 31-H), 2.86 – 2.78 (m, 1H, 31-H), 2.79 – 2.73 (m, 1H, 10-H), 2.73 – 2.71 (m, 1H, 15-H), 2.70 – 2.68 (bs, 2H, 34-H), 2.67 – 2.64 (m, 1H, 35-H), 2.64 – 2.60 (m, 1H, 30-H), 2.19 – 2.10 (m, 1H, 15-H), 2.05 – 1.99 (m, 3H, 22-H), 1.94 – 1.90 (m, 1H, 33-H), 1.78 (s, 5H, 13,25-H), 1.78 – 1.68 (m, 1H, 14-H), 1.68 – 1.57 (m, 1H, 32-H), 1.53 – 1.43 (m, 1H, 32-H), 0.99 (d, $J^3_{H-10,H-26}$ = 7.1 Hz, 3H, 26-H), 0.97 (d, $J^3_{H-14,H-28}$ = 6.7 Hz, 3H, 28-H). ^{13}C NMR (126 MHz, $CDCl_3$) δ 183.9 (18-C), 180.9 (21-C), 168.5 (1-C), 156.2 (24-C), 144.7 (17-C), 141.4 (20-C), 136.0 (5-C), 135.1 (2-C), 133.8 (9-C), 133.0 (8-C), 127.1 (3-C), 126.6 (4-C), 109.1 (16-C), 108.8 (19-C), 81.7 (7-C), 81.5 (12-C), 81.3 (6-C), 72.8 (11-C), 57.3 (23-C), 56.9* (27,30-C), 53.9 (35-C), 50.8 (29-C), 46.6 (31-C), 35.2* (13,34-C), 34.8 (15-C), 32.5 (10-C), 29.4 (14-C), 26.6 (33-C), 23.2 (28-C), 19.5 (32-C), 12.9 (22-C), 12.8 (25-C), 12.5 (26-C).

(1S,4S)-1-Allyl-3-(((4E,6Z,8S,9S,10E,12S,13R,14S,16R)-9-(carbamoyloxy)-13-hydroxy-8,14-dimethoxy-4,10,12,16-tetramethyl-3,20,22-trioxo-2-azabicyclo[16.3.1]docosa-1(21),4,6,10,18-pentaen-19-yl)amino)quinuclidin-1-ium (compound **9**): 50 mg (0.08 mmol) of **8** was dissolved in a 2 mL of ACN and then a four-fold excess of allyl bromide was added (26 μL, 0.31 mmol). The mixtures were stirred at 30 °C for a 24 hours. Then a large amount of diethyl ether (100 mL) was added. The precipitate was filtered on a Büchner funnel. The product

was obtained as a violet powder. Yield 97 %, mp decomposition >250 ºC. Anal. Calcd for $C_{38}H_{55}N_4O_8$: C, 65.59; H, 7.97; N, 8.05. Found: C, 65.58; H, 7.97; N, 8.06; HRMS (ESI-TOF) m/z: $[M + H]^+$ Calcd 695.4014; Found 695.4015. FT-IR (KBr): $\nu_{as}(N\text{-}H)_{carbamate}$=3427.17 cm^{-1}, $\nu_s(N\text{-}H)^*_{carbamate}$, $\nu_s(N\text{-}H)^*_{lactam}$=3349.69$cm^{-1}$, ν(O-H) =3191.67 cm^{-1}, $\nu_s(C\text{-}H)$ =2932.36 cm^{-1}, $\nu(C{=}O)_{carbamate}$ =1718.55 cm^{-1}, $\nu(C{=}O)_{lactam}$=1690.28 cm^{-1}, $\nu(C{=}O)_{quinone,}$ ν(C=C) =1645.67 cm^{-1}, $\delta(N\text{-}H)^*_{lactam,substituent}$ =1580.01 cm^{-1} , $\delta(N\text{-}H)_{carbamate}$ =1487.45 cm^{-1}, $\nu(C\text{-}N)_{carbamate}$ =1324.96 cm^{-1}, $\nu(C\text{- }O\text{-}C)_{carbamate}$ =1190.59 cm^{-1}, $\nu(C\text{- }O\text{-}C)_{methoxy}$ =1098.28cm^{-1} , $\gamma(N\text{-}H)_{carbamate}$ =782.02 cm^{-1} . ^{1}H NMR (500 MHz, $CDCl_3$) δ 8.95 (s, 1H, 1-NH), 7.07 (s, 1H, 19-H), 6.88 (d, $J^3_{H\text{-}3,H\text{-}4}$ = 11.5 Hz, 1H, 3-H), 6.53 (t, $J^3_{H\text{-}3,H\text{-}4}$ = 11.2 Hz, 1H, 4-H), 6.35 (d, $J^3_{NH\text{-}17,H\text{-}29}$ = 7.5 Hz, 1H, 17-NH), 6.03 – 5.94 (m, 1H, 37-H), 5.84 (t, $J^3_{H\text{-}4,H\text{-}5}$ = 10.1 Hz, 1H, 5-H), 5.80 – 5.77 (m, 1H, 9-H), 5.76 – 5.73 (m, 1H, 38-H), 5.68 (d, J^2 = 10.0 Hz, 1H, 38-H), 5.12 (bs, 2H, 24-NH_2), 5.07 (s, 1H, 7-H), 4.78 – 4.69 (m, 1H, 29-H), 4.45 – 4.35 (m, 1H, 30-H), 4.31 – 4.25 (m, 2H, 6, 31-H), 4.22 (d, $J^3_{H\text{-}36,H\text{-}37}$ = 7.2 Hz, 2H, 36-H), 4.18 – 4.05 (m, 2H, 30,31-H), 3.91 (bs, 1H, 35-H), 3.72 – 3.57 (m, 1H, 35-H), 3.56 – 3.48 (m, 1H, 11-H), 3.42 – 3.36 (m, 1H, 12-H), 3.31 (s, 3H, 27-H), 3.25 (s, 3H, 23-H), 2.75 – 2.67 (m, 1H, 10-H), 2.57 (bs, 2H, 15-H), 2.35 (bs, 2H, 32,33-H), 2.24 – 2.09 (m, 3H, 34-H, 11-OH), 1.98 (s, 3H, 22-H), 1.95 (bs, 1H, 32-H), 1.83 – 1.76 (m, 5H, 13,25-H), 1.75 (s, 1H, 14-H), 1.02 (d, $J^3_{H\text{-}10,H\text{-}26}$ = 6.1 Hz, 3H, 26-H), 0.94 (d, $J^3_{H\text{-}14,H\text{-}28}$ = 6.9 Hz, 3H, 28-H). ^{13}C NMR (126 MHz, $CDCl_3$) δ 185.1 (18-C), 180.5 (21-C), 168.4 (1-C), 156.3 (24-C), 145.6 (17-C), 139.8 (20-C), 136.6 (5-C), 134.9 (2-C), 133.7 (9-C), 133.3 (8-C), 129.9 (38-C), 127.2 (3-C), 126.5 (4-C), 124.2 (37-C), 114.1 (16-C), 109.9 (19-C), 82.0 (7-C), 81.6 (12-C), 81.2 (6-C), 72.6 (11-C), 66.1 (36-C), 60.1 (30-C), 57.3 (23-C), 56.9 (27-C), 54.5 (31-C), 53.8 (35-C), 50.2 (29-C), 34.4 (13-C), 34.1 (15-C), 32.4 (10-C), 28.9 (14-C), 27.0 (33-C), 23.1 (28-C), 22.7 (34-C), 18.7 (32-C), 13.0 (22-C), 12.7 (25-C), 12.7 (26-C).

(1S,4S)-1-((E)-But-2-en-1-yl)-3-(((4E,6Z,8S,9S,10E,12S,13R,14S,16R)-9-(carbamoyloxy)-13-hydroxy-8,14-dimethoxy-4,10,12,16-tetramethyl-3,20,22-trioxo-2-azabicyclo[16.3.1]docosa-1(21),4,6,10,18-pentaen-19-yl)amino)quinuclidin-1-ium (compound **10**): 50 mg (0.08 mmol) of **8** was dissolved in a 2 mL of ACN and then a four-fold excess of crotyl bromide was added (30 µL, 0.31 mmol). The mixtures were stirred at 30 °C for a 24 hours. Then a large amount of diethyl ether (100 mL) was added. The precipitate was filtered on a Büchner funnel. The product was obtained as a violet powder. Yield 99 %, mp decomposition >250 ºC. Anal. Calcd for $C_{39}H_{57}N_4O_8$: C, 65.98; H, 8.09; N, 7.89. Found: C, 65.98; H, 8.10; N, 7.88; HRMS (ESI-TOF) m/z: $[M + H]^+$ Calcd 709.4171; Found 709.4169. FT-IR (KBr): $\nu_{as}(N\text{-}H)_{carbamate}$=3426.86 cm^{-1}, $\nu_s(N\text{-}H)*_{carbamate}$, $\nu_s(N\text{-}H)*_{lactam}$=3353.74$cm^{-1}$, ν(O-H) =3187.62 cm^{-1}, $\nu_s(C\text{-}H)$ =2930.33 cm^{-1}, $\nu(C{=}O)_{carbamate}$ =1719.54 cm^{-1}, $\nu(C{=}O)_{lactam}$=1689.89 cm^{-1}, $\nu(C{=}O)_{quinone,}$ ν(C=C) =1645.83 cm^{-1}, $\delta(N\text{-}H)*_{lactam,substituent}$ =1579.89 cm^{-1} , $\delta(N\text{-}H)_{carbamate}$ =1487.37 cm^{-1}, $\nu(C\text{-}N)_{carbamate}$ =1324.47 cm^{-1}, $\nu(C\text{- }O\text{-}C)_{carbamate}$ =1190.56 cm^{-1}, $\nu(C\text{- }O\text{-}C)_{methoxy}$ =1098.38cm^{-1} , $\gamma(N\text{-}H)_{carbamate}$ =782.94 cm^{-1}.^{1}H NMR (500 MHz, $CDCl_3$) δ 8.98 (s, 1H, 1-NH), 7.10 (s, 1H, 19-H), 6.91 (d, $J^3_{H\text{-}3,H\text{-}4}$ = 11.3 Hz, 1H, 3-H), 6.54 (t, $J^3_{H\text{-}3,H\text{-}4}$ = 11.3 Hz, 1H, 4-H), 6.37 (d, $J^3_{NH\text{-}17,H\text{-}29}$ = 8.3 Hz, 1H, 17-NH), 6.26 – 6.17 (m, 1H, 37-H), 5.85 (t, $J^3_{H\text{-}4,H\text{-}5}$ = 10.3 Hz, 1H, 5-H), 5.80 (d, $J^3_{H\text{-}9,H\text{-}10}$ = 9.2 Hz, 1H, 9-H), 5.61 – 5.53 (m, 1H, 38-H), 5.11 (s, 1H, 7-H), 5.03 (bs, 2H, 24-NH_2), 4.76 – 4.69 (m, 1H, 29-H), 4.51 – 4.37 (m, 1H, 30-H), 4.28 (d, $J^3_{H\text{-}36,H\text{-}37}$ = 9.3 Hz, 2H, 36-H), 4.23 – 4.19 (m, 1H, 6-H), 4.18 – 4.13 (m, 2H, 30,31-H), 4.12 – 4.09 (m, 1H, 31-H), 4.09 – 4.02 (m, 1H, 35-H), 3.93 (bs, 1H, 11-OH), 3.63 – 3.49 (m, 2H, 11,35-H), 3.44 – 3.38 (m, 1H, 12-H), 3.32 (s, 3H, 27-H), 3.25 (s, 3H, 23-H), 2.77 – 2.68 (m, 1H, 10-H), 2.57 (d, J^2 = 16.4 Hz, 2H, 15-H), 2.37 – 2.27 (m, 2H, 32-H), 2.16 – 2.11 (m, 2H, 34-H), 1.99 (s, 3H, 22-H), 1.89 – 1.85 (m, 2H, 13-H), 1.81 (d, $J^3_{H\text{-}38,H\text{-}39}$ = 7.4 Hz, 3H, 39-H), 1.76 (s, 3H, 25-H), 1.03 (d, $J^3_{H\text{-}10,H\text{-}26}$ = 6.1 Hz, 3H, 26-H), 0.96 (d, $J^3_{H\text{-}14,H\text{-}28}$ = 6.9 Hz, 3H, 28-H). ^{13}C NMR (126 MHz, $CDCl_3$) δ 185.1 (18-C), 180.6 (21-C), 168.4

(1-C), 156.3 (24-C), 145.5 (17-C), 142.5 (38-C), 139.9 (20-C), 136.6 (5-C), 134.9 (2-C), 133.7 (9-C), 133.2 (8-C), 127.3 (3-C), 126.5 (4-C), 116.9 (37-C), 113.9 (16-C), 109.8 (19-C), 81.9 (7-C), 81.4 (12-C), 81.3 (6-C), 72.7 (11-C), 65.9 (30-C), 59.8 (36-C), 57.3 (23-C), 56.8 (27-C), 54.2 (31-C), 53.5 (35-C), 50.1 (29-C), 34.6 (13-C), 34.1 (15-C), 32.4 (10-C), 29.1 (14-C), 27.1 (33-C), 23.0 (28-C), 22.6 (34-C), 18.6 (32-C), 14.5 (39-C), 13.0 (22-C), 12.7 (25-C), 12.7 (26-C).

(1R,4R)-3-(((4E,6Z,8S,9S,10E,12S,13R,14S,16R)-9-(Carbamoyloxy)-13-hydroxy-8,14-dimethoxy-4,10,12,16-tetramethyl-3,20,22-trioxo-2-azabicyclo[16.3.1]docosa-1(21),4,6,10,18-pentaen-19-yl)amino)-1-((5-nitrofuran-2-yl)methyl)quinuclidin-1-ium (compound **11**): 50 mg (0.08 mmol) of **8** was dissolved in a 2 mL of ACN and then a four-fold excess of 2-(Bromomethyl)-5-nitrofuran was added (63 mg, 0.31 mmol). The mixtures were stirred at 60 °C for a 24 hours. Then a large amount of diethyl ether (100 mL) was added. The precipitate was filtered on a Büchner funnel. The product was obtained as a violet powder. Yield 56 %, mp decomposition >250 ºC. Anal. Calcd for $C_{40}H_{54}N_5O_{11}$: C, 61.52; H, 6.97; N, 8.97. Found: C, 61.51; H, 6.96; N, 8.98; HRMS (ESI-TOF) m/z: $[M + H]^+$ Calcd 780.3814; Found 780.3819. FT-IR (KBr): $\nu_{as}(N\text{-}H)_{carbamate}$=3420.69 cm^{-1}, $\nu_s(N\text{-}H)^*_{carbamate}$, $\nu_s(N\text{-}H)^*_{lactam}$=3343.61$cm^{-1}$, ν(O-H) =3195.72 cm^{-1}, $\nu_s(C\text{-}H)$ =2932.83 cm^{-1}, $\nu(C{=}O)_{carbamate}$ =1718.31 cm^{-1}, $\nu(C{=}O)_{lactam}$=1684.41 cm^{-1}, $\nu(C{=}O)_{quinone}$, ν(C=C) =1645.50 cm^{-1}, $\delta(N\text{-}H)^*_{lactam,substituent}$ =1581.48 cm^{-1} , $\delta(N\text{-}H)_{carbamate}$ =1486.65 cm^{-1}, $\nu(C\text{-}N)_{substituent}$ =1355.90 cm^{-1}, $\nu(C\text{-}N)_{carbamate}$ =1326.03 cm^{-1}, $\nu(C\text{-}O\text{-}C)_{carbamate}$ =1189.56 cm^{-1}, $\nu(C\text{-}O\text{-}C)_{methoxy}$ =1098.07cm^{-1} , $\gamma(N\text{-}H)_{carbamate}$ =782.58 cm^{-1} . ^{1}H NMR (400 MHz, CD_3CN) δ 9.07 (s, 1H, 1-NH), 7.47 (d, $J^3_{H\text{-}39,H\text{-}40}$ = 3.7 Hz, 1H, 39-H), 7.15 (d, $J^3_{H\text{-}39,H\text{-}40}$ = 3.7 Hz, 1H, 40-H), 7.05 (d, $J^3_{H\text{-}3,H\text{-}4}$ = 12.0 Hz, 1H, 3-H), 7.03 (s, 1H, 19-H), 6.62 – 6.54 (m, 1H, 4-H), 6.13 (d, $J^3_{NH\text{-}17,H\text{-}29}$ = 7.9 Hz, 1H, 17-NH), 5.81 (t, $J^3_{H\text{-}4,H\text{-}5}$ = 10.0 Hz, 1H, 5-H), 5.64 (d, $J^3_{H\text{-}9,H\text{-}10}$ = 9.6 Hz, 1H, 9-H), 5.25 (bs, 2H,

24-NH_2), 5.05 (s, 1H. 7-H), 4.76 (s, 2H, 36-H), 4.57 (d, $J^3_{H-29,H-30}$ = 6.6 Hz, 1H, 29-H), 4.44 – 4.39 (m, 2H, 30-H), 4.02 – 3.86 (m, 3H, 31,35-H), 3.60 – 3.45 (m, 3H, 11,12-H, 11-OH), 3.28 (s, 3H, 27-H), 3.21 (s, 3H, 23-H), 2.73 – 2.62 (m, 1H, 10-H), 2.60 – 2.53 (m, 2H, 15-H), 2.36 – 2.28 (m, 3H, 32,33-H), 2.10 – 2.02 (m, 1H, 34-H), 1.95 (s, 3H, 22-H), 1.85 – 1.77 (m, 2H, 13-H), 1.70 (d, $J^3_{H-10,H-25}$ = 1.3 Hz, 3H, 25-H), 1.64 – 1.62 (m, 1H, 14-H), 1.00 (d, $J^3_{H-10,H-26}$ = 6.6 Hz, 3H, 26-H), 0.91 (d, $J^3_{H-14,H-28}$ = 6.9 Hz, 3H, 28-H). ^{13}C NMR (126 MHz, CD_3CN) δ 185.7 (18-C), 181.6 (21-C), 169.9 (1-C), 157.3 (24-C), 154.5 (37-C), 145.7 (17,38-C), 141.7 (20-C), 137.6 (5-C), 134.9 (2-C), 134.3 (9-C), 133.1 (8-C), 129.3 (3-C), 127.0 (4-C), 121.1 (39-C), 113.9 (16-C), 113.4 (40-C), 109.5 (19-C), 82.0 (7-C), 81.9 (12-C), 81.6 (6-C), 73.6 (11-C), 61.1 (36-C), 59.3 (30-C), 57.1 (23-C), 56.7 (27-C), 55.5 (31-C), 55.4 (35-C), 50.5 (29-C), 34.9 (13-C), 34.2 (15-C), 33.4 (10-C), 30.1 (14-C), 27.0 (33-C), 22.9 (28-C), 22.8 (34-C), 18.9 (32-C), 13.5 (22-C), 13.3 (25-C), 12.6 (26-C).

(1S,4S)-1-((2-Bromothiophen-3-yl)methyl)-3-(((4E,6Z,8S,9S,10E,12S,13R,14S,16R)-9-(carbamoyloxy)-13-hydroxy-8,14-dimethoxy-4,10,12,16-tetramethyl-3,20,22-trioxo-2-azabicyclo[16.3.1]docosa-1(21),4,6,10,18-pentaen-19-yl)amino)quinuclidin-1-ium (compound **12**): 50 mg (0.08 mmol) of **8** was dissolved in a 2 mL of ACN and then a four-fold excess of 2-Bromo-3-(bromomethyl)thiophene was added (78 mg, 0.31 mmol). The mixtures were stirred at 60 °C for a 24 hours. Then a large amount of diethyl ether (100 mL) was added. The precipitate was filtered on a Büchner funnel. The product was obtained as a violet powder. Yield 54 %, mp decomposition >250 ºC. Anal. Calcd for $C_{40}H_{54}BrN_4O_8S$: C, 57.82; H, 6.55; Br, 9.62; N, 6.74; S, 3.86. Found: C, 57.81; H, 6.56; Br, 9.62; N, 6.73; S, 3.85; HRMS (ESI-TOF) m/z: $[M + H]^+$ Calcd 829.2840; Found 829.2838. FT-IR (KBr): $\nu_{as}(N\text{-}H)_{carbamate}$=3423.22 cm^{-1}, $\nu_s(N\text{-}H)^*_{carbamate}$, $\nu_s(N\text{-}H)^*_{lactam}$=3353.74cm^{-1}, ν(O-H) =3193.70 cm^{-1}, ν_s(C-H) =2928.31 cm^{-1}, $\nu(C{=}O)_{carbamate}$ =1718.37 cm^{-1}, $\nu(C{=}O)_{lactam}$=1689.69 cm^{-1}, $\nu(C{=}O)_{quinone,}$ ν(C=C)

=1645.15 cm^{-1}, δ(N-H)*$_{lactam,substituent}$ =1580.14 cm^{-1} , δ(N-H)$_{carbamate}$ =1486.12 cm^{-1}, ν(C-N)$_{carbamate}$ =1325.93 cm^{-1}, ν(C-O-C)$_{carbamate}$ =1190.95 cm^{-1}, ν(C-O-C)$_{methoxy}$ =1098.45cm^{-1} , γ(N-H)$_{carbamate}$ =781.89 cm^{-1} . ^{1}H NMR (400 MHz, $CDCl_3$) δ 8.96 (s, 1H, 1-NH), 7.45 (d, $J^3_{H-39,H-40}$ = 5.7 Hz, 1H, 39-H), 7.39 (d, $J^3_{H-39,H-40}$ = 5.7 Hz, 1H, 40-H), 7.10 (s, 1H, 19-H), 6.90 (d, $J^3_{H-3,H-4}$ = 11.6 Hz, 1H, 3-H), 6.58 – 6.50 (m, 1H, 4-H), 6.37 (d, $J^3_{NH-17,H-29}$ = 7.7 Hz, 1H, 17-NH), 5.84 (t, $J^3_{H-4,H-5}$ = 10.1 Hz, 1H, 5-H), 5.81 (d, $J^3_{H-9,H-10}$ = 6.7 Hz, 1H, 9-H), 5.12 (s, 1H, 7-H), 5.00 (bs, 2H, 24-NH_2), 4.82 (q, $J^3_{H-29,H-30}$ = 13.5 Hz, 1H, 29-H), 4.76 – 4.70 (m, 2H, 36-H), 4.59 (d, J^2= 12.8 Hz, 1H, 30-H), 4.43 – 4.35 (m, 1H, 31-H), 4.30 – 4.26 (m, 2H, 6,30-H), 4.20 (t, J^2= 11.5 Hz, 1H, 31-H), 3.96 (bs, 1H, 35-H), 3.75 – 3.65 (m, 1H, 35-H), 3.64 – 3.52 (m, 1H, 11-OH, 11-H), 3.44 – 3.38 (m, 1H, 12-H), 3.33 (s, 3H, 27-H), 3.26 (s, 3H, 23-H), 2.78 – 2.69 (m, 1H, 10-H), 2.65 – 2.55 (m, 2H, 15-H), 2.40 – 2.35 (m, 1H, 32-H), 2.35 – 2.29 (m, 1H, 33-H), 2.20 – 2.06 (m, 2H, 34-H), 2.02 – 1.98 (m, 3H, 22-H), 1.93 (bs, 3H, 13,32-H), 1.86 – 1.81 (m, 1H, 14-H), 1.77 ($J^4_{H-11,H-25}$ = 1.3 Hz, 3H, 25-H), 1.05 (d, $J^3_{H-10,H-26}$ = 6.5 Hz, 3H, 26-H), 0.97 (d, $J^3_{H-14,H-28}$ = 6.9 Hz, 3H, 28-H). ^{13}C NMR (101 MHz, $CDCl_3$) δ 185.2 (18-C), 180.6 (21-C), 168.4 (1-C), 156.3 (24-C), 145.3 (17-C), 139.8 (20-C), 136.4 (5-C), 134.9 (2-C), 133.8 (9-C), 133.2 (8-C), 131.3 (37-C), 128.2 (39-C), 127.5 (40-C), 127.2 (3-C), 126.5 (4-C), 119.7 (38-C), 114.1 (16-C), 109.8 (19-C), 81.9 (7-C), 81.4 (12-C), 81.2 (6-C), 72.6 (11-C), 60.3 (36-C), 60.3 (30-C), 57.3 (23-C), 56.9 (27-C), 54.6 (31-C), 53.9 (35-C), 50.2 (29-C), 34.5 (13-C), 34.2 (15-C), 32.3 (10-C), 29.0 (14-C), 26.6 (33-C), 23.0 (28-C), 22.7 (34-C), 18.7 (32-C), 13.0 (22-C), 12.7 (25-C), 12.6 (26-C).

(1S,4S)-3-(((4E,6Z,8S,9S,10E,12S,13R,14S,16R)-9-(Carbamoyloxy)-13-hydroxy-8,14-dimethoxy-4,10,12,16-tetramethyl-3,20,22-trioxo-2-azabicyclo[16.3.1]docosa-1(21),4,6,10,18-pentaen-19-yl)amino)-1-cinnamylquinuclidin-1-ium (compound **13**): 50 mg

(0.08 mmol) of **8** was dissolved in a 2 mL of ACN and then a four-fold excess of 3-Bromo-1-phenyl-1-propene was added (84 mg, 0.31 mmol). The mixtures were stirred at 60 °C for a 24 hours. Then a large amount of diethyl ether (100 mL) was added. The precipitate was filtered on a Büchner funnel. The product was obtained as a violet powder. Yield 68 %, mp decomposition >250 ºC. Anal. Calcd for $C_{44}H_{59}N_4O_8$: C, 68.46; H, 7.70; N, 7.26. Found: C, 68.46; H, 7.69; N, 7.27; HRMS (ESI-TOF) m/z: $[M + H]^+$ Calcd 771.4327; Found 771.4324. FT-IR (KBr): $\nu_{as}(N\text{-}H)_{carbamate}$=3424.73 cm^{-1}, $\nu_s(N\text{-}H)^*_{carbamate}$, $\nu_s(N\text{-}H)^*_{lactam}$=3355.77$cm^{-1}$, $\nu(O\text{-}H)$ =3185.59 cm^{-1}, $\nu_s(C\text{-}H)$ =2936.41 cm^{-1}, $\nu(C{=}O)_{carbamate}$ =1719.92 cm^{-1}, $\nu(C{=}O)_{lactam}$=1689.71 cm^{-1}, $\nu(C{=}O)_{quinone,}$ $\nu(C{=}C)$ =1645.82 cm^{-1}, $\delta(N\text{-}H)^*_{lactam,substituent}$ =1579.76 cm^{-1} , $\delta(N\text{-}H)_{carbamate}$ =1486.33 cm^{-1}, $\nu(C\text{-}N)_{carbamate}$ =1324.47 cm^{-1}, $\nu(C\text{-}O\text{-}C)_{carbamate}$ =1190.68 cm^{-1}, $\nu(C\text{-}O\text{-}C)_{methoxy}$ =1098.35cm^{-1} , $\gamma(N\text{-}H)_{carbamate}$ =782.41 cm^{-1} .^{1}H NMR (500 MHz, $CDCl_3$) δ 8.94 (s, 1H, 1-NH), 7.47 – 7.42 (m, 2H, 41,43-H), 7.34 – 7.28 (m, 3H, 40,42,44-H), 7.09 (s, 1H, 19-H), 7.01 (d, $J^3_{H\text{-}37,H\text{-}38}$ = 15.6 Hz, 1H, 38-H), 6.89 (d, $J^3_{H\text{-}3,H\text{-}4}$ = 11.5 Hz, 1H, 3-H), 6.53 (t, $J^3_{H\text{-}3,H\text{-}4}$ = 11.3 Hz, 1H, 4-H), 6.37 (d, $J^3_{NH\text{-}17,H\text{-}29}$ = 7.7 Hz, 1H, 17-NH), 6.26 (dt, $J^3_{H\text{-}37,H\text{-}38}$ = 15.4 Hz, $J^3_{H\text{-}36,H\text{-}37}$ = 7.5 Hz, 1H, 37-H), 5.89 – 5.81 (m, 1H, 5-H), 5.81 (d, $J^3_{H\text{-}9,H\text{-}10}$ = 9.3 Hz, 1H, 9-H), 5.11 (s, 1H, 7-H), 4.97 (bs, 2H, 24-NH_2), 4.76 (q, $J^3_{H\text{-}29,NH\text{-}17}$ = 7.8 Hz, 1H, 29-H), 4.51 – 4.46 (m, 1H, 30-H), 4.44 (d, $J^3_{H\text{-}36,H\text{-}37}$ = 7.6 Hz, 2H, 36-H), 4.30 – 4.26 (m, 1H, 6-H), 4.25 – 4.19 (m, 2H, 30,31-H) , 4.17 (t, J^2 = 11.6 Hz, 1H, 31-H), 3.98 – 3.83 (m, 1H, 35-H), 3.70 – 3.57 (m, 2H, 35-H, 11-OH), 3.54 (s, 1H, 11-H), 3.41 – 3.36 (m, 1H, 12-H), 3.30 (s, 3H, 27-H), 3.25 (s, 3H, 23-H), 2.76 – 2.68 (m, 1H, 10-H), 2.62 – 2.56 (m, 2H, 15-H), 2.38 – 2.28 (m, 2H, 32-H), 2.20 – 2.09 (m, 3H, 33,34-H), 1.98 (s, 3H, 22-H), 1.83 – 1.79 (m, 2H, 13-H), 1.76 (s, 3H, 25-H), 1.73 (s, 1H, 14-H), 1.03 (d, $J^3_{H\text{-}10,H\text{-}26}$ = 6.4 Hz, 3H, 26-H), 0.96 (d, $J^3_{H\text{-}14,H\text{-}28}$ = 6.9 Hz, 3H, 28-H). ^{13}C NMR (101 MHz, $CDCl_3$) δ 185.1 (18-C), 180.6 (21-C), 168.4 (1-C), 156.3 (24-C), 145.5 (17-C), 143.8 (39-C), 139.8 (20-C), 136.4 (5-C), 134.9 (2-C), 134.7 (38-C), 133.7 (9-C), 133.3 (8-C), 129.0 (41,43-C), 127.9 (42-C), 127.5 (40,44-C), 127.2

(3-C), 126.5 (4-C), 113.9 (16,37-C), 109.8 (19-C), 82.0 (7-C), 81.5 (12-C), 81.2 (6-C), 72.7 (11-C), 66.0 (30-C), 60.0 (36-C), 57.3 (23-C), 56.8 (27-C), 54.3 (31-C), 53.8 (35-C), 50.2 (29-C), 34.6 (13-C), 34.1 (15-C), 32.4 (10-C), 29.0 (14-C), 27.1 (33-C), 23.1 (28-C), 22.7 (34-C), 18.7 (32-C), 13.0 (22-C), 12.7 (25-C), 12.7 (26-C).

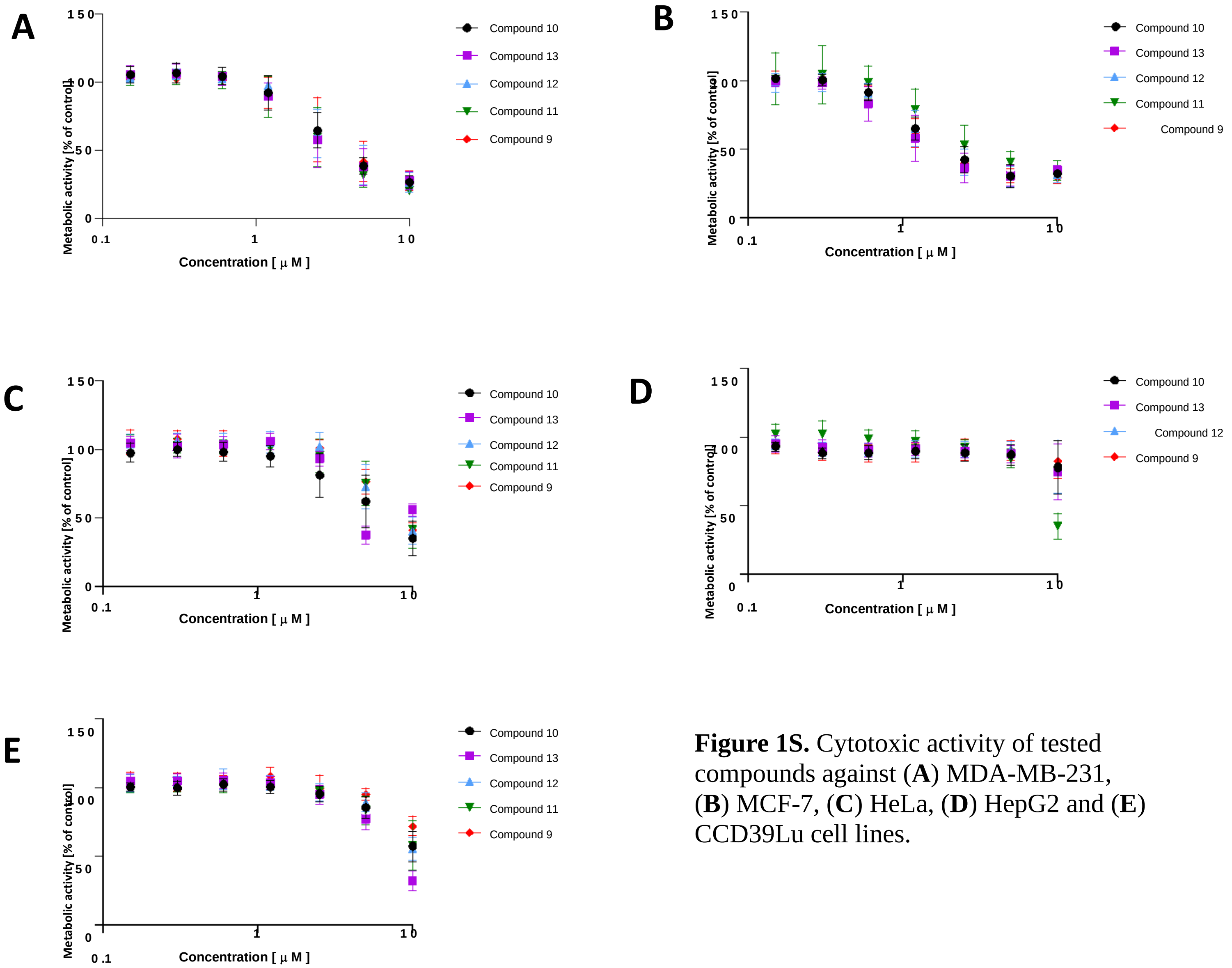

Figure 1S. Cytotoxic activity of tested compounds against (**A**) MDA-MB-231, (**B**) MCF-7, (**C**) HeLa, (**D**) HepG2 and (**E**) CCD39Lu cell lines.

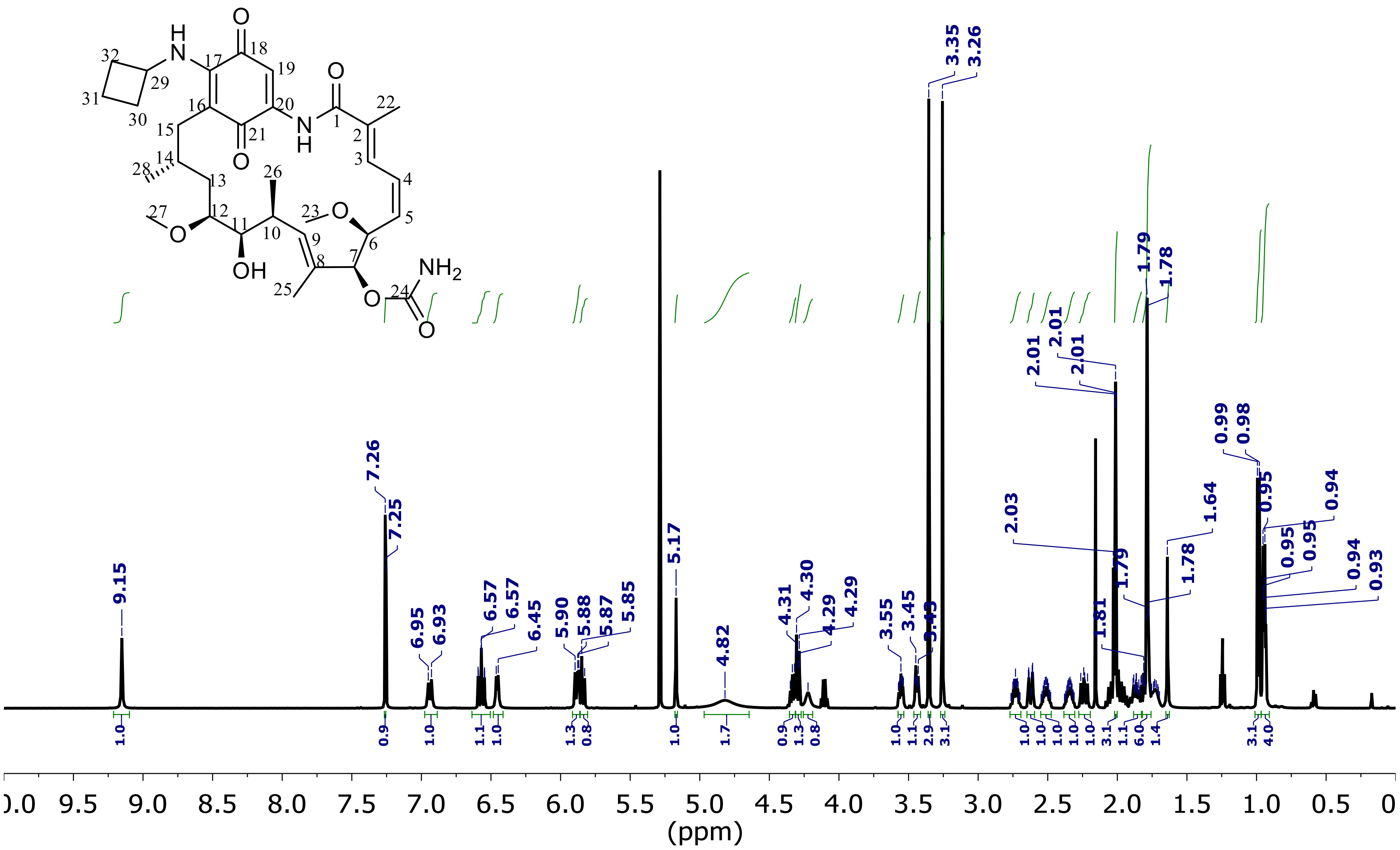

Figure 3S. ^{1}H NMR spectrum of compound **1** in $CDCl_3$.

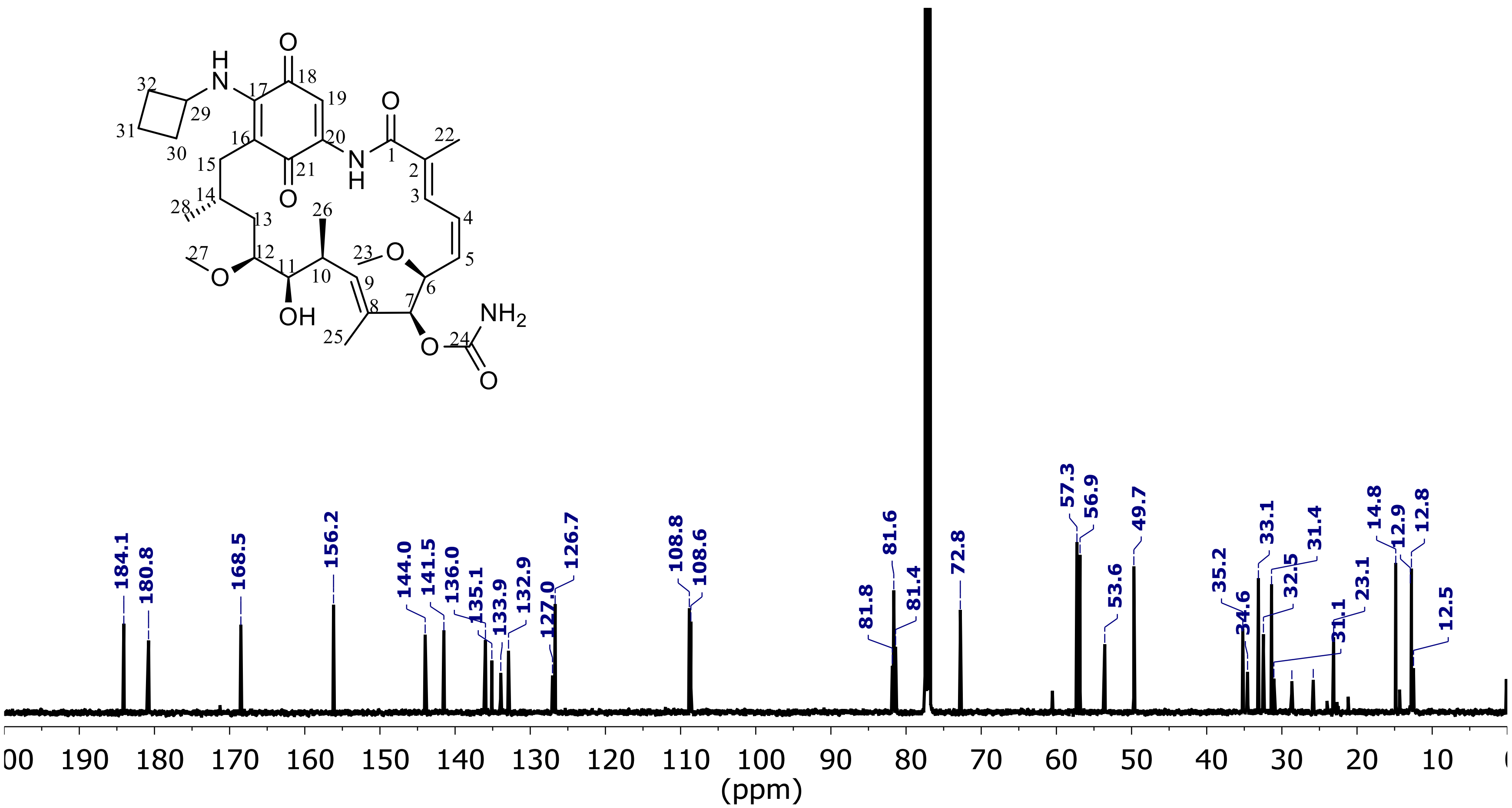

Figure 4S. ^{13}C NMR spectrum of compound **1** in $CDCl_3$.

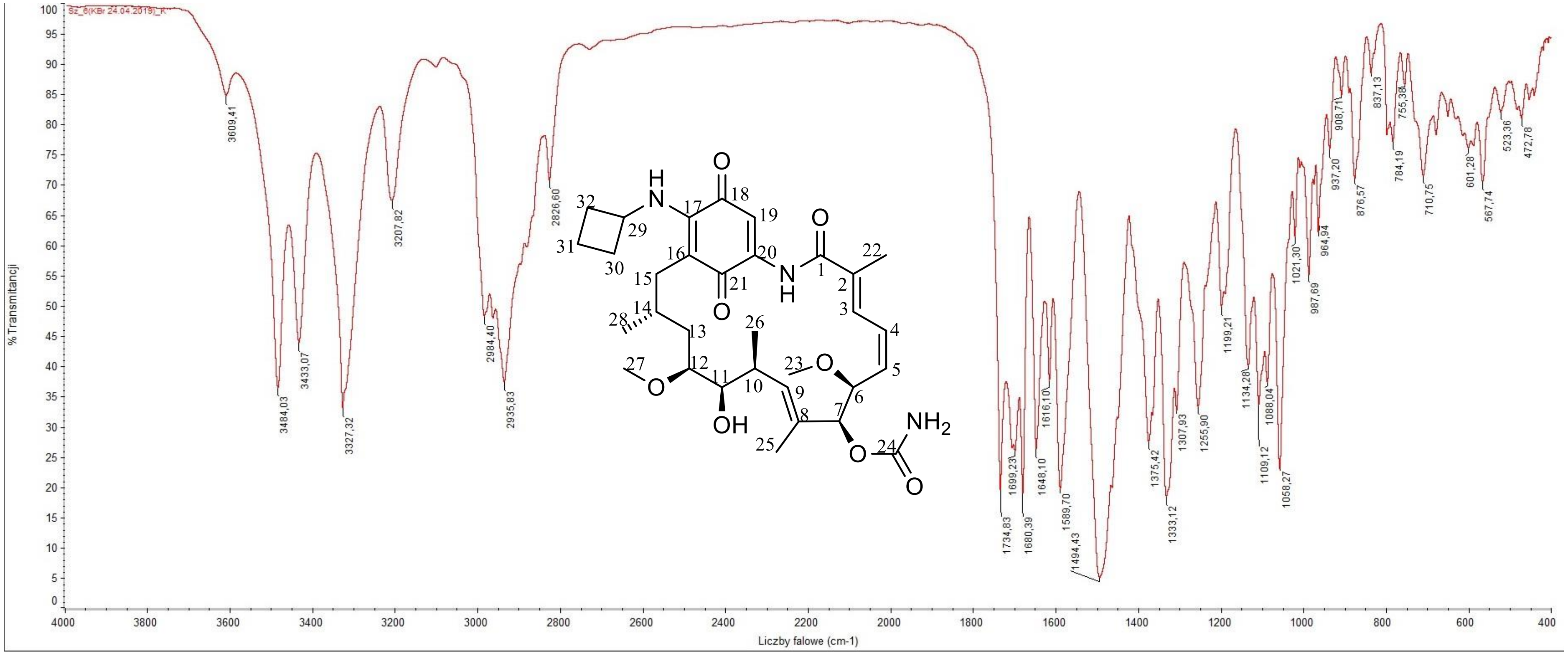

Figure 5S. FT-IR spectrum of compound **1** (in KBr).

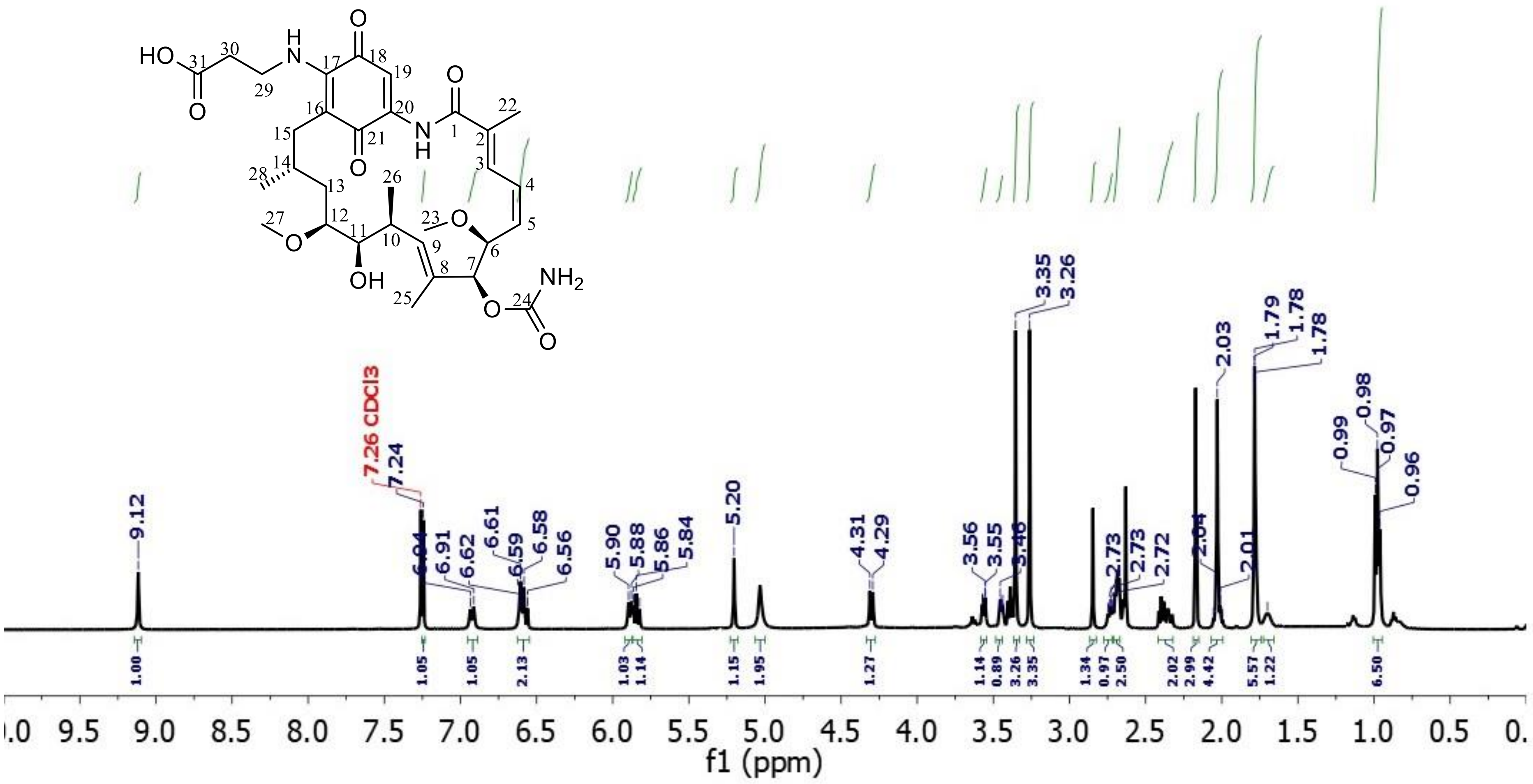

Figure 6S. ^{1}H NMR spectrum of compound **2** in $CDCl_3$.

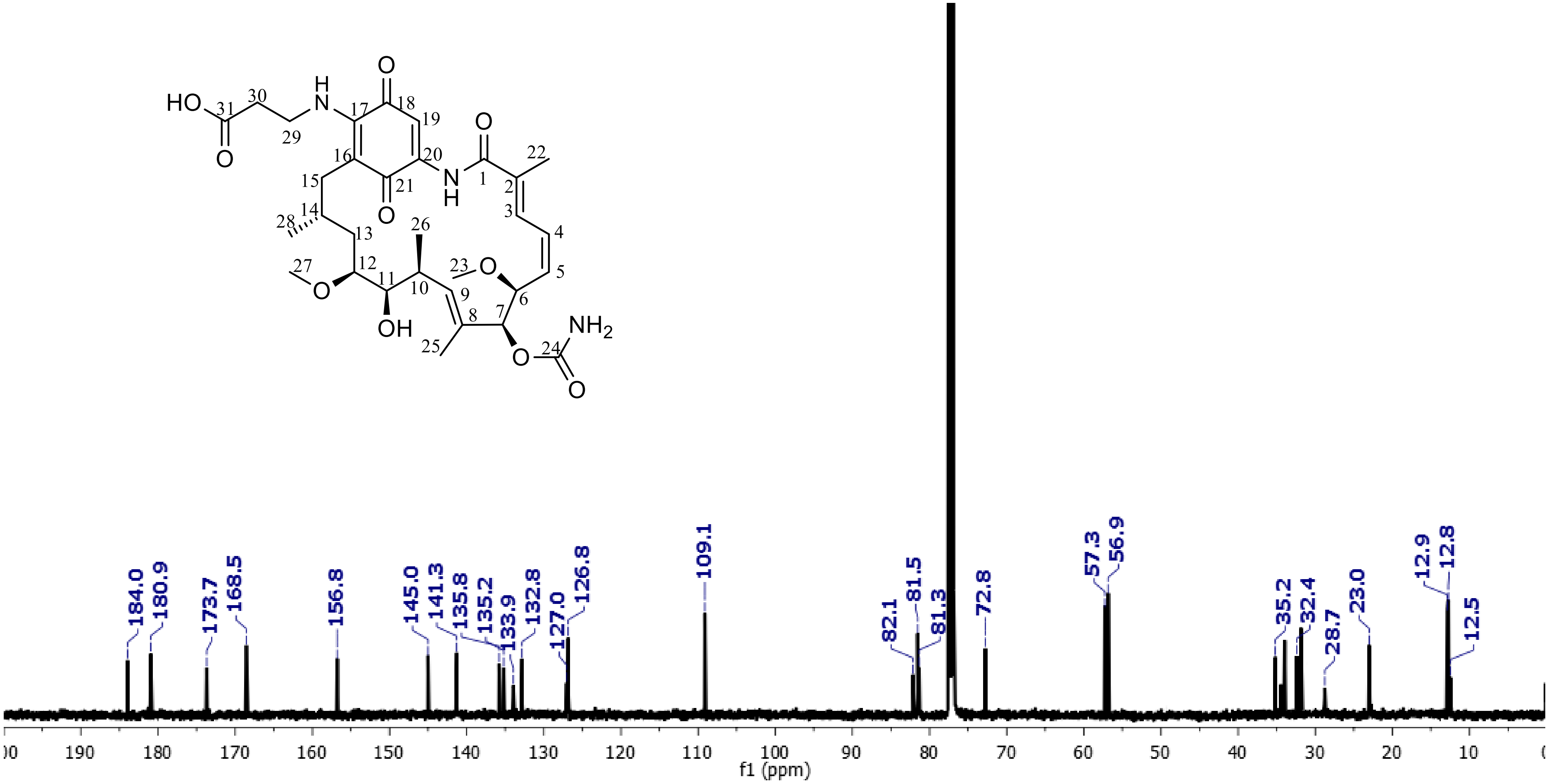

Figure 7S. ^{13}C NMR spectrum of compound **2** in $CDCl_3$.

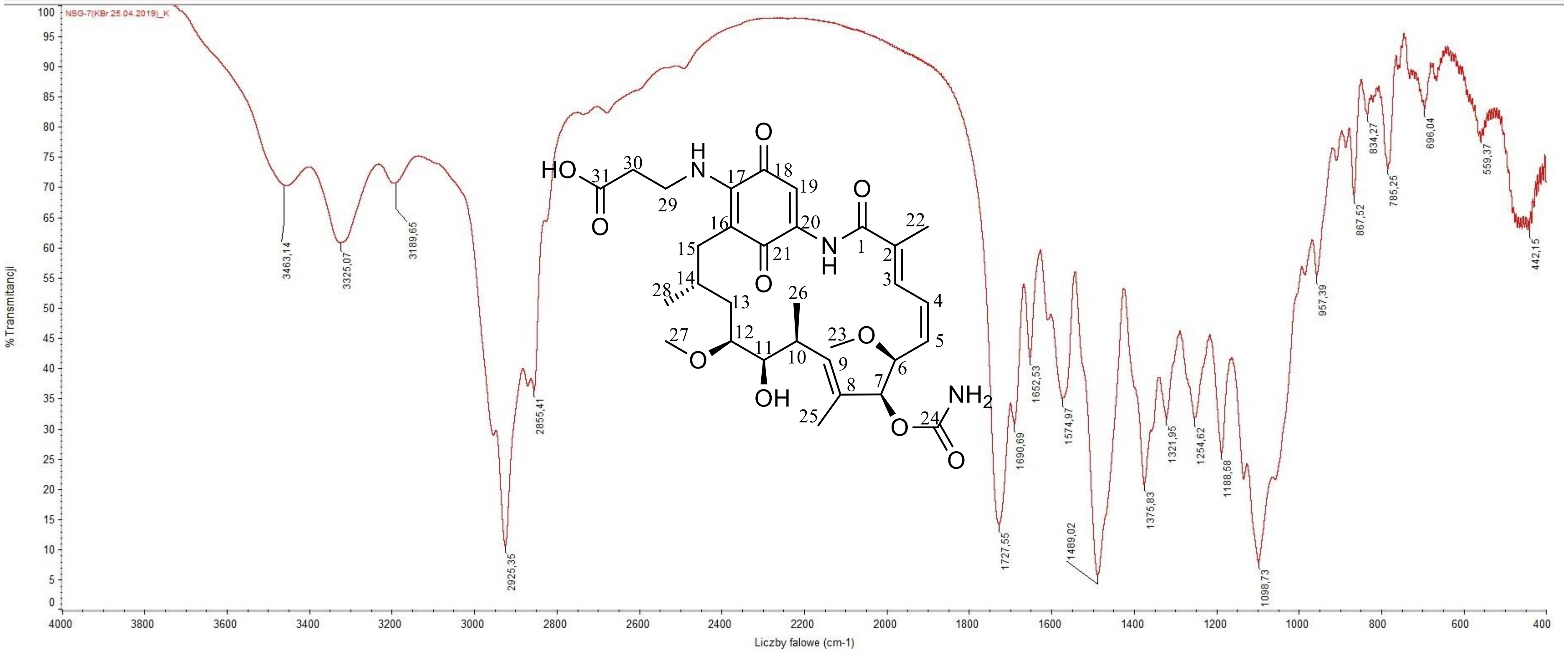

Figure 8S. FT-IR spectrum of compound **2** (in KBr).

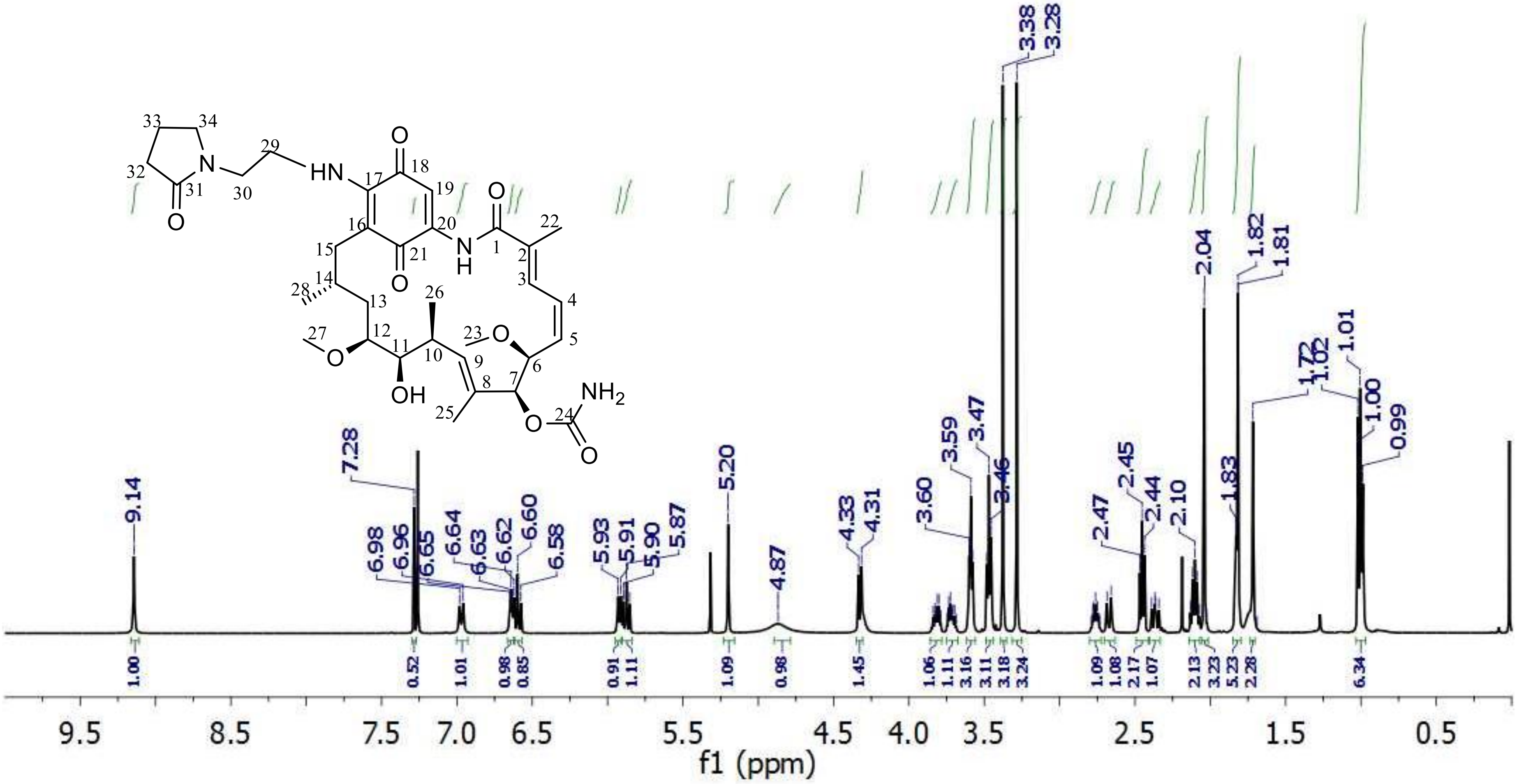

Figure 9S. ^{1}H NMR spectrum of compound **3** (in $CDCl_3$). [5]

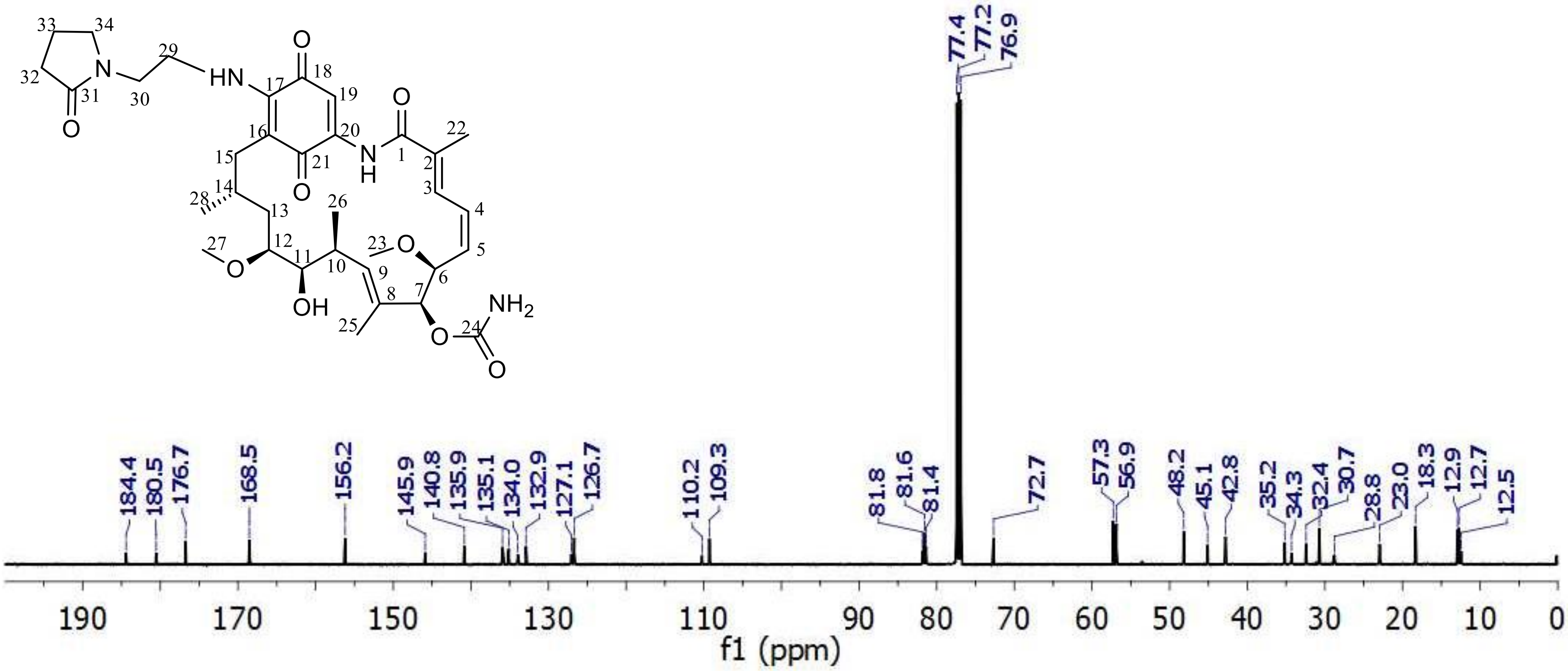

Figure 10S. ^{13}C NMR spectrum of compound **3** (in $CDCl_3$). [5]

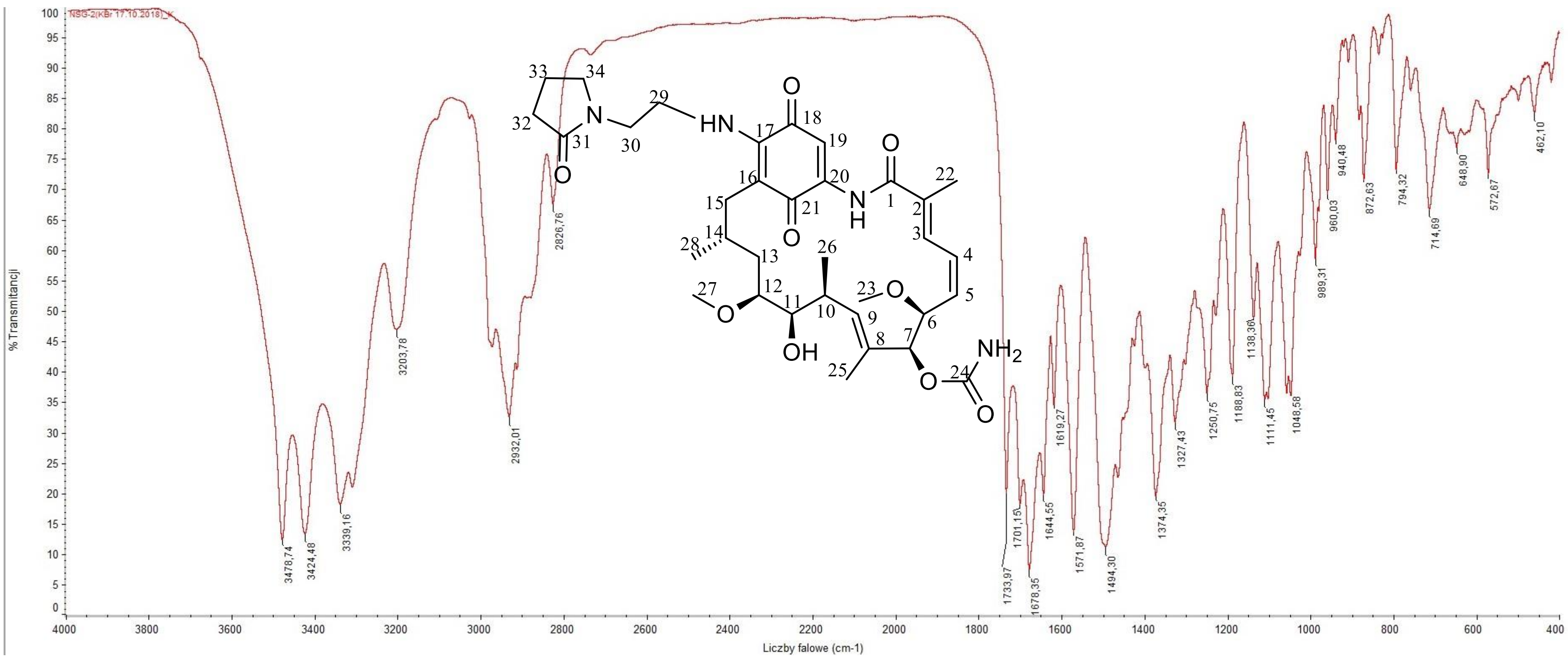

Figure 11S. FT-IR spectrum of compound **3** (in KBr).[5]

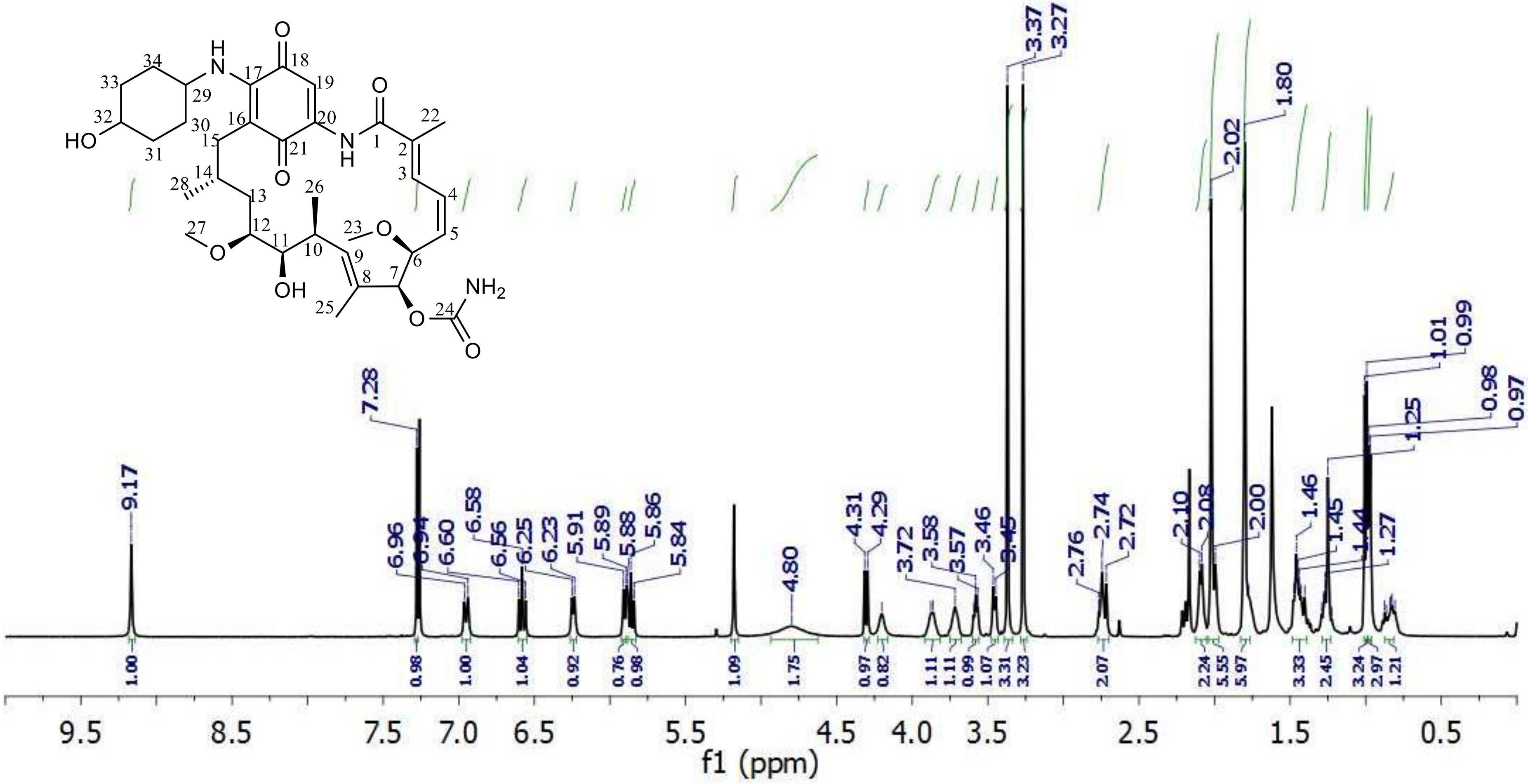

Figure 12S. ^{1}H NMR spectrum of compound **4** (in $CDCl_3$). [5]

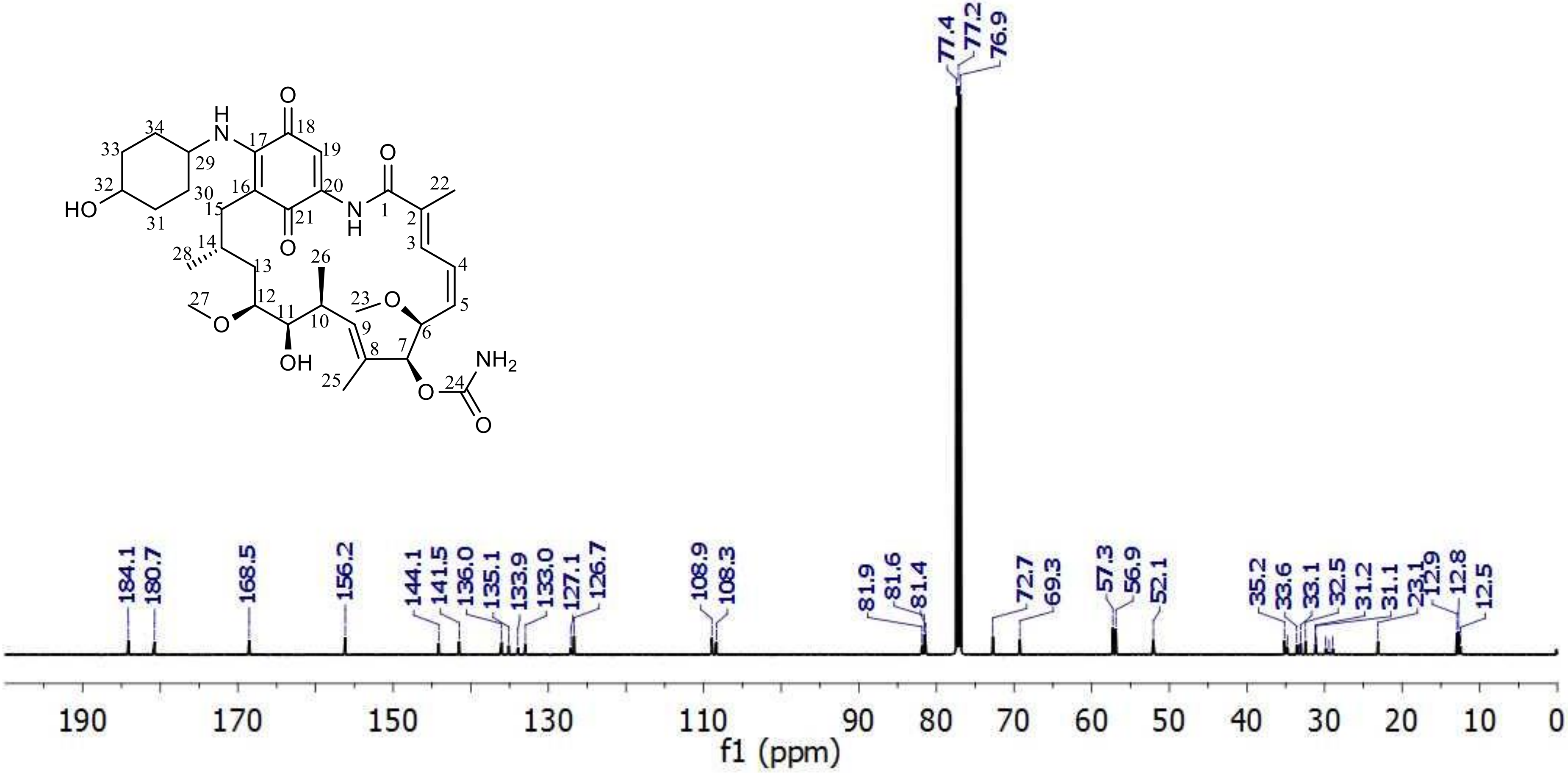

Figure 13S. ^{13}C NMR spectrum of compound **4** (in $CDCl_3$). [5]

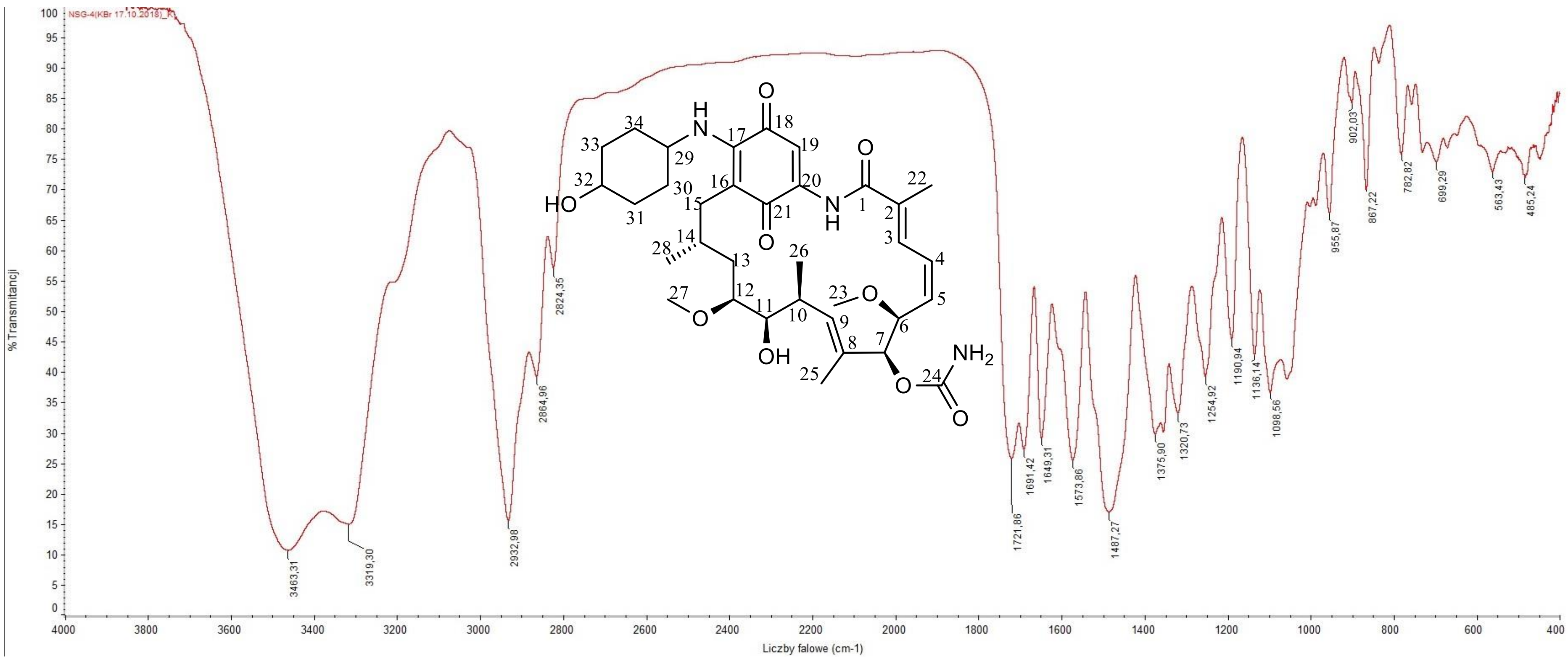

Figure 14S. FT-IR spectrum of compound **4** (in KBr). [5]

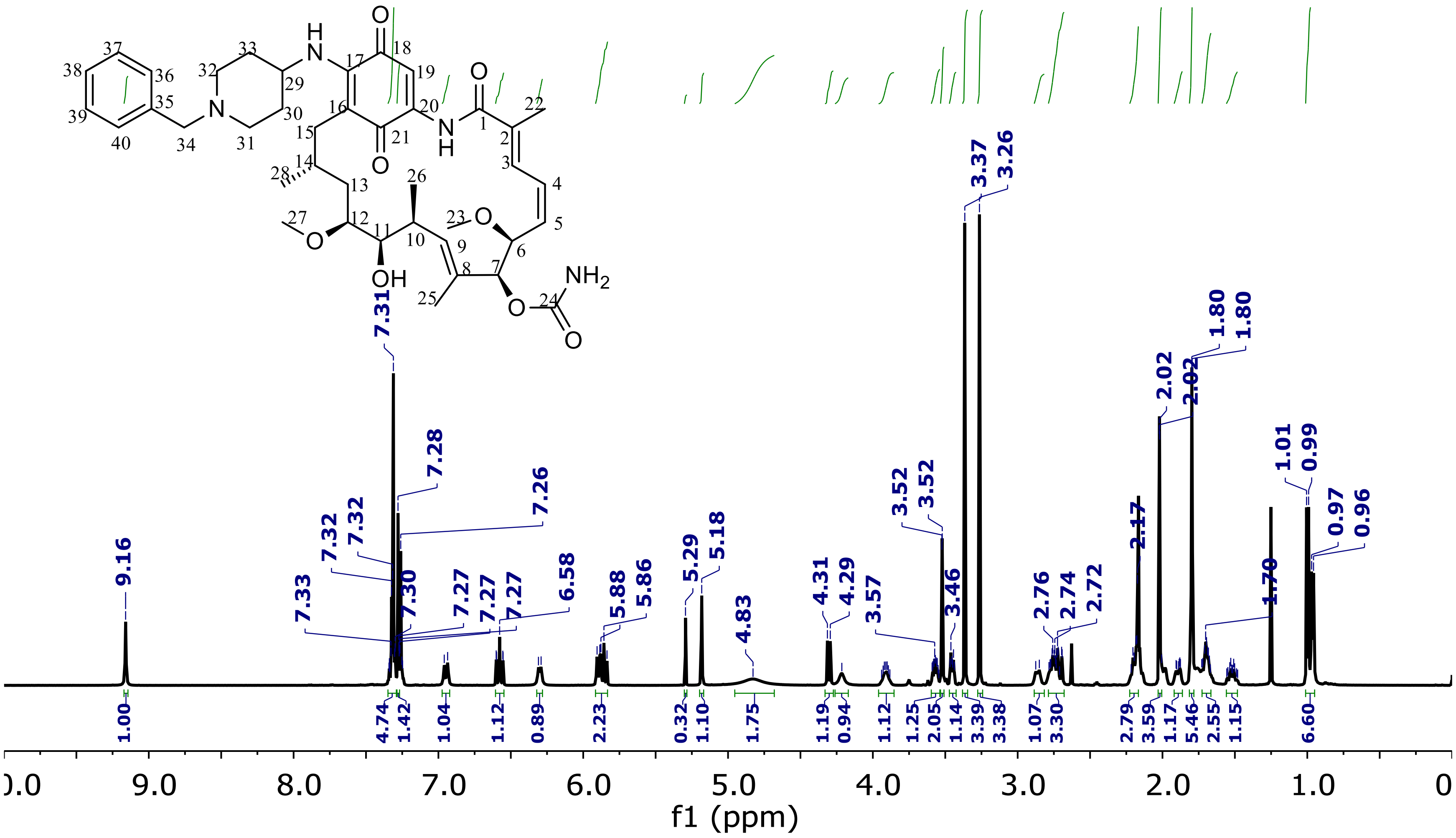

Figure 15S. ^{1}H NMR spectrum of compound **5** (in $CDCl_3$). [5]

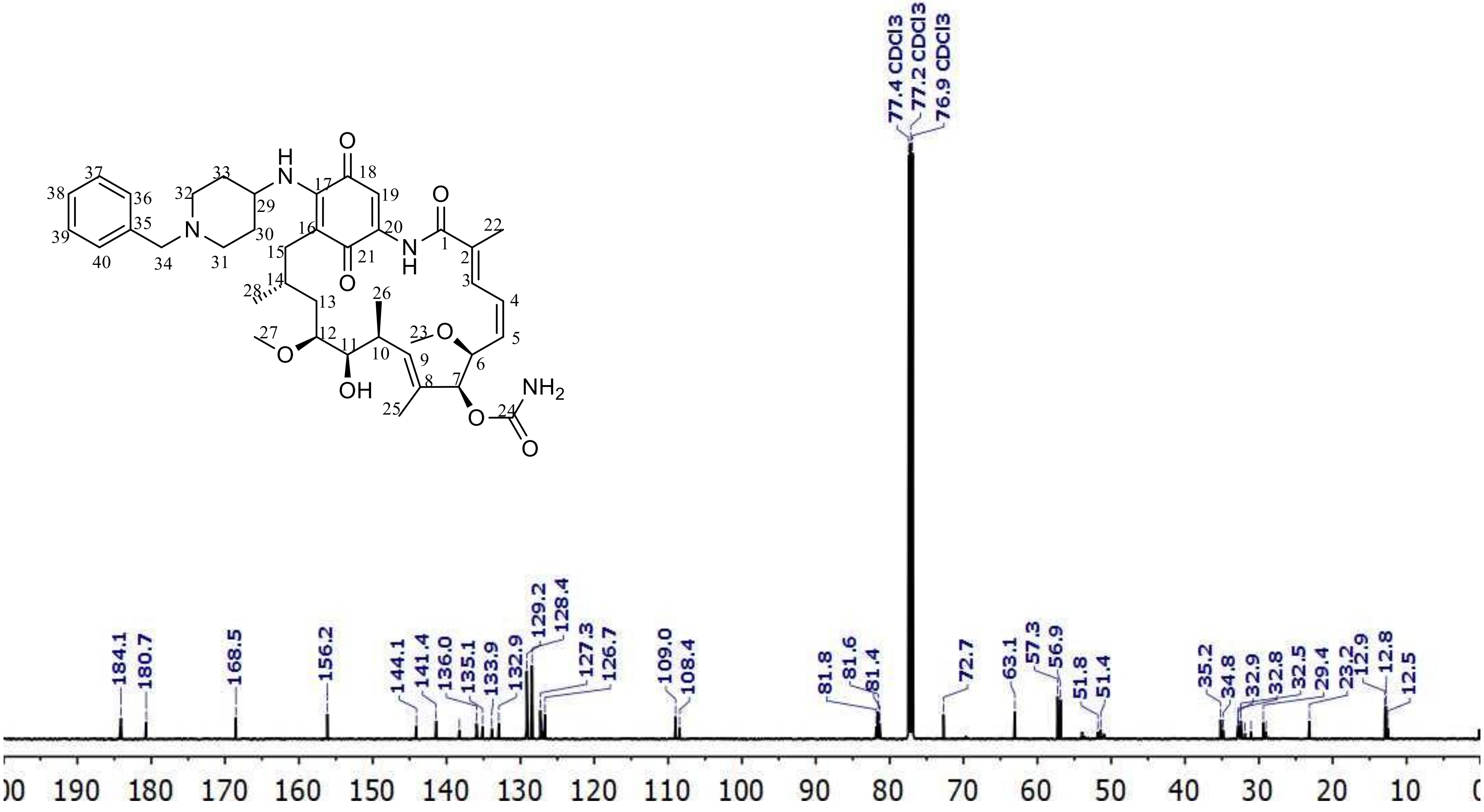

Figure 16S. ^{13}C NMR spectrum of compound **5** (in $CDCl_3$). [5]

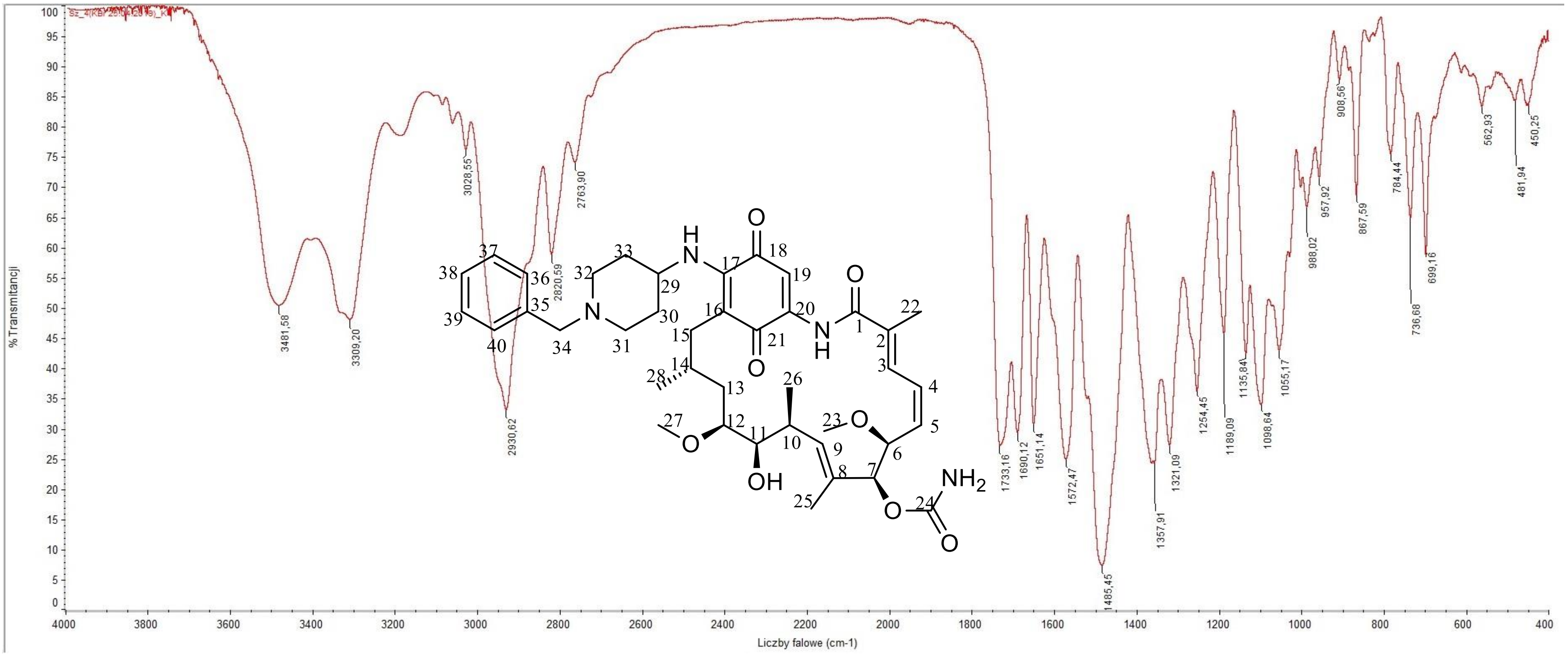

Figure 17S. FT-IR spectrum of compound **5** (in KBr). [5]

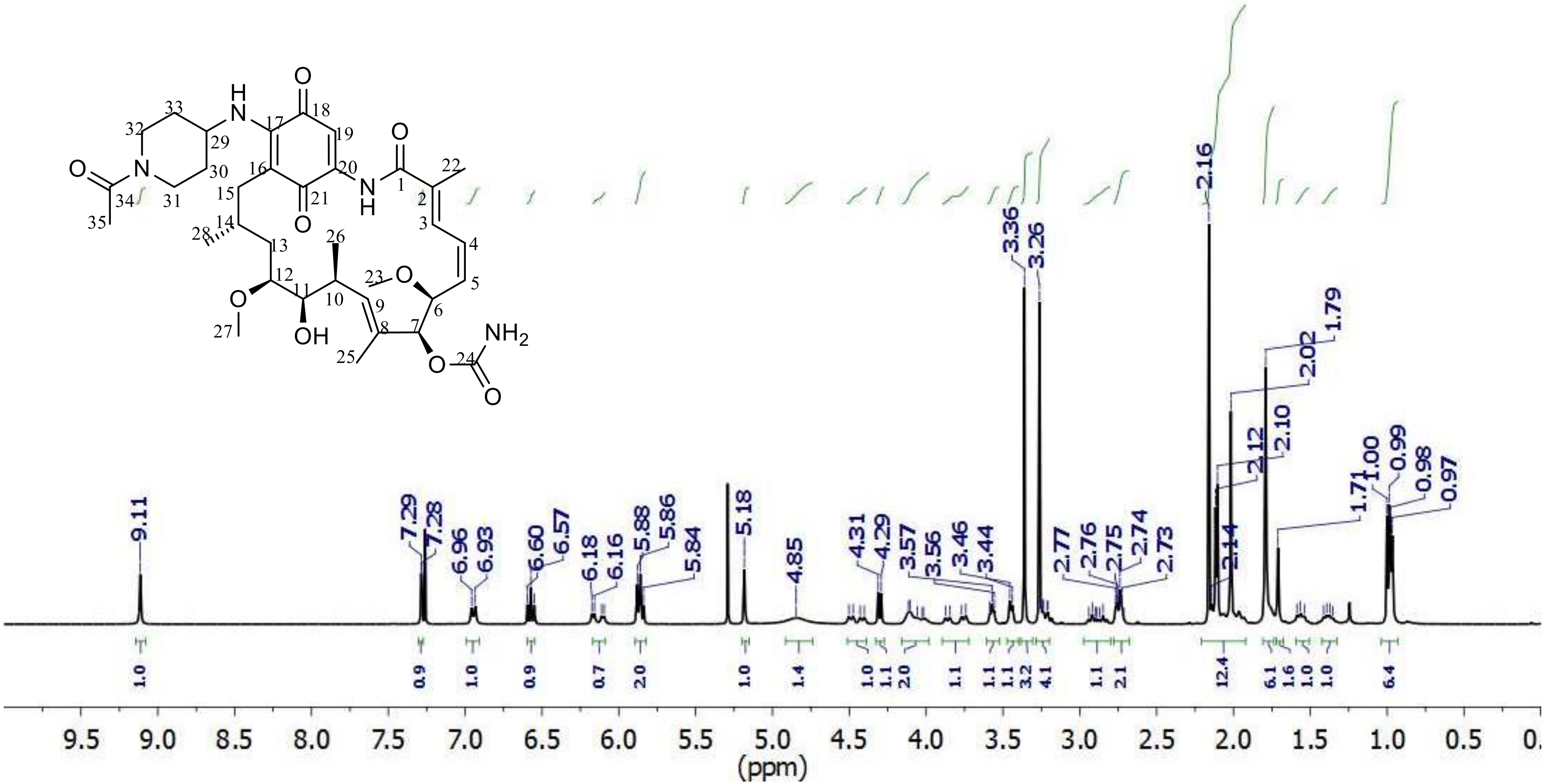

Figure 18S. ^{1}H NMR spectrum of compound **6** (in $CDCl_3$). [5]

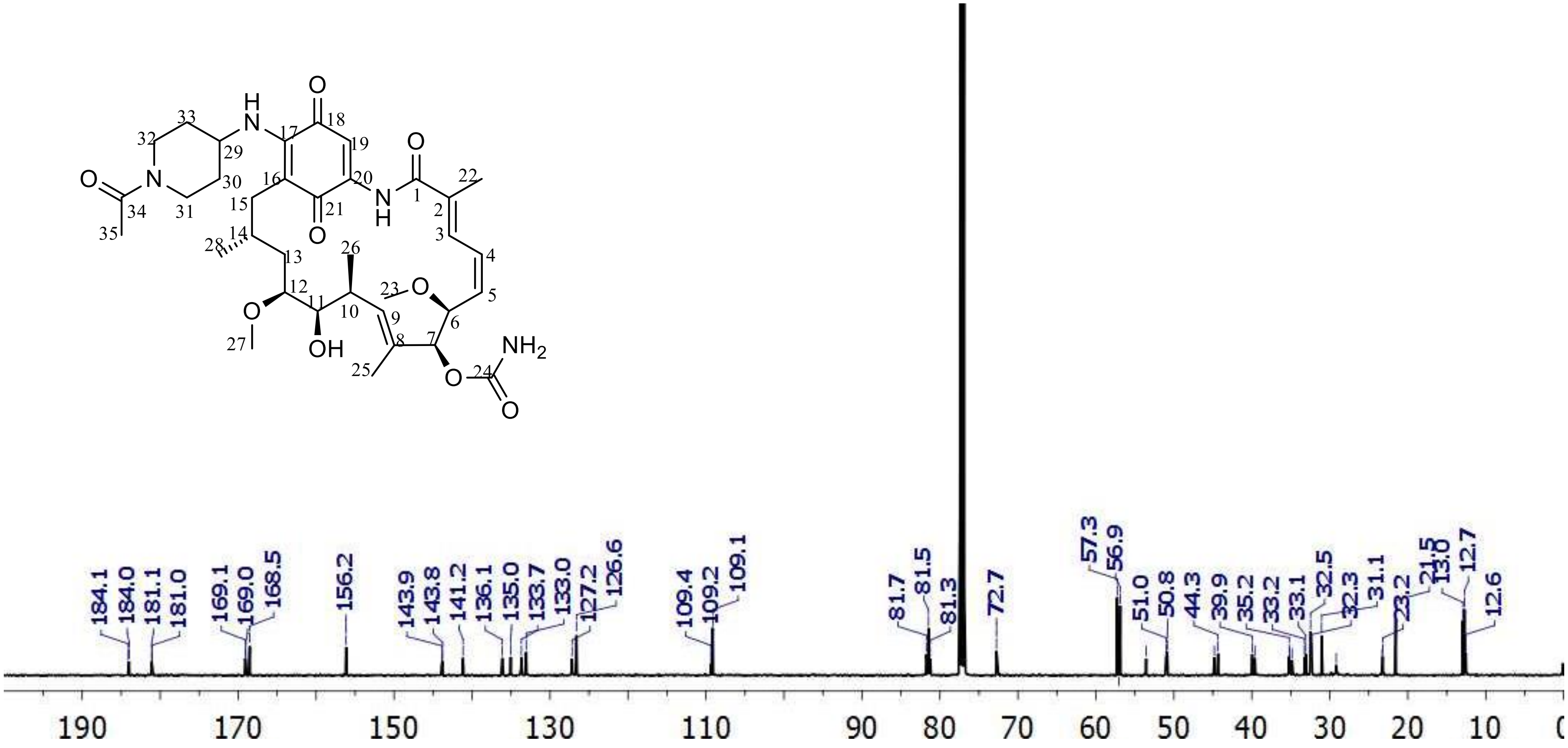

Figure 19S. ^{13}C NMR spectrum of compound **6** (in $CDCl_3$). [5]

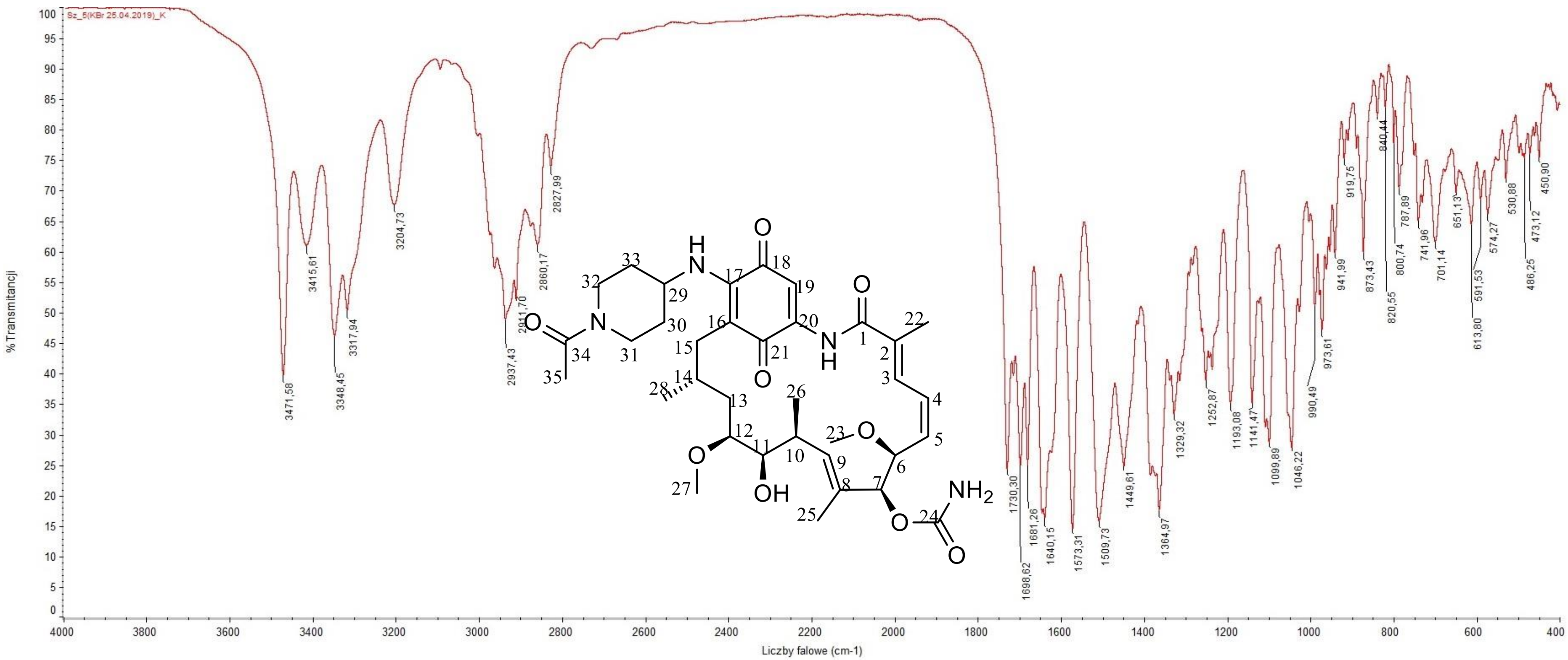

Figure 20S. FT-IR spectrum of compound **6** (in KBr). [5]

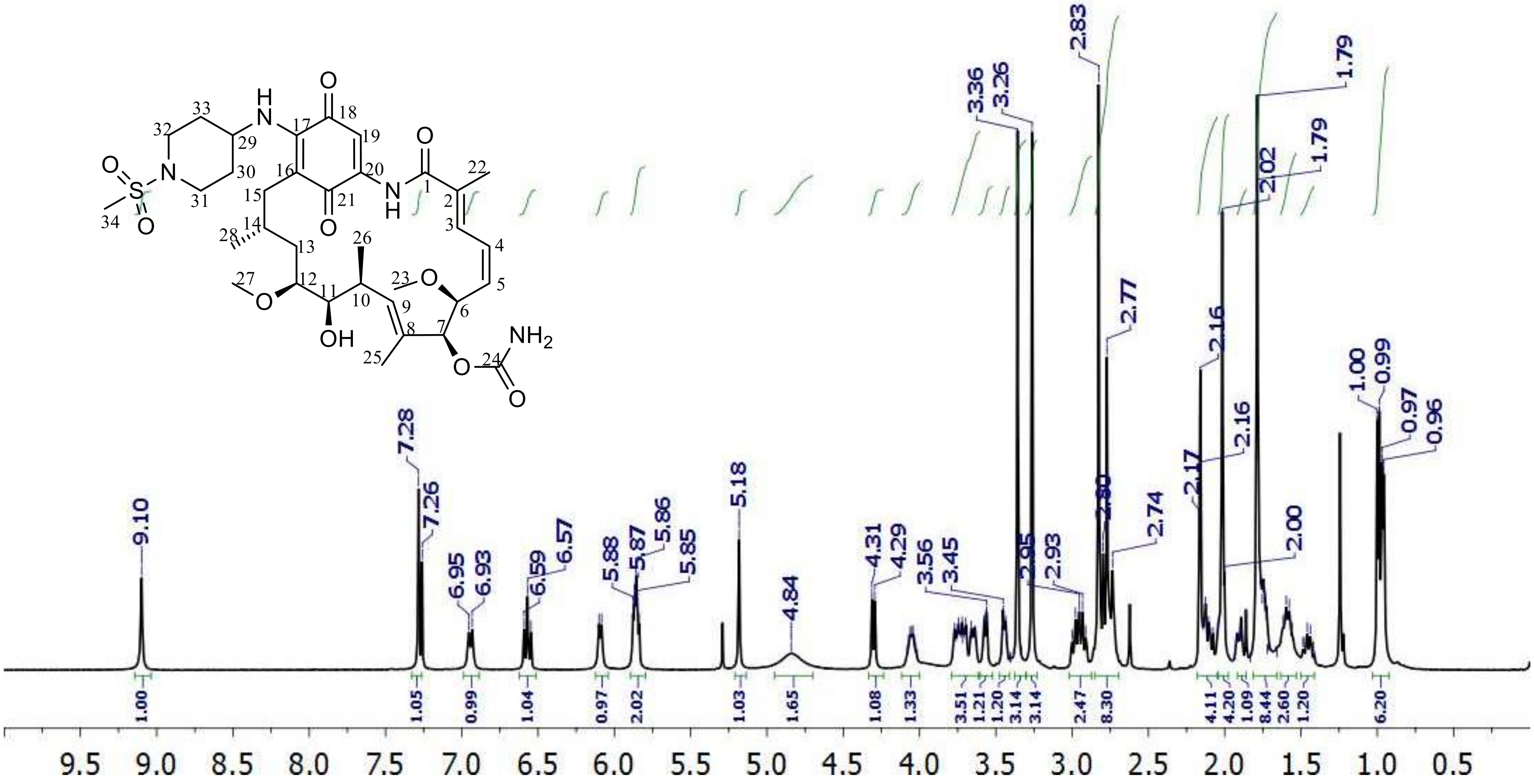

Figure 21S. ^{1}H NMR spectrum of compound **7** (in $CDCl_3$). [5]

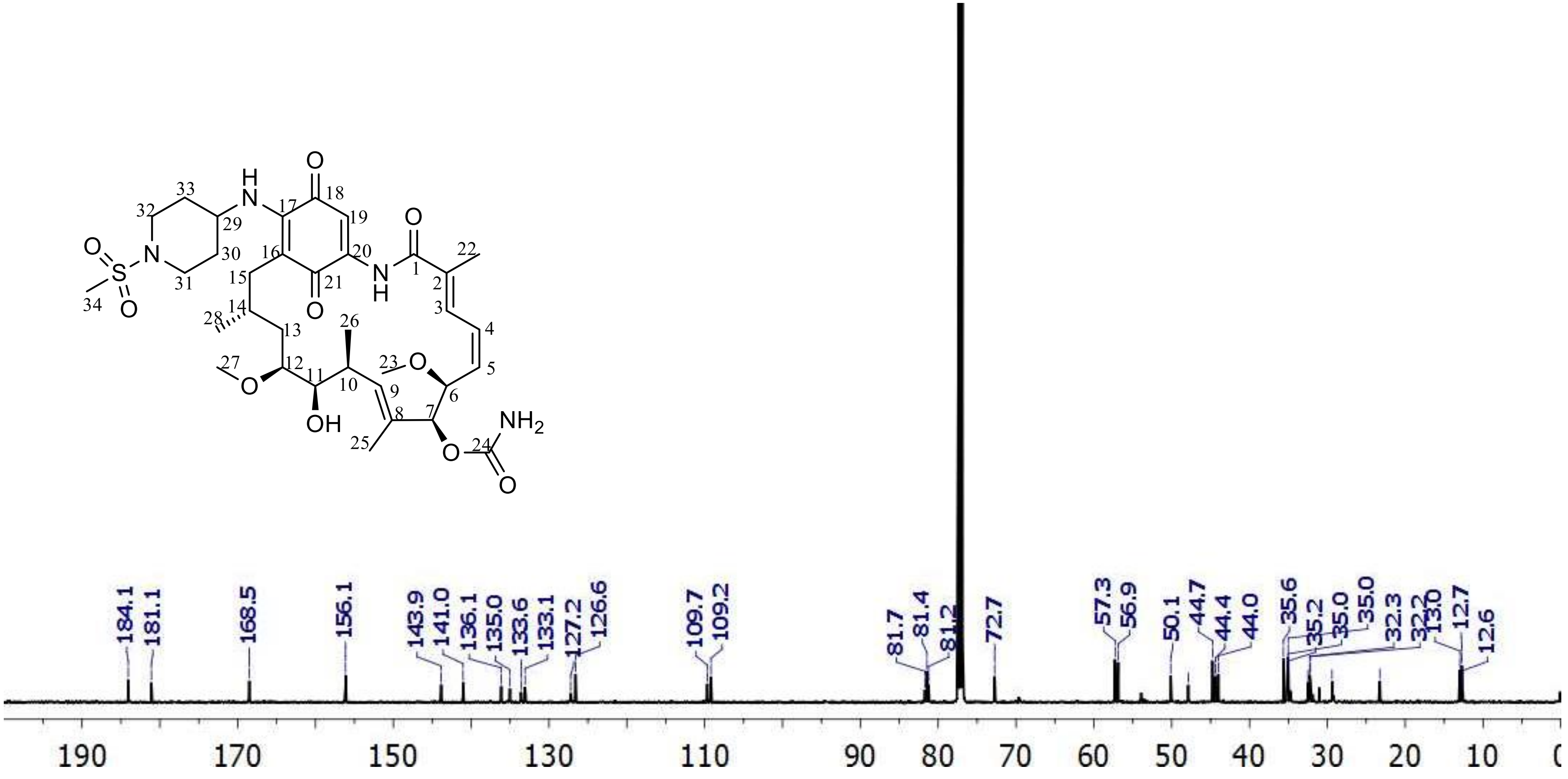

Figure 22S. ^{13}C NMR spectrum of compound **7** (in $CDCl_3$). [5]

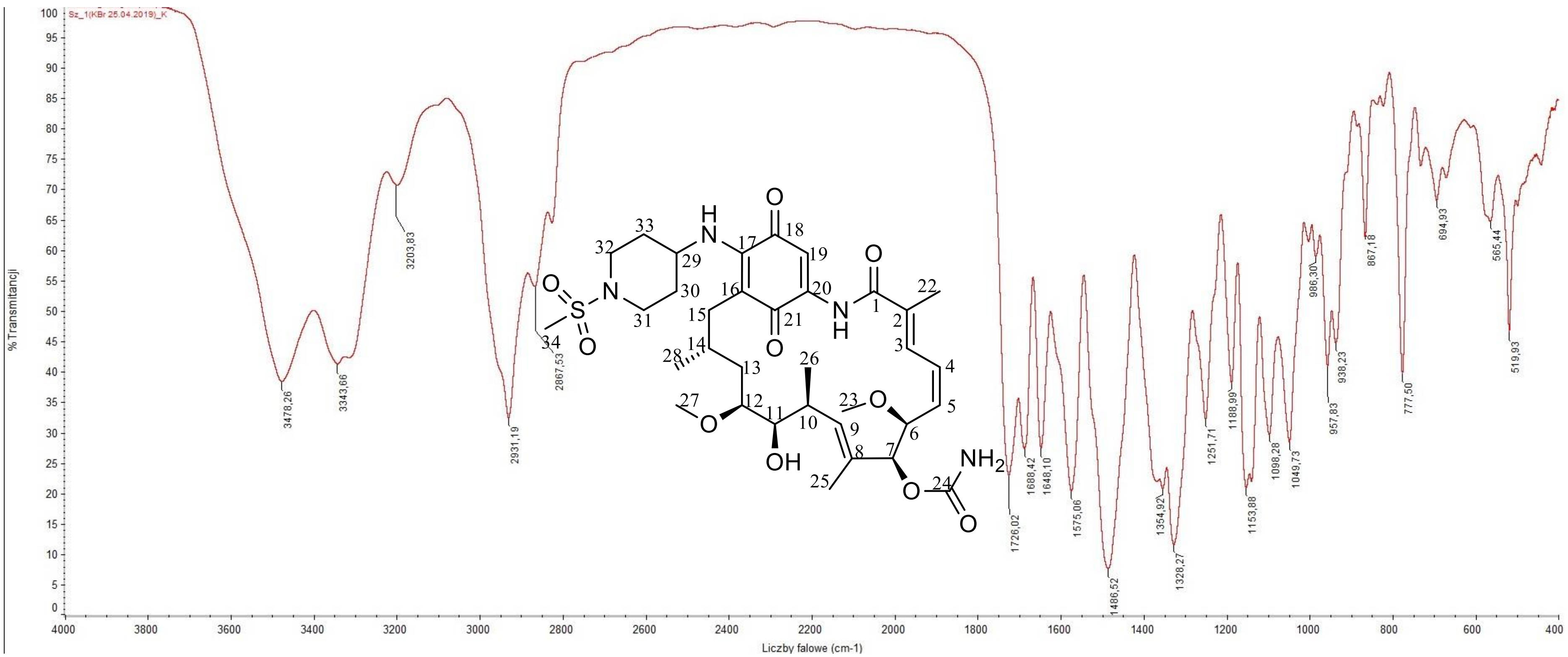

Figure 23S. FT-IR spectrum of compound **7** (in KBr).[5]

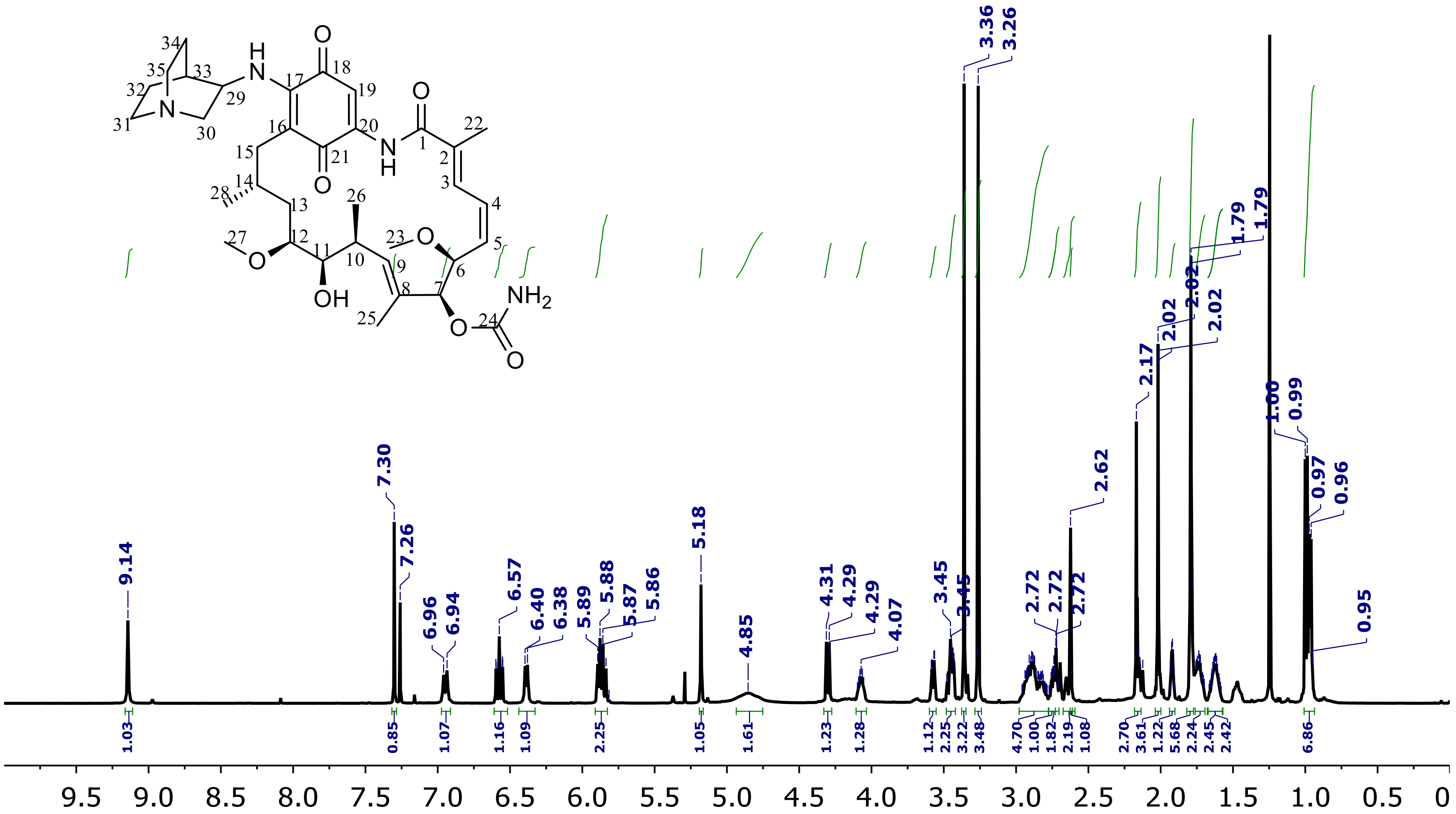

Figure 24S. ^{1}H NMR spectrum of compound **8** in $CDCl_3$.

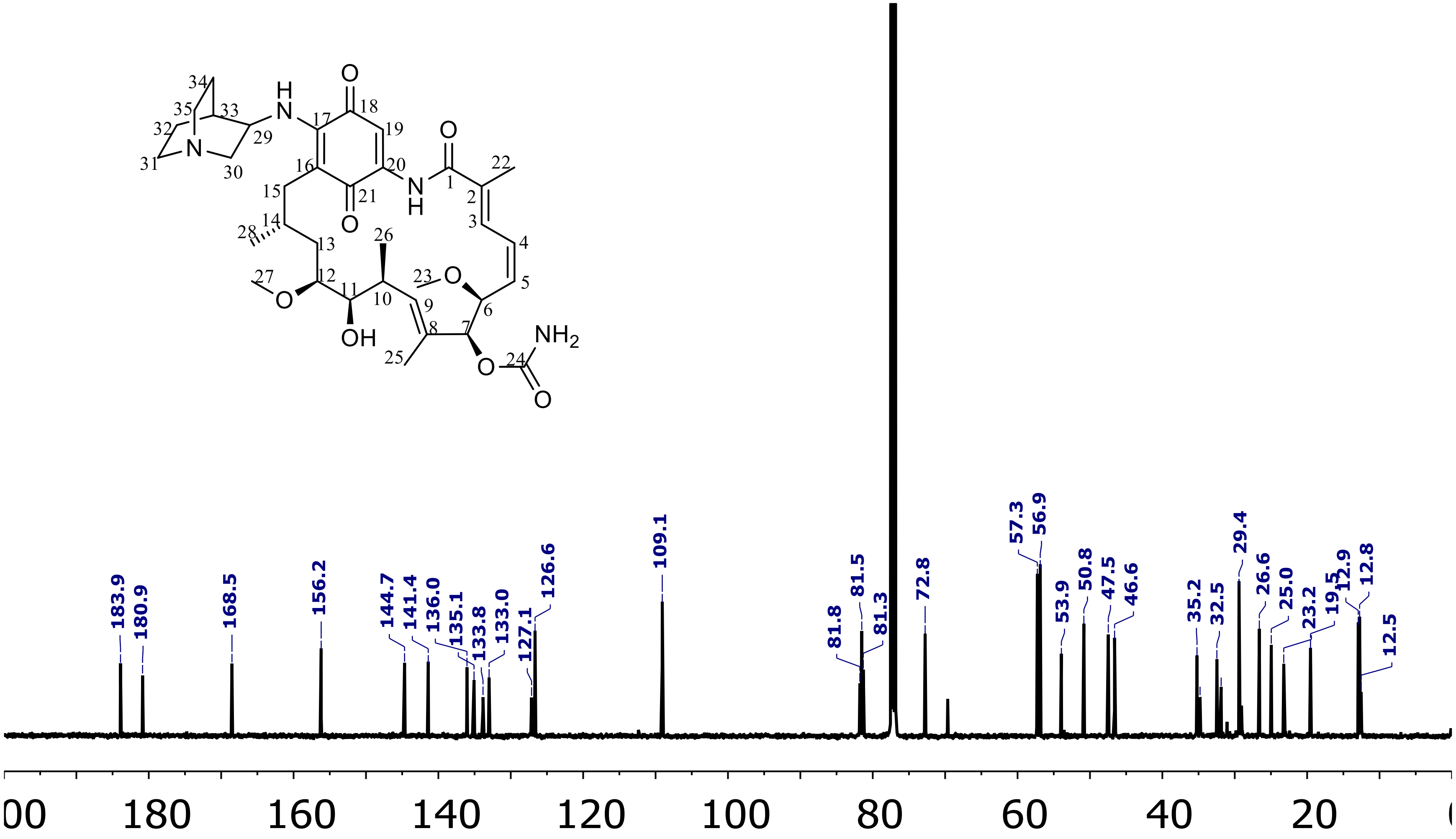

Figure 25S. ^{13}C NMR spectrum of compound **8** in $CDCl_3$.

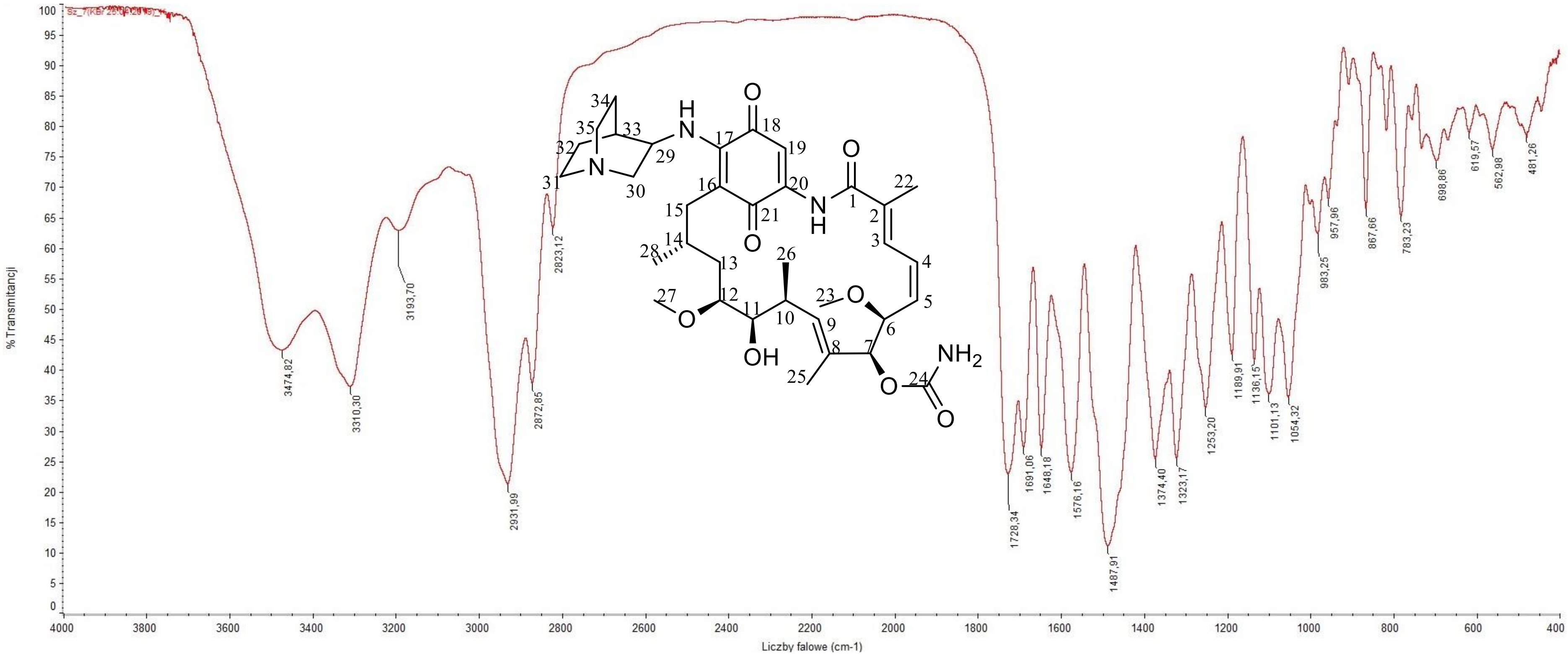

Figure 26S. FT-IR spectrum of compound **8** (in KBr).

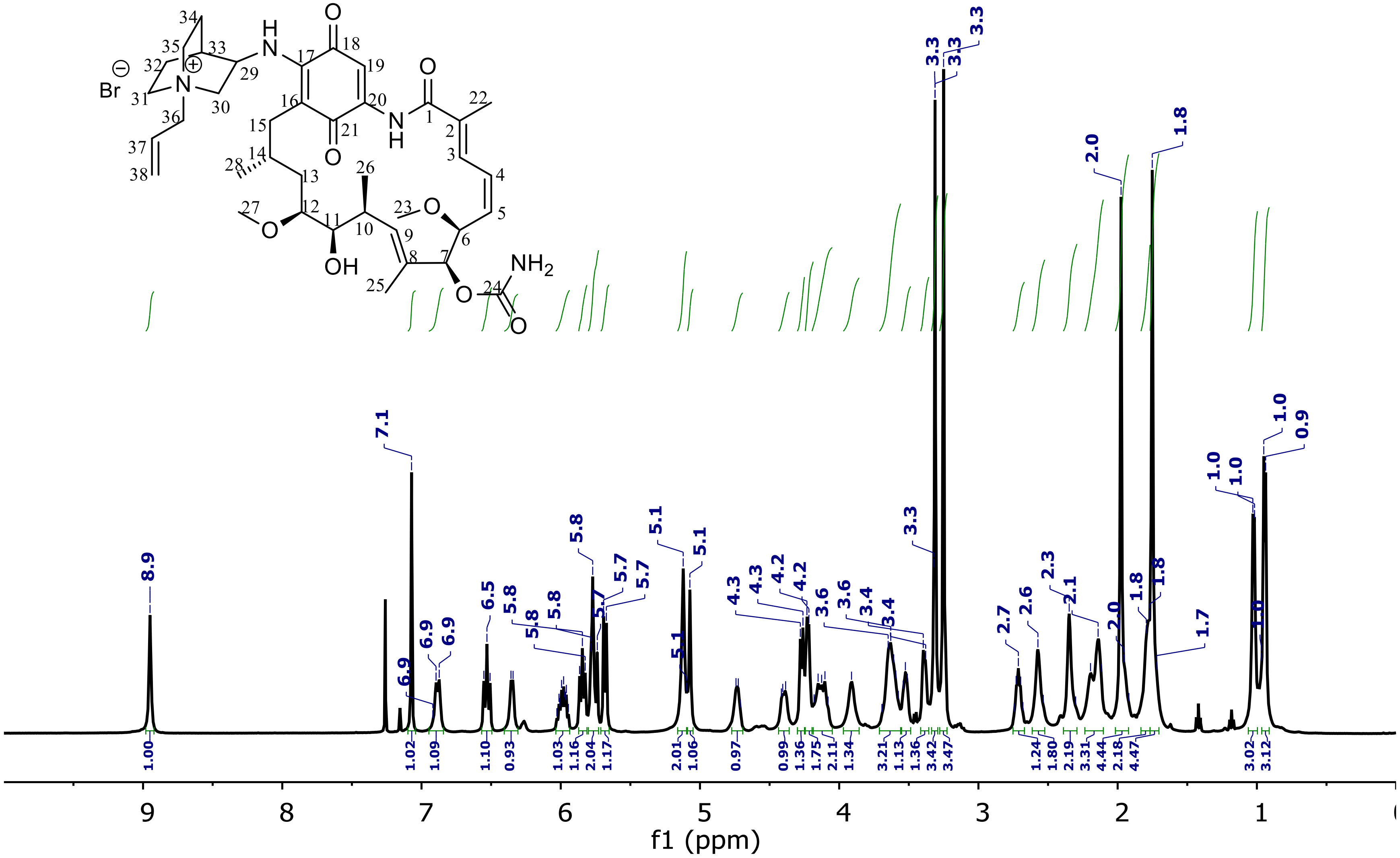

Figure 27S. ^{1}H NMR spectrum of compound **9** in $CDCl_3$.

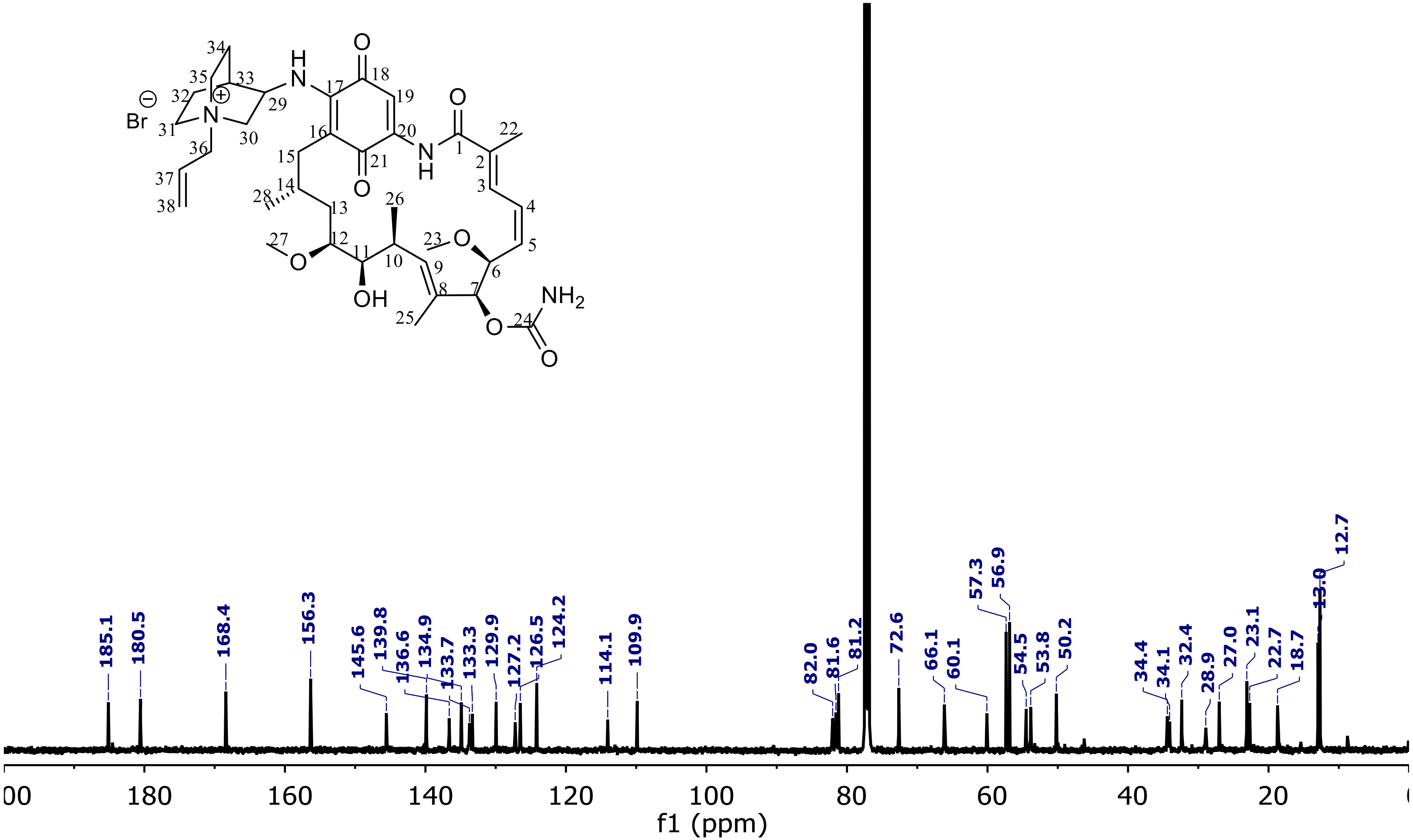

Figure 28S. ^{13}C NMR spectrum of compound **9** in $CDCl_3$.

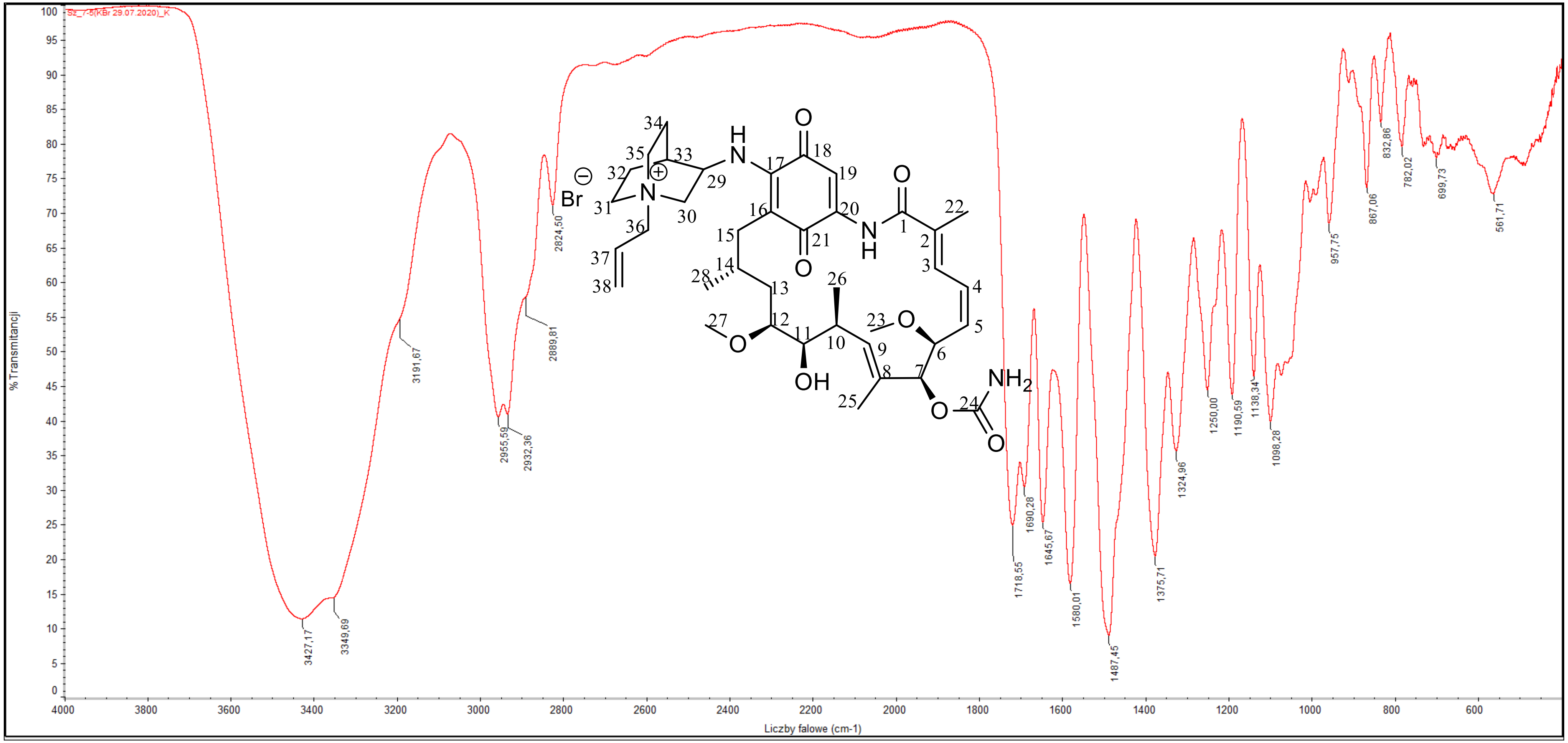

Figure 29S. FT-IR spectrum of compound **9** (in KBr).

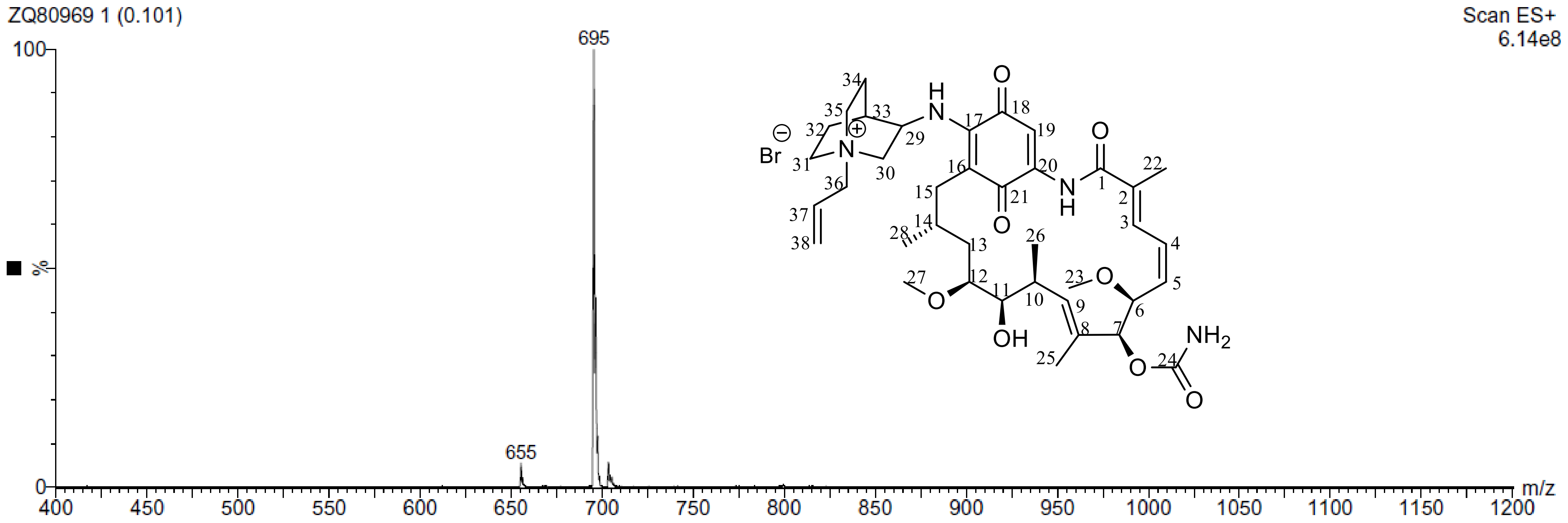

Figure 30S. ESI-MS^{+} spectrum of **9**.

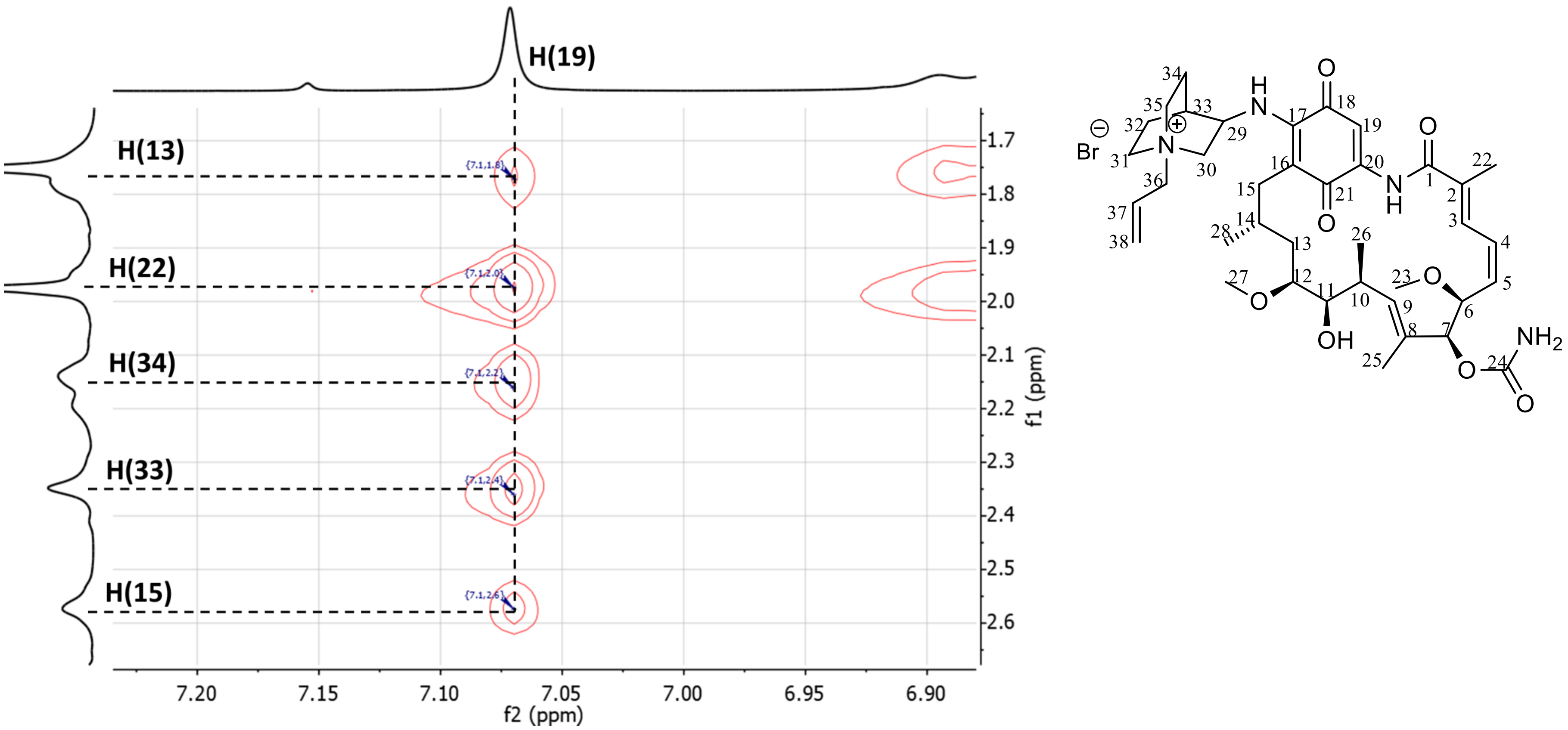

Figure 31S. ^{1}H-^{1}H NOESY contacts recorded for compound **9**.

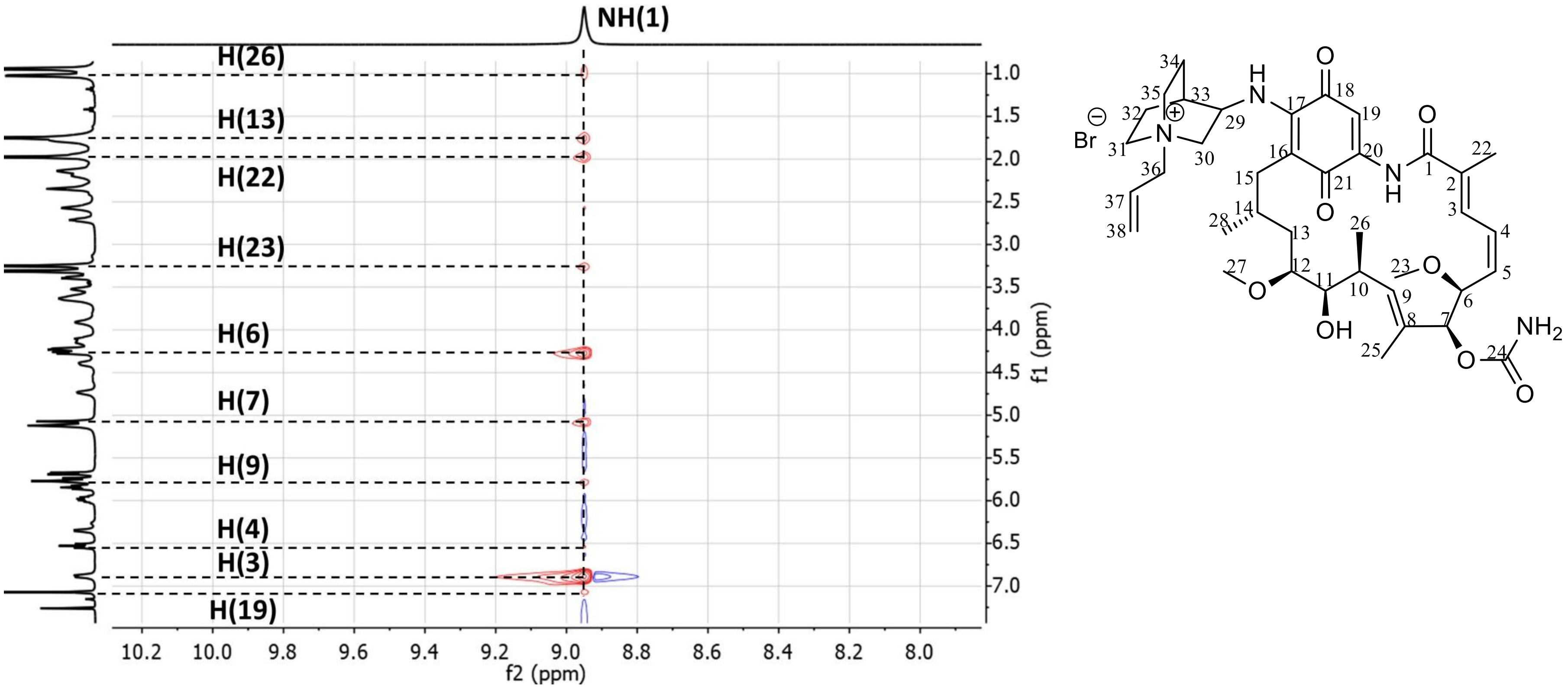

Figure 32S. ^{1}H-^{1}H NOESY contacts recorded for compound **9**.

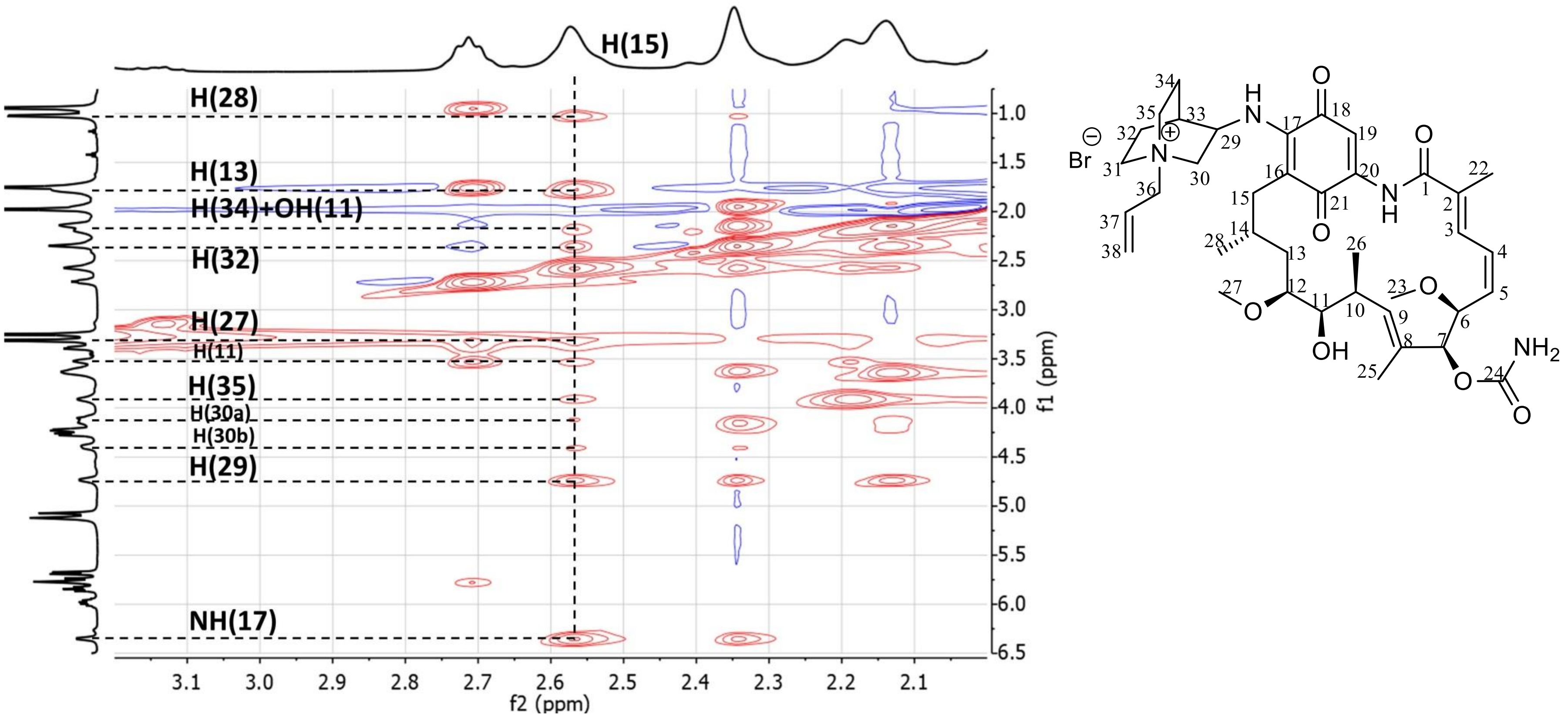

Figure 33S. ^{1}H-^{1}H NOESY contacts recorded for compound **9**.

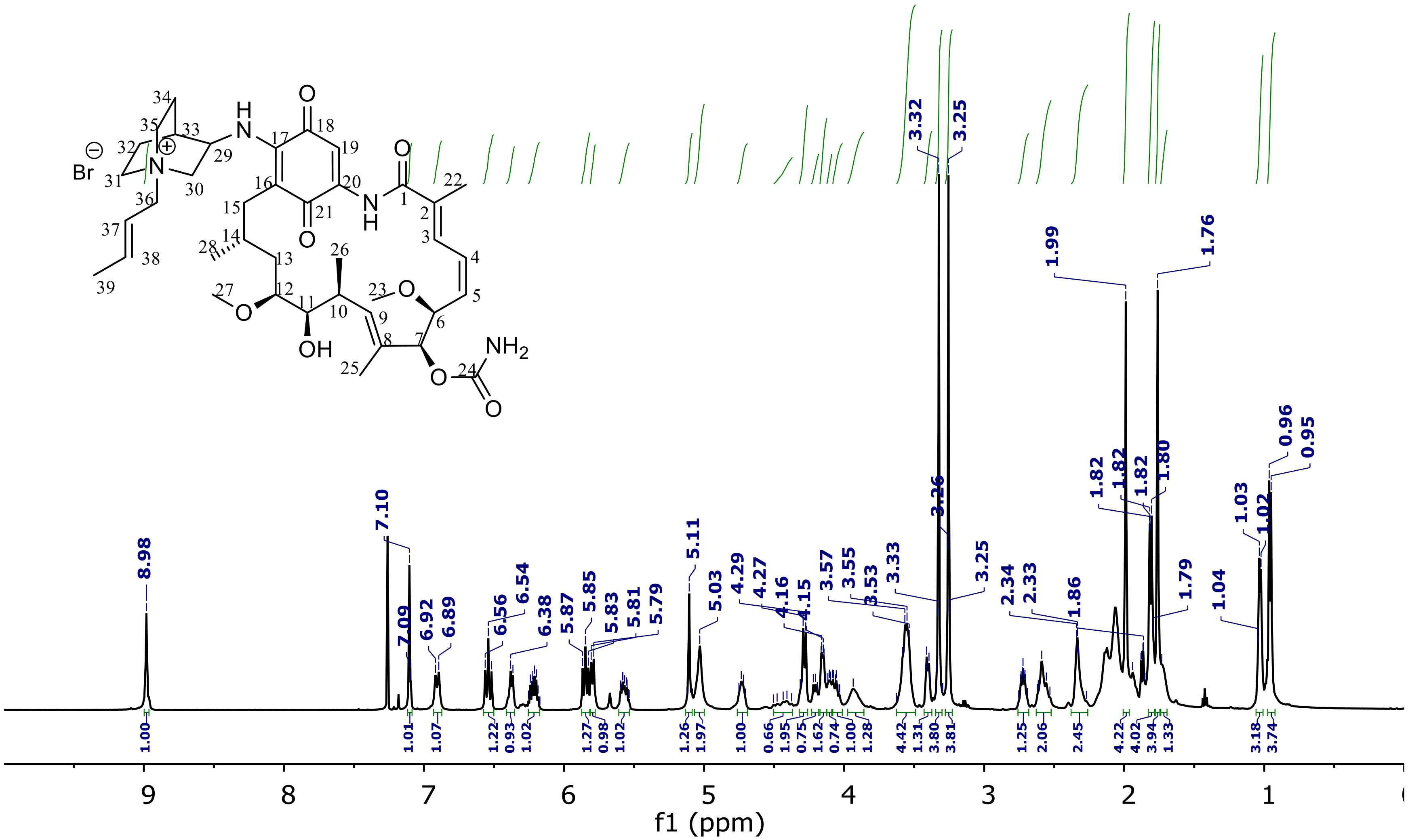

Figure 34S. ^{1}H NMR spectrum of compound **10** in $CDCl_3$.

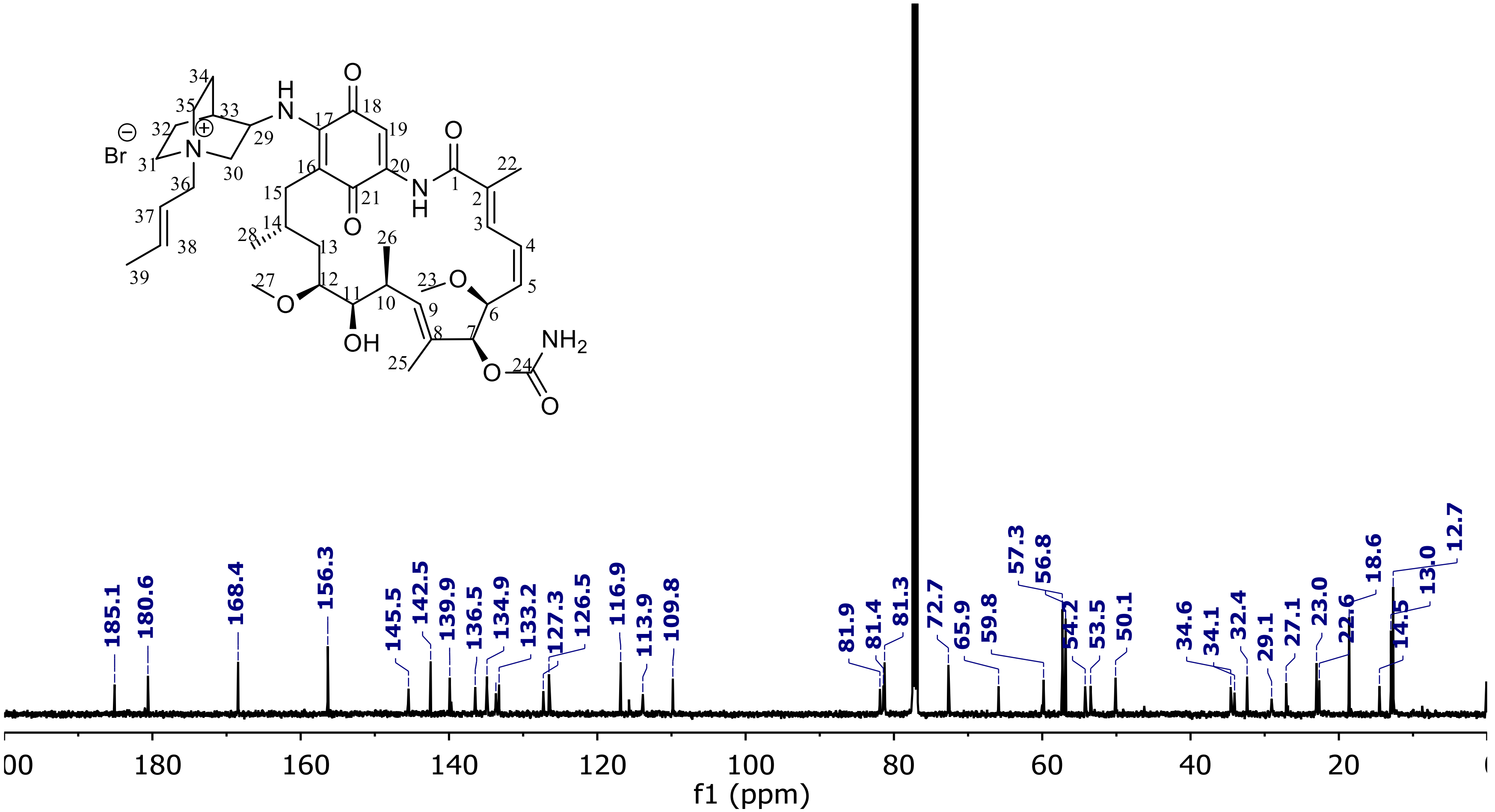

Figure 35S. ^{13}C NMR spectrum of compound **10** in $CDCl_3$.

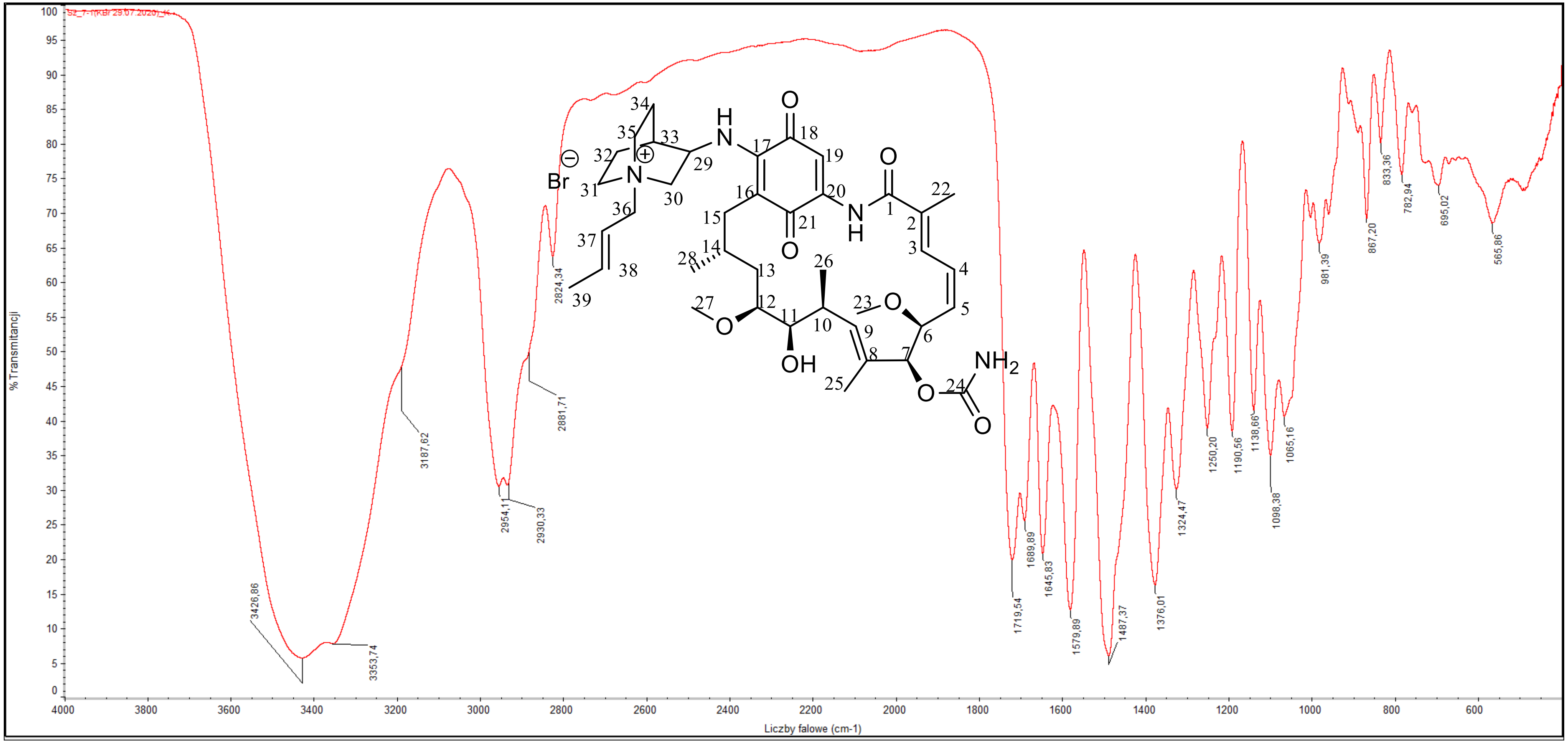

Figure 36S. FT-IR spectrum of compound **10** (in KBr).

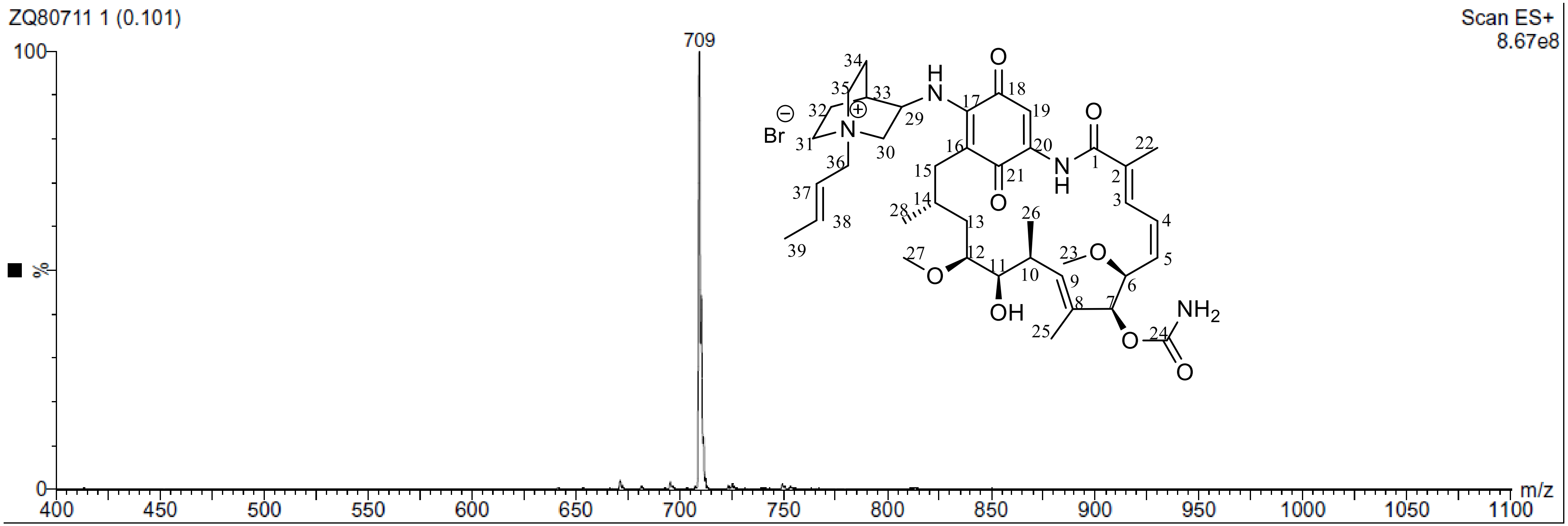

Figure 37S. ESI-MS+ spectrum of **10**.

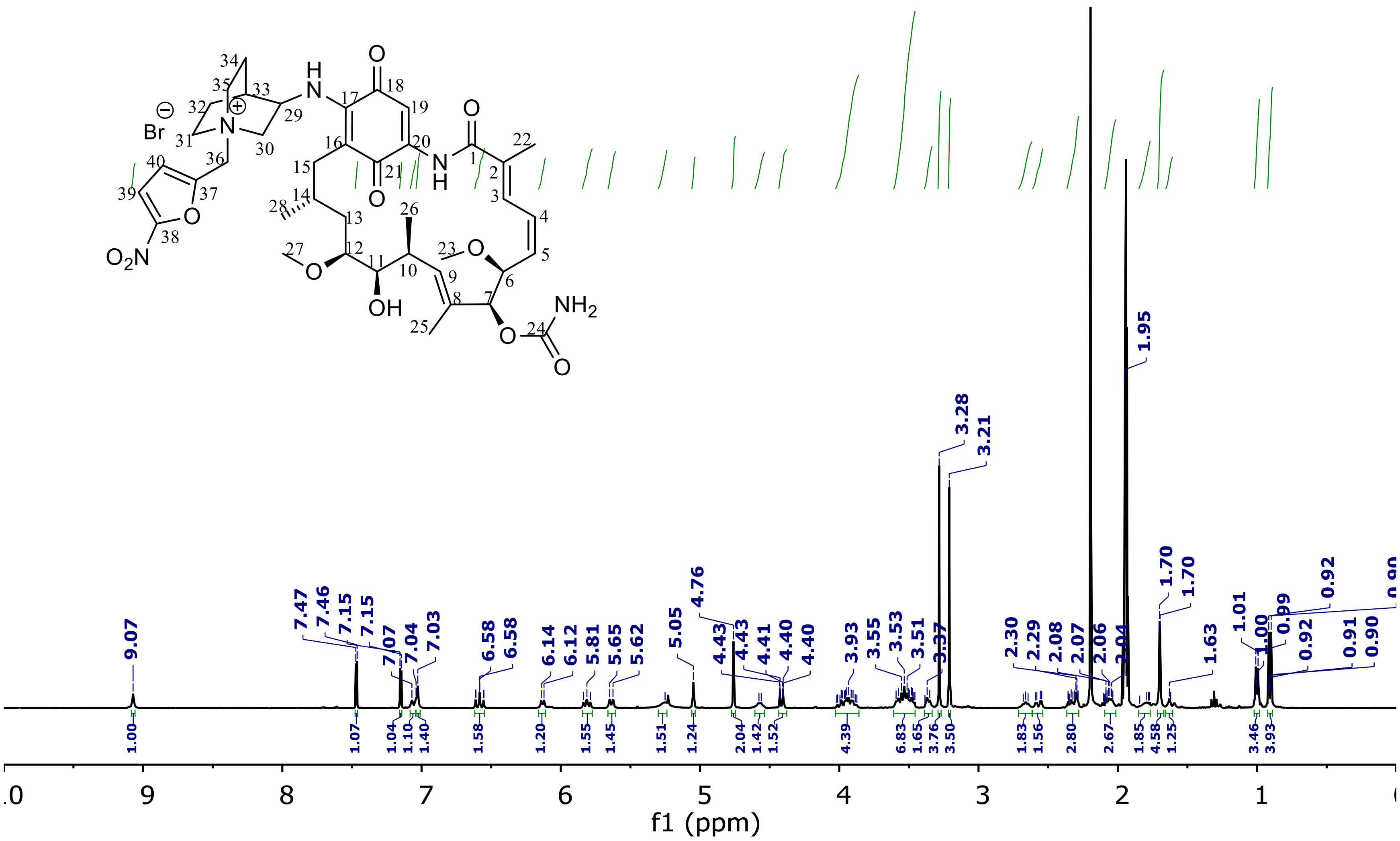

Figure 38S. ^{1}H NMR spectrum of compound **11** in CD_3CN.

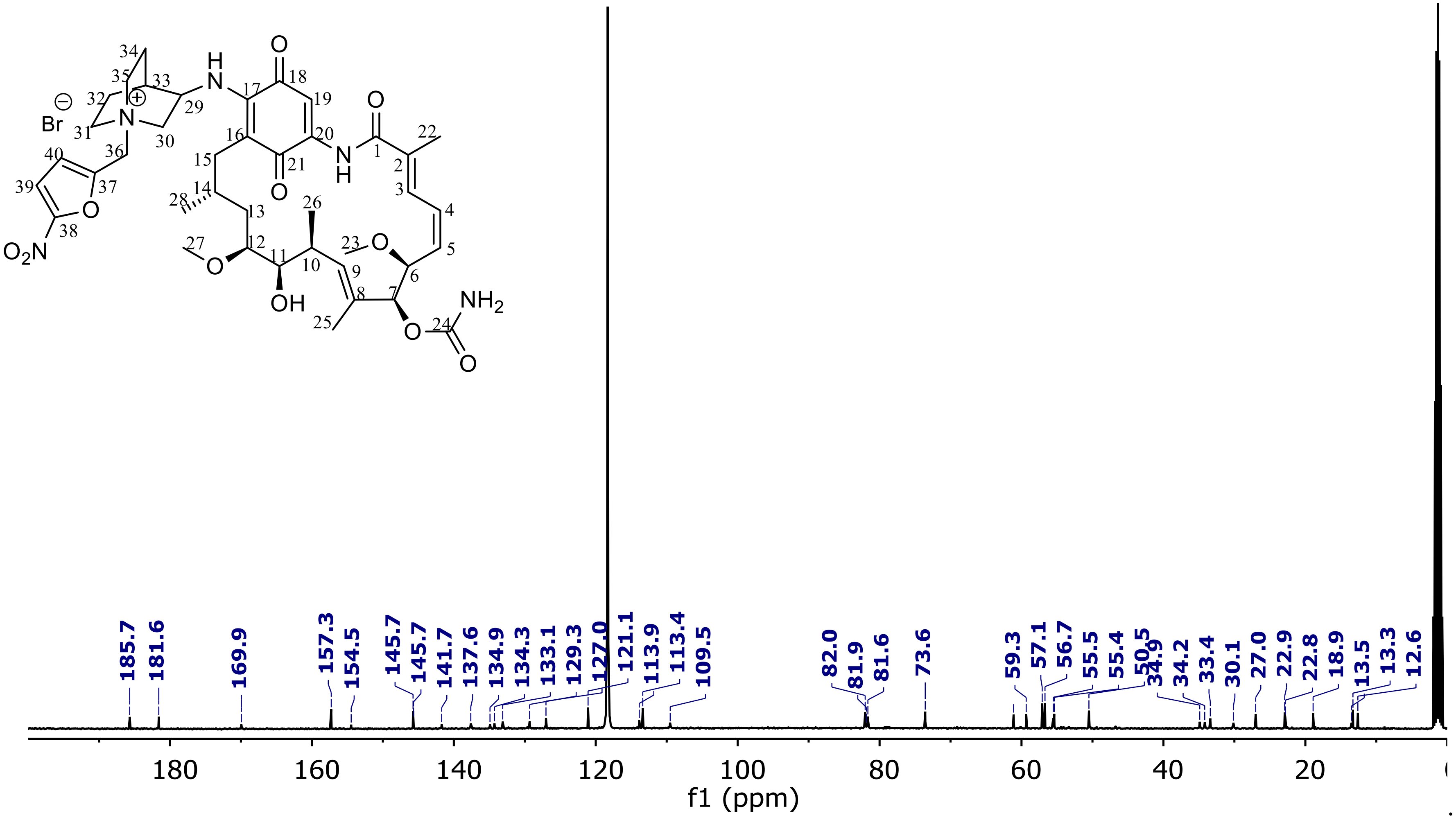

Figure 39S. ^{13}C NMR spectrum of compound **11** in CD_3CN.

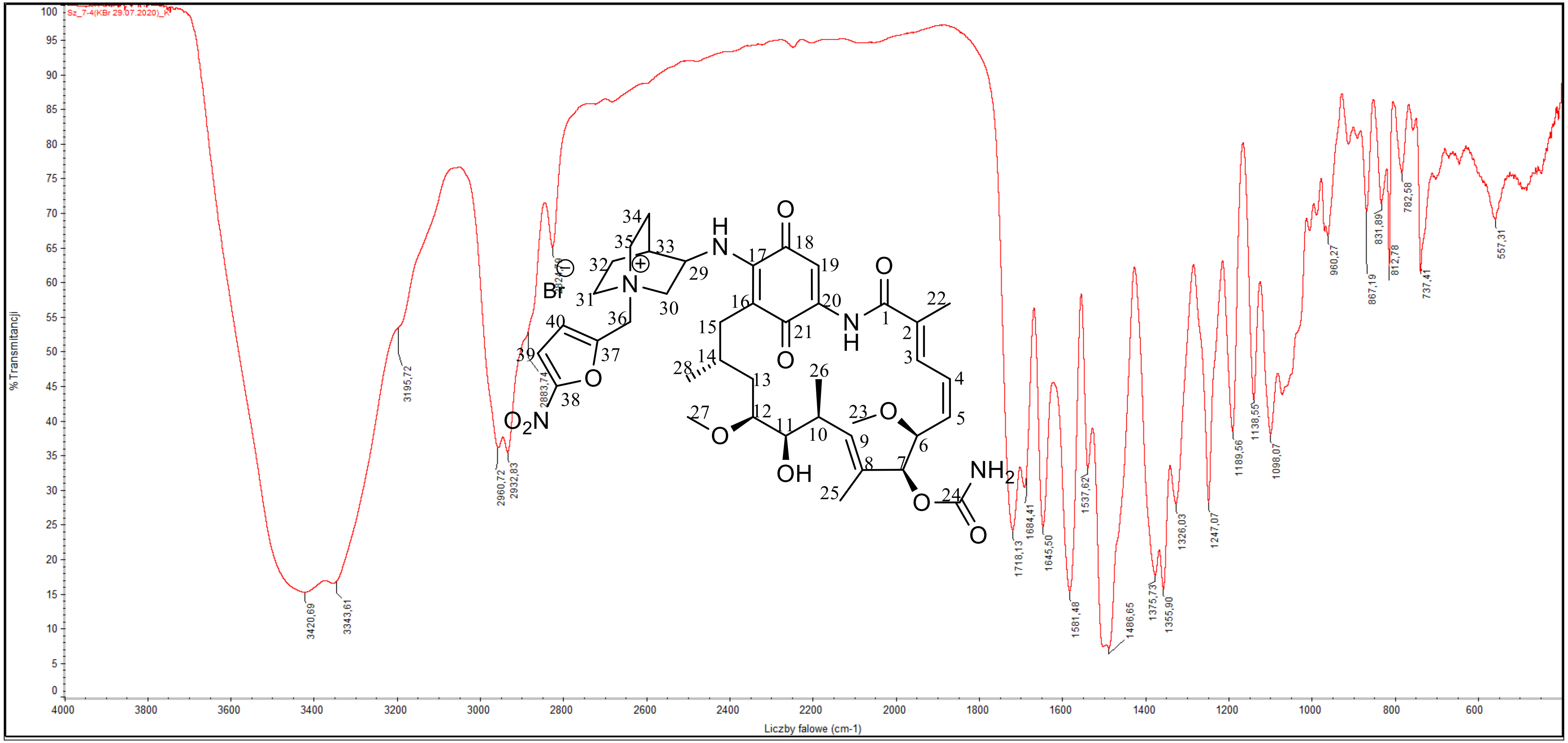

Figure 40S. FT-IR spectrum of compound **11** (in KBr).

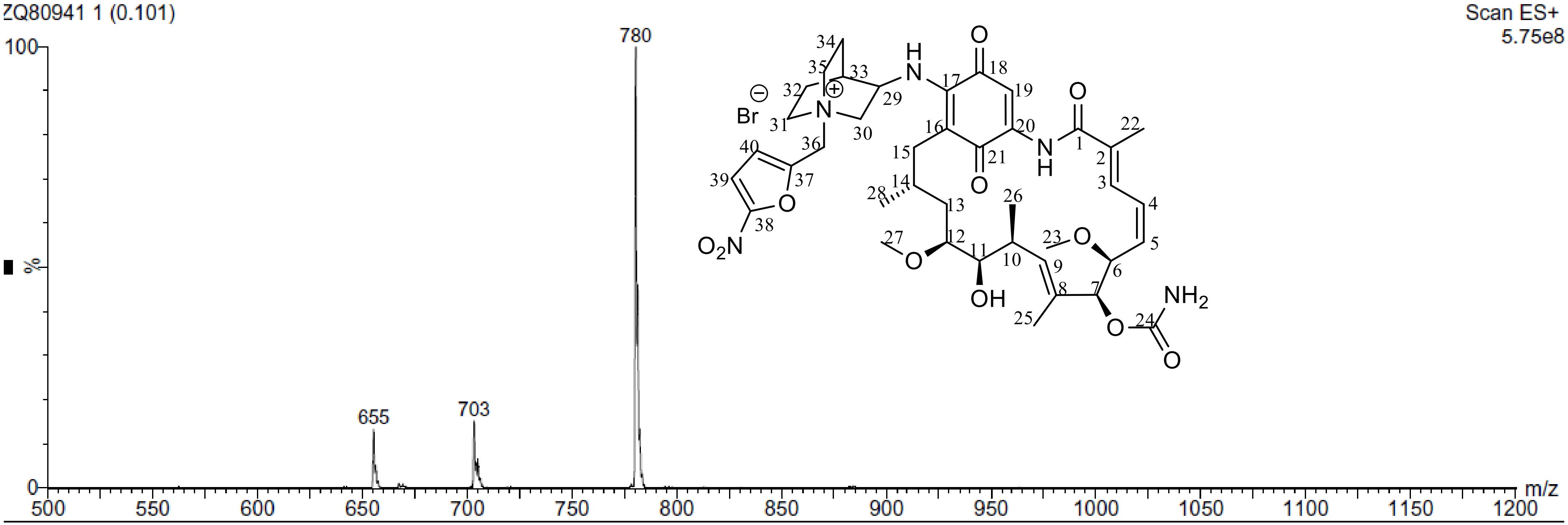

Figure 41S. ESI-MS[+] spectrum of **11**.

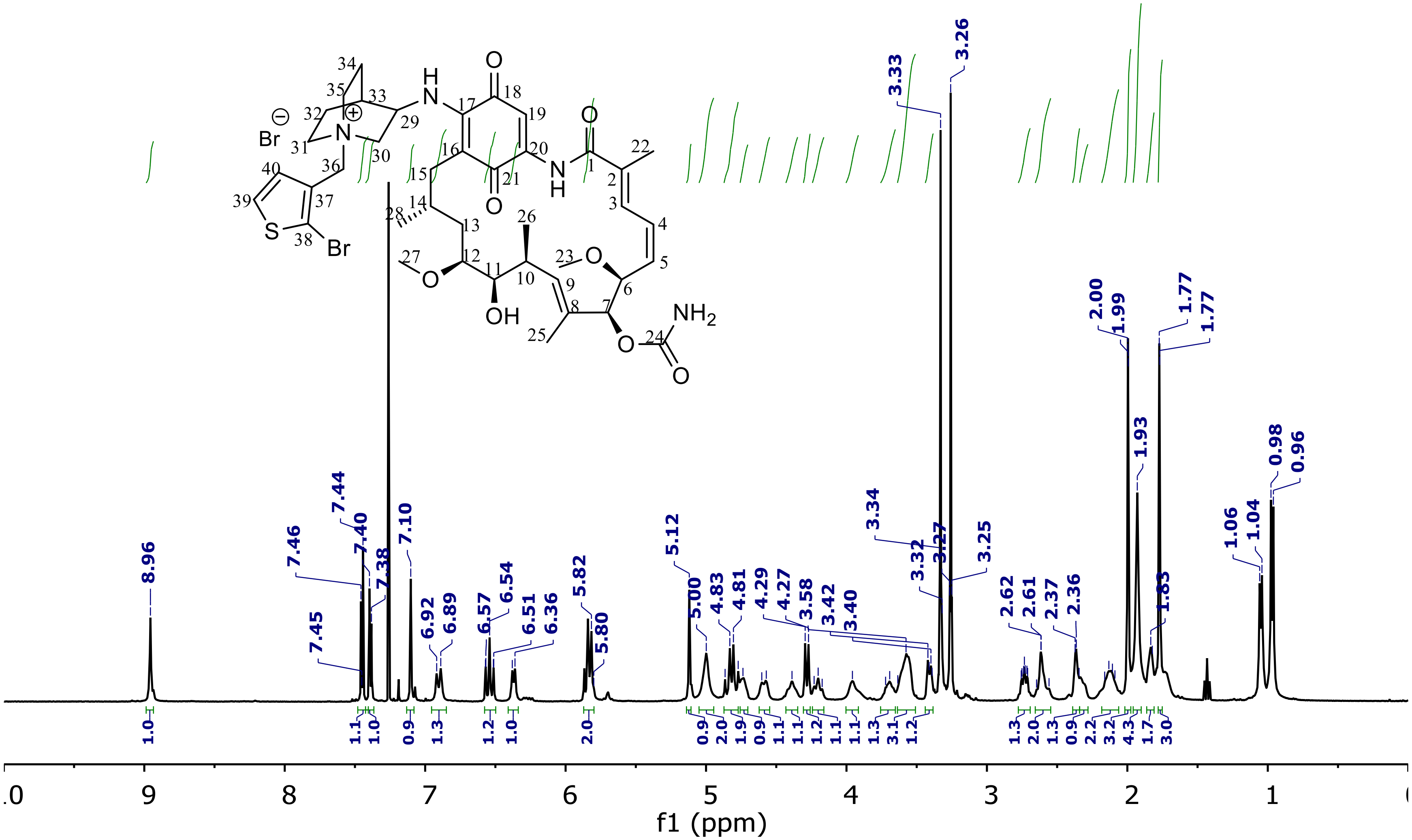

Figure 42S. ^{1}H NMR spectrum of compound **12** in $CDCl_3$.

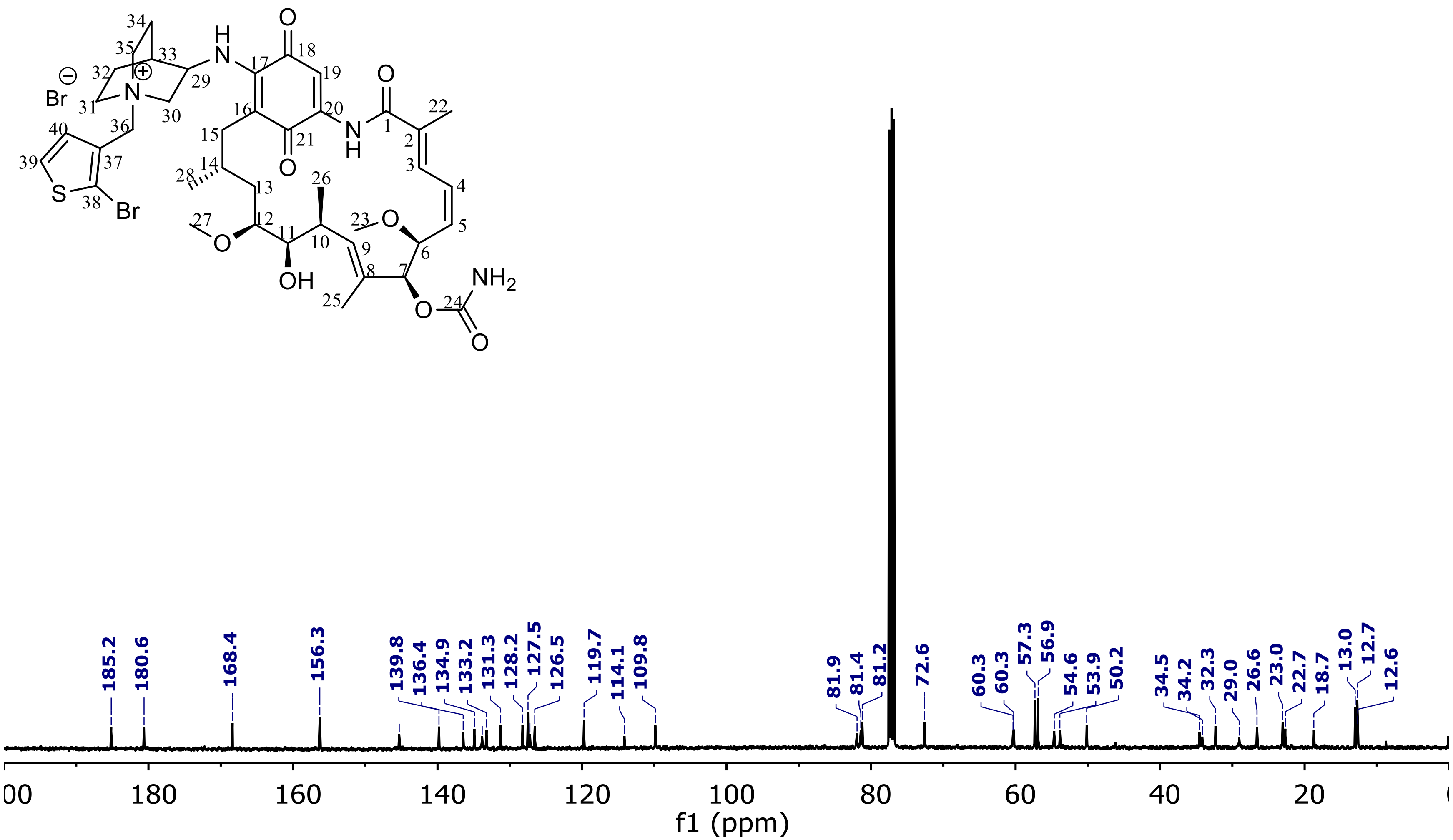

Figure 43S. ^{13}C NMR spectrum of compound **12** in $CDCl_3$.

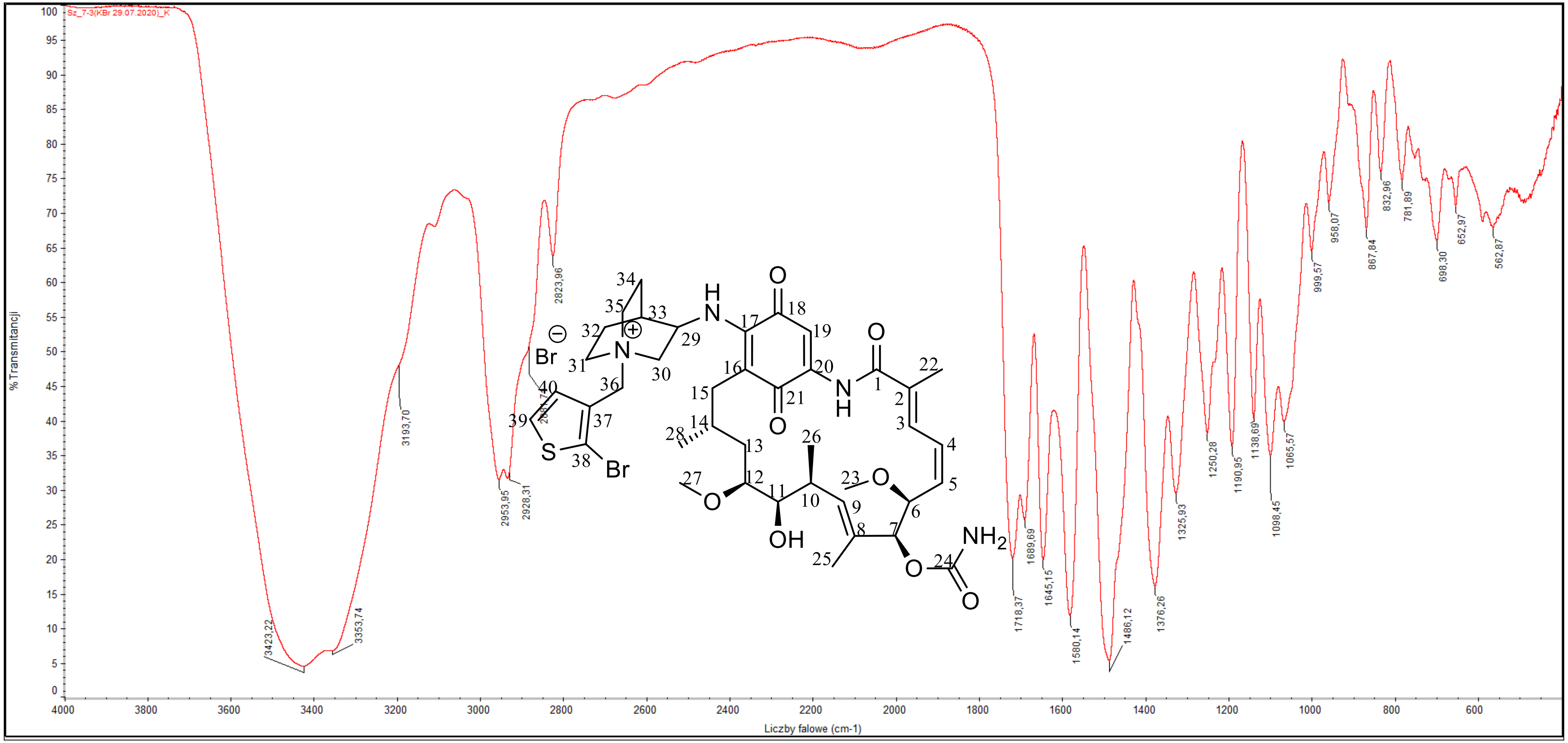

Figure 44S. FT-IR spectrum of compound **12** (in KBr).

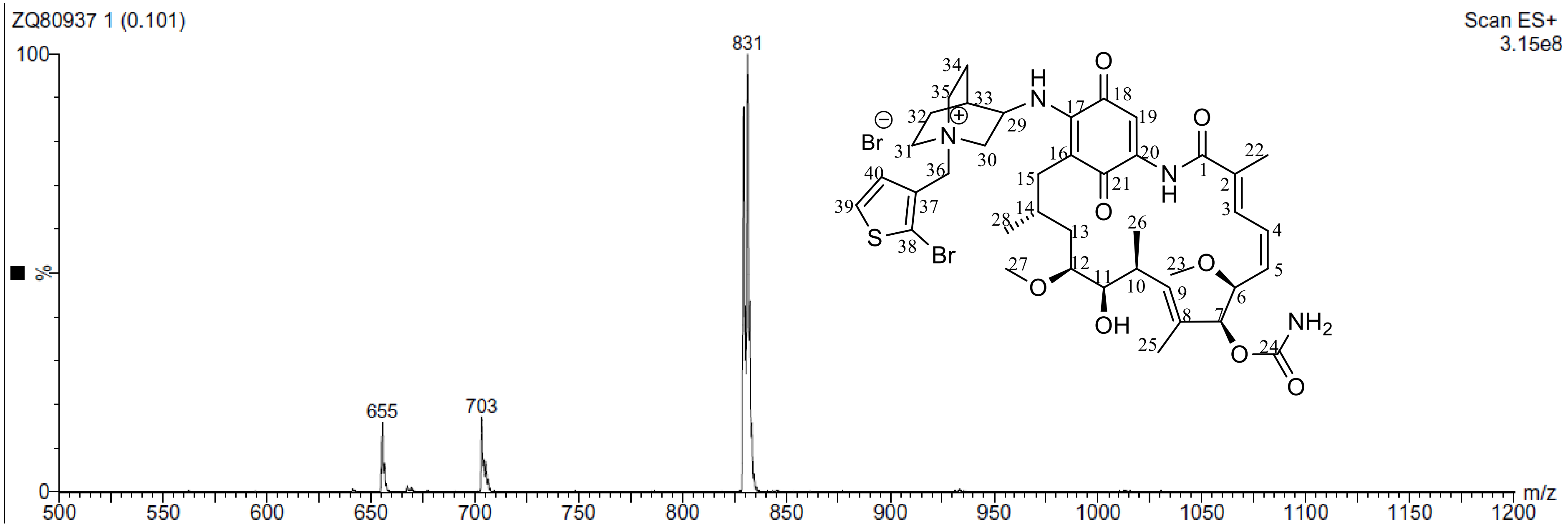

Figure 45S. ESI-MS+ spectrum of **12**.

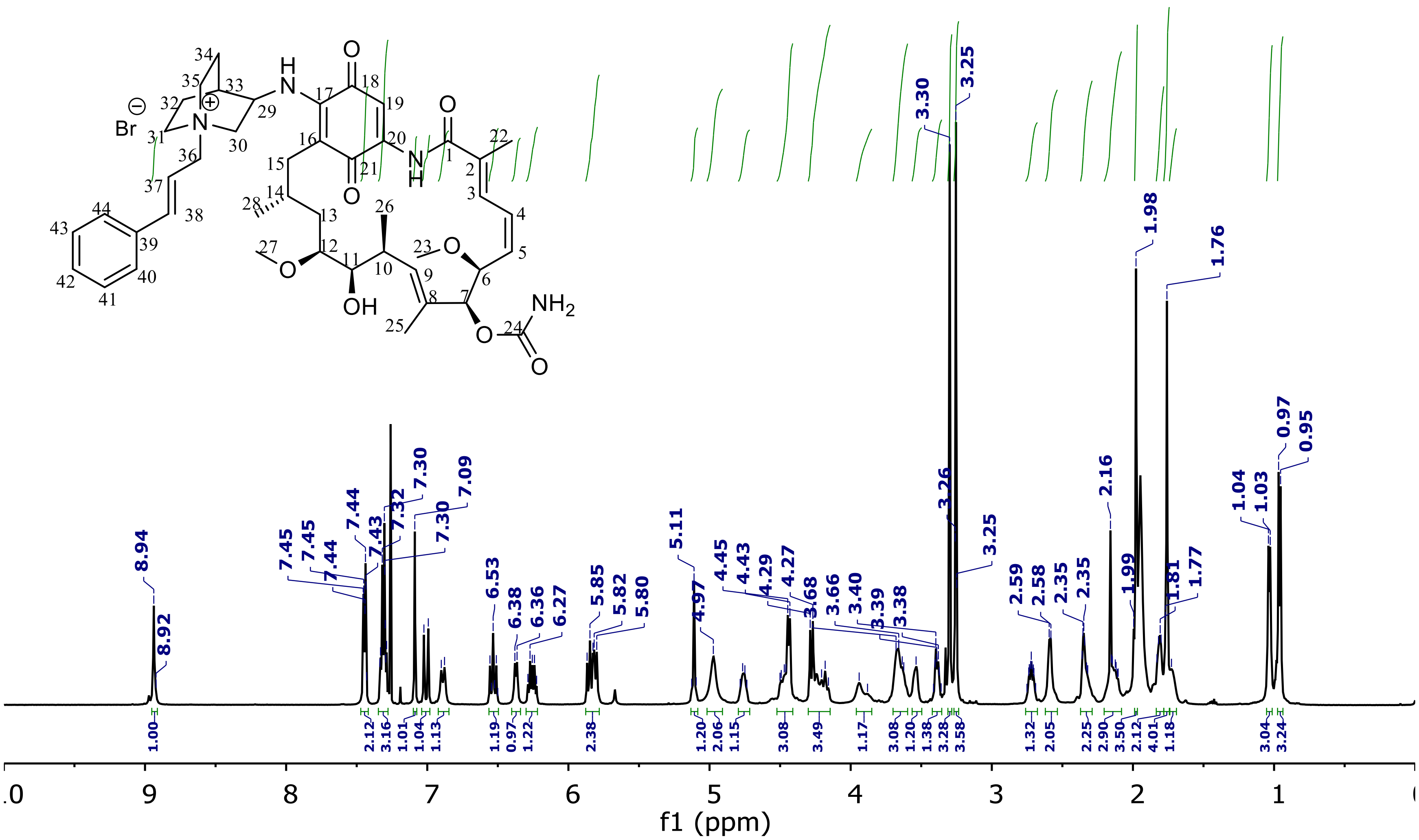

Figure 46S. ^{1}H NMR spectrum of compound **13** in $CDCl_3$.

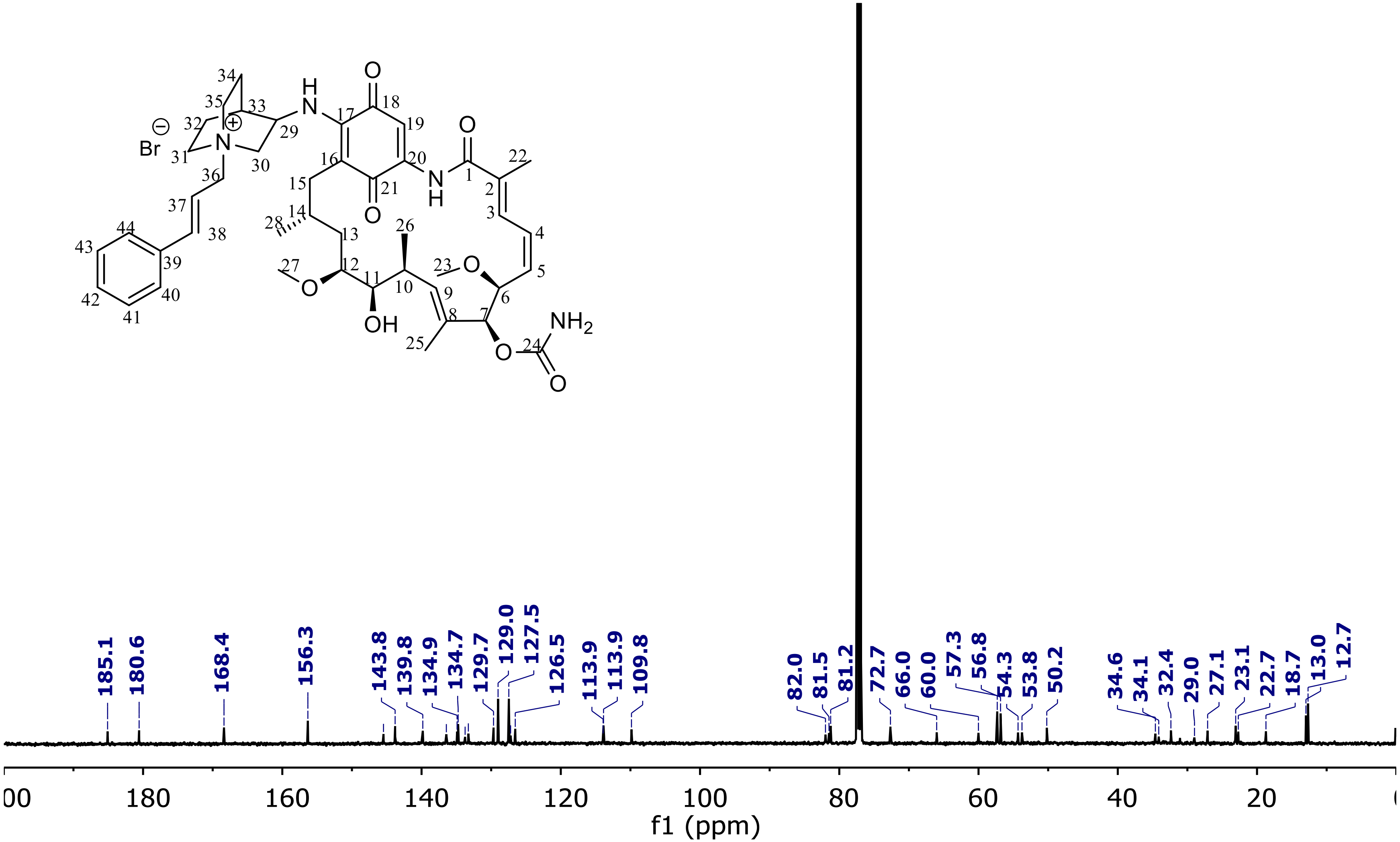

Figure 47S. ^{13}C NMR spectrum of compound **13** in $CDCl_3$.

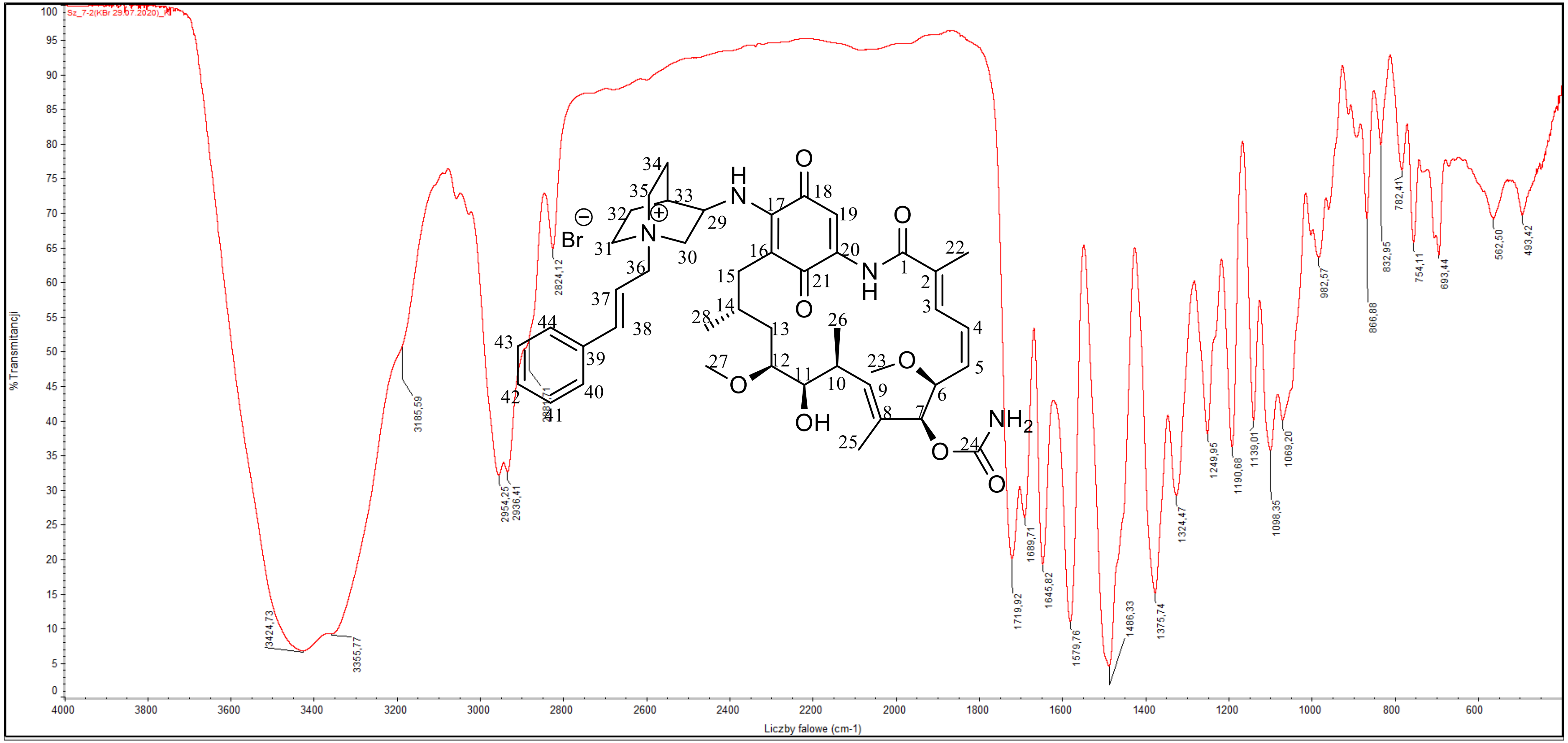

Figure 48S. FT-IR spectrum of compound **13** (in KBr).

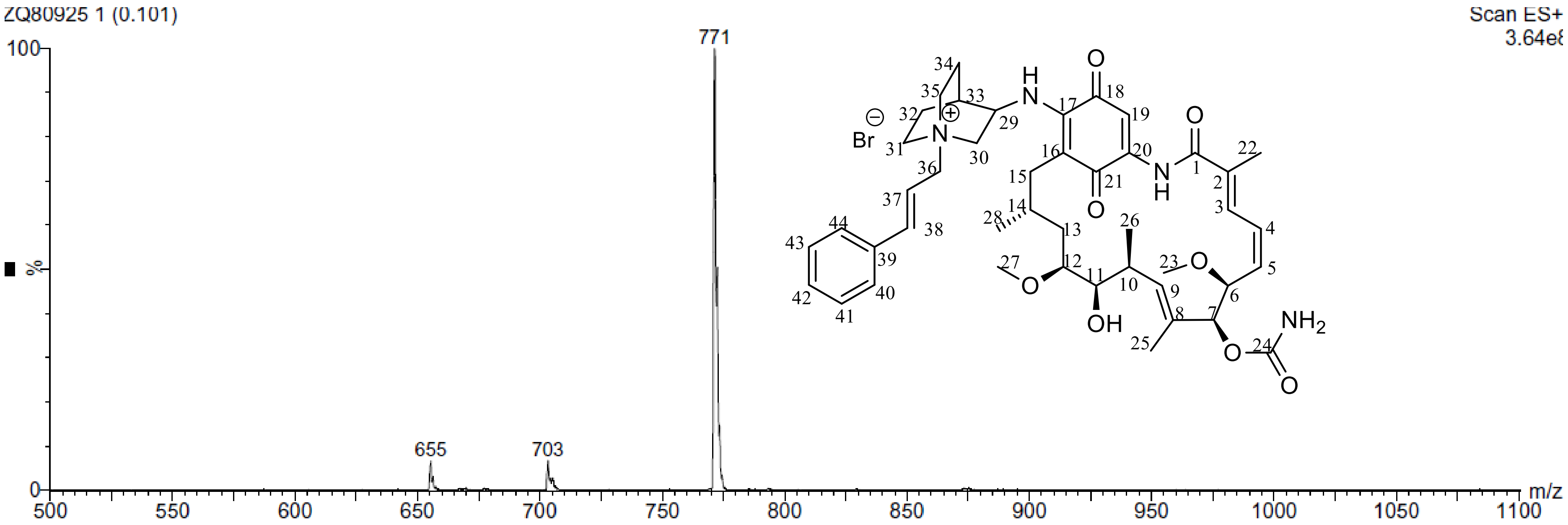

Figure 49S. ESI-MS$^+$ spectrum of **13**.

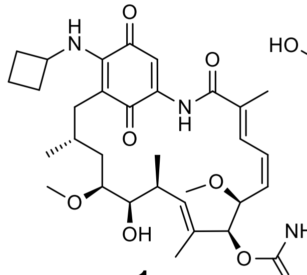

1

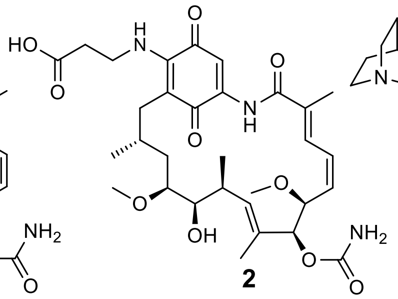

2

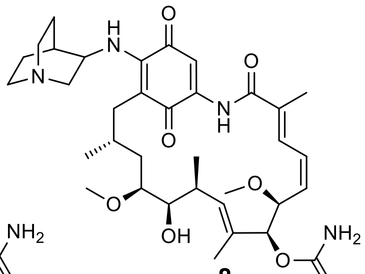

8

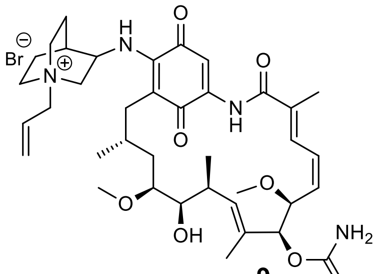

9

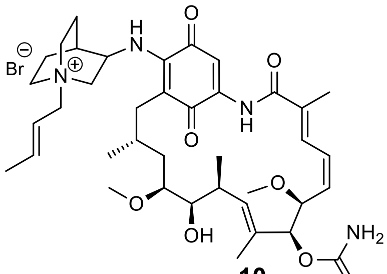

10

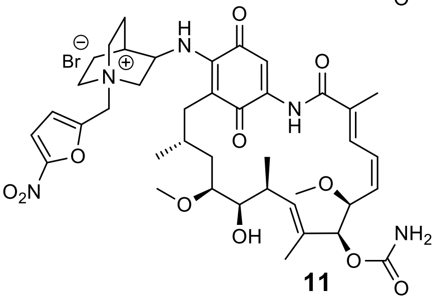

11

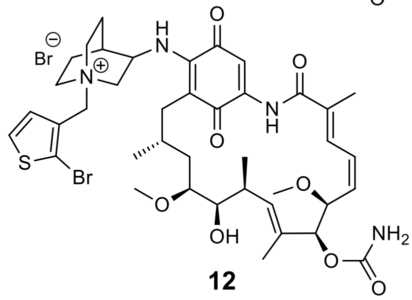

12

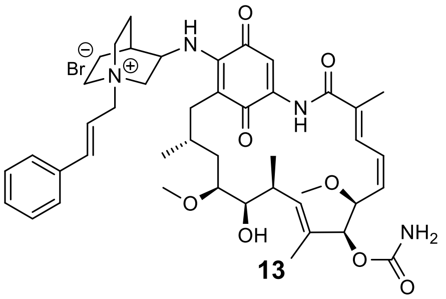

13

Figure 50S. Structures of **1,2,8-13** derivatives presented in ChemDraw Ultra 12.0.

2a

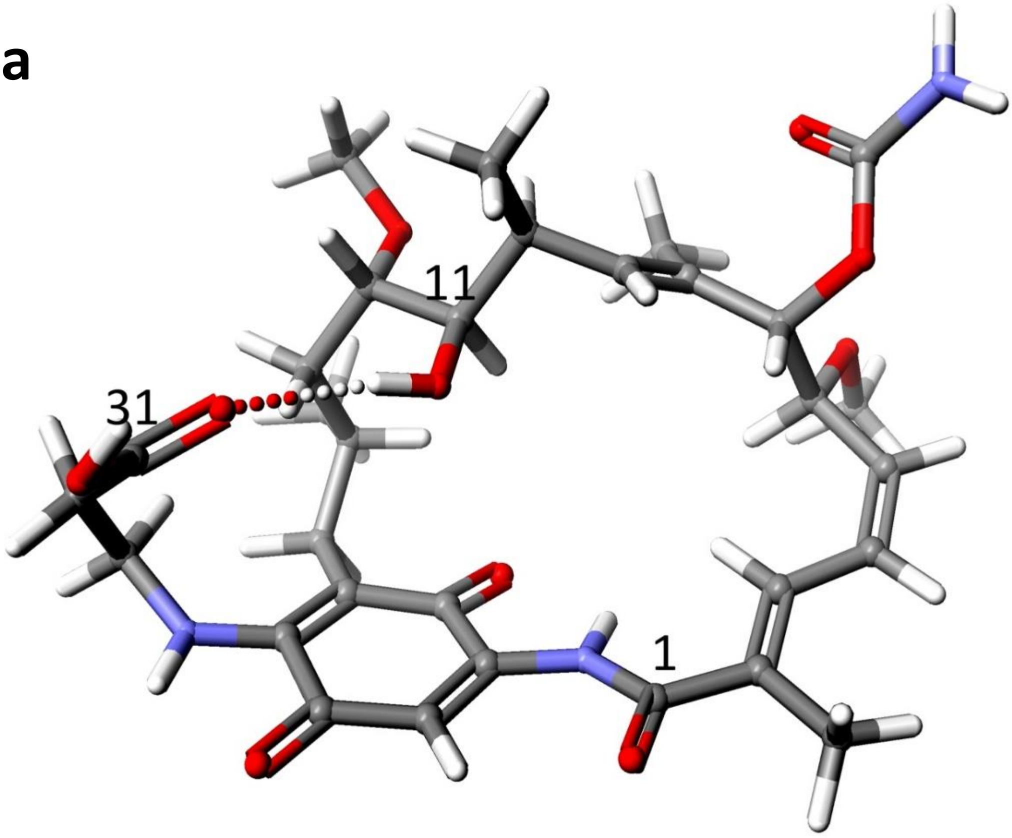

2b

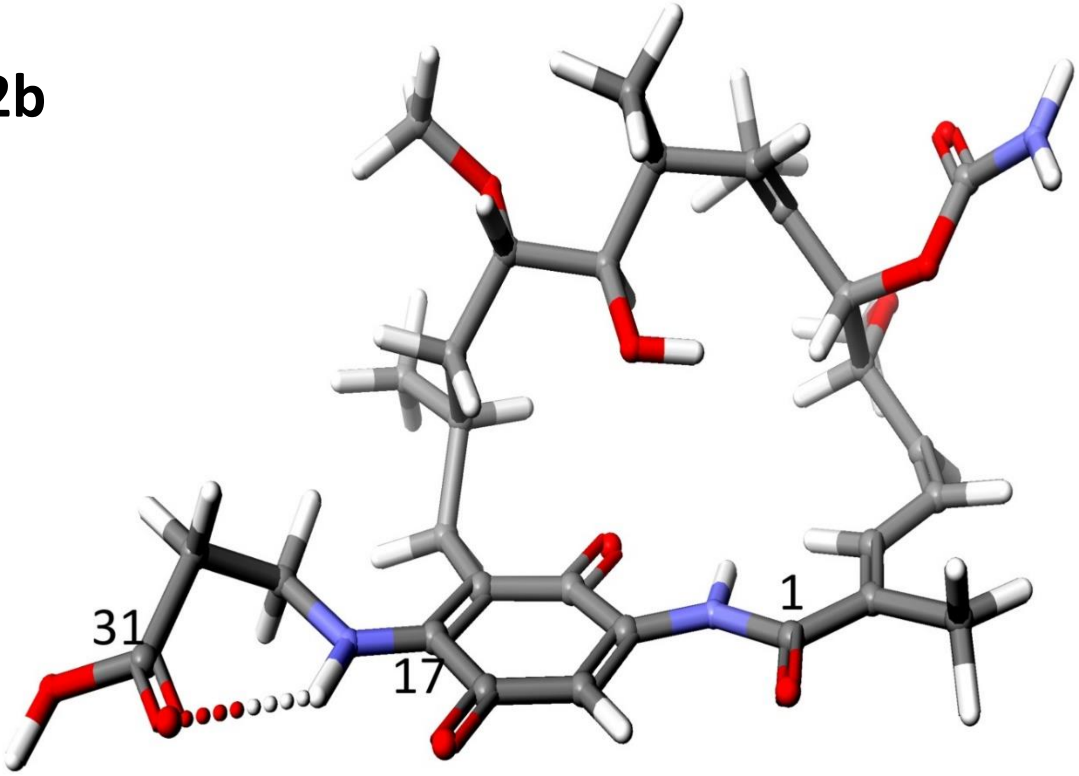

Figure 51S. Two possibilities of an alternative intramolecular H-bonding within structure of derivative **2** together with xyz coordinates (see below**),** visualized by DFT B88-LYP method. [1]

XYZ coordinates of **2a**

O 49.0484365 -3.0295715 19.0877715

O 42.8033217 -5.6403854 14.9040667

O 41.7883586 -4.426928 17.2519763

O 40.3967239 -6.4322931 17.2187024

O 45.994735 -7.4181342 20.8163934

H 46.3263023 -7.7208736 21.5976738

O 44.3050103 -10.5472664 19.7341196

N 50.5307351 -8.9719599 21.6273806

O 51.3197667 -6.4976384 21.7846883

O 48.0582277 -7.620662 17.6601787

N 48.3882081 -5.080563 18.2264939

H 47.8579249 -5.5820878 17.5018781

N 39.1833828 -4.3064024 17.451784

C 48.3423173 -3.675047 18.2910165

C 47.3921663 -2.9784039 17.3514703

C 46.341168 -3.6402957 16.7677355

H 46.215586 -4.705099 16.9911694

C 45.3033731 -3.0643852 15.9194047

H 45.4226281 -2.0195456 15.6080503

C 44.1687926 -3.7093094 15.5200096

H 43.4343412 -3.1602386 14.9164872

C 43.7332002 -5.1285818 15.8795389

H 44.618467 -5.7967186 15.9307244

C 43.0732287 -5.1795064 17.3020259

H 43.6788954 -4.5097082 17.9280461

C 43.0706191 -6.5453285 17.987471

C 43.4270726 -6.5445694 19.2971723

H 43.6561422 -5.5837629 19.7787403

C 43.585669 -7.7695385 20.1803969

H 42.9777916 -8.5916009 19.7732029

C 45.0661092 -8.2822767 20.1090902

H 45.3902921 -8.2113418 19.0614122

C 45.2188674 -9.7710483 20.559182

H 44.8915139 -9.8443689 21.6181528

C 46.6845614 -10.2735532 20.4880157

H 46.735503 -11.2739128 20.9608224

H 47.2439007 -9.5865309 21.1415164

C 47.3426847 -10.3164181 19.0733179

H 46.8474963 -9.5788149 18.4250985

C 48.8598433 -9.920963 19.0965662

H 49.4274146 -10.667263 19.6758115

H 49.2196949 -10.0083722 18.0547148

C 49.2058688 -8.5033685 19.5593679

C 50.005388 -8.1665209 20.6508302

C 50.4826761 -6.7158937 20.8750182

C 49.9253007 -5.6516655 20.0678621

H 50.2099497 -4.6246083 20.2967023

C 49.0503285 -5.9639589 19.0587224

C 48.7324949 -7.4218105 18.7084295

C 47.6259382 -1.4787942 17.259137

H 47.0860844 -1.0183869 16.4189148

H 48.6994506 -1.250163 17.1608642

H 47.2926732 -0.9944118 18.1952563

C 43.4261207 -5.9283338 13.6342741

H 42.6354724 -6.334853 12.9849851

H 44.2364287 -6.678241 13.7400139

H 43.8488295 -5.0163499 13.1682835

C 40.4588704 -5.1196509 17.3029283

C 42.7813742 -7.8287054 17.2051836

H 43.6664902 -8.4913671 17.1689535

H 42.4616532 -7.6013187 16.1812296

H 41.9665334 -8.3937705 17.6840307

C 43.1050558 -7.4837926 21.6292607

H 43.6400344 -6.6294922 22.0825473

H 43.2378341 -8.3608582 22.2882684

H 42.0314681 -7.2308969 21.631989

C 43.6747147 -11.6479956 20.4133739

H 42.9024551 -12.0368908 19.7291975

H 43.1835707 -11.3206207 21.3533438

H 44.3875637 -12.4636803 20.6532604

C 47.1954634 -11.6996017 18.3893452

H 47.7366389 -12.4881302 18.9479678

H 47.6112013 -11.6737505 17.365775

H 46.1362178 -11.9966134 18.3150423

H 38.2793247 -4.7774423 17.4864326

H 39.2315214 -3.2896299 17.5170254

C 50.040364 -10.2435895 22.182673

C 49.4165917 -9.8421411 23.6644727

C 48.5494869 -8.6445867 24.0952775

H 49.2676544 -10.6699288 21.5429429

H 48.8216667 -10.7116994 23.9438157

O 48.9021591 -7.8688372 25.3297146

O 47.4984498 -8.3016023 23.3799675

H 51.1682421 -8.4269667 22.2271083

H 50.2924693 -9.7785277 24.3101443

H 50.857593 -10.956839 22.2899665

H 48.1043419 -7.3287126 25.5551794

XYZ coordinates of **2b**

O 48.252052 -3.4564373 19.2218474

O 42.8169857 -6.4883943 14.8162288

O 42.592777 -4.7145935 17.1747417

O 40.6945887 -5.6318181 16.3242725

O 45.4339892 -7.3146561 20.0239794

H 45.0955772 -6.6199412 19.4713959

O 44.3057501 -10.8374267 20.0885539

N 50.3516083 -9.1256966 21.6384225

O 50.7101061 -6.6856372 21.937441

O 47.7345215 -7.9606039 17.9100305

N 47.8674073 -5.5098194 18.3812279

H 47.2952539 -5.9606443 17.6737736

N 40.6187545 -3.6326023 17.3015695

C 47.669229 -4.1345711 18.4111362

C 46.7546265 -3.5595378 17.5760373

C 46.1080554 -4.3216834 16.6714149

H 46.3783203 -5.3799906 16.5622317

C 45.1006616 -3.9853671 15.8519357

H 44.6754556 -2.9705253 15.8349487

C 44.5508799 -4.8927838 15.0665559

H 44.5880474 -4.7653568 13.983503

C 43.882201 -6.0886307 15.6528128

H 44.6251982 -6.920673 15.699291

C 43.3353006 -5.9108554 17.0875465

H 44.2209824 -5.6979046 17.7293934

C 42.6847553 -7.0752661 17.804712

C 42.5598641 -6.9492397 19.1140384

H 42.106097 -6.0653688 19.5659053

C 43.0910996 -8.1244682 19.9174685

H 42.4738989 -9.0172221 19.6631712

C 44.5774083 -8.5027815 19.7005183

H 44.7518653 -8.719752 18.6213364

C 45.0332878 -9.7156254 20.5459848

H 44.7524307 -9.5055669 21.6026005

C 46.5463109 -10.0192877 20.5627657

H 46.780179 -10.7474795 21.3730405

H 47.0370276 -9.0756872 20.888442

C 47.1855983 -10.5310728 19.2565162

H 46.6255977 -10.0561477 18.4173818

C 48.6817942 -10.1457361 19.1159566

H 49.3580375 -10.9286921 19.510025

H 48.9469491 -10.1816632 18.032894

C 49.0158459 -8.7520885 19.6238336

C 49.7978182 -8.400476 20.7480743

C 50.0125817 -7.0272248 20.9749747

C 49.4267391 -6.0071357 20.2231018

H 49.5948707 -4.9746165 20.5028767

C 48.5988721 -6.344967 19.1546123

C 48.4601484 -7.706722 18.8779514

C 46.4965131 -2.0788348 17.7513458

H 45.7973864 -1.6406688 17.0085057

H 47.4453952 -1.5042301 17.6531024

H 46.0632679 -1.8854219 18.7589773

C 43.1956953 -7.3470768 13.7649021

H 42.2848491 -7.5157207 13.1482059

H 43.5369684 -8.3217149 14.1841419

H 43.9845338 -6.8726146 13.1384254

C 41.2944655 -4.7384741 16.8617951

C 42.1219785 -8.3276556 17.1836053

H 42.904499 -9.114596 17.0971074

H 41.7280881 -8.1272222 16.164618

H 41.2546321 -8.7198475 17.7617623

C 42.768451 -7.8247206 21.3977527

H 43.4265162 -7.0191821 21.7968625

H 42.8589842 -8.7141362 22.0598862

H 41.7134934 -7.4857447 21.5184451

C 44.0174155 -11.7853424 21.0900448

H 43.528436 -12.647125 20.5840806

H 43.3064166 -11.3413498 21.8238296

H 44.9547901 -12.1319258 21.5797444

C 47.0380395 -12.0537234 19.088746

H 47.5301538 -12.5989862 19.9266658

H 47.5055664 -12.399999 18.1382583

H 45.9720014 -12.3701372 19.0560132

H 39.6211093 -3.6478141 17.1074382

H 41.1473966 -2.9181296 17.7947772

C 50.1147025 -10.577818 21.808465

C 50.262849 -10.9989154 23.2783485

C 51.623776 -10.6416739 23.8355077

H 49.0680783 -10.8269975 21.5315286

H 50.1023724 -12.0998356 23.3677498

O 52.5114081 -11.7942294 24.2011919

O 52.0293196 -9.6618854 24.0000923

H 51.0091347 -8.708917 22.3876777

H 49.4910264 -10.4785043 23.8936265

H 50.8624981 -11.1258432 21.1885643

H 53.3777368 -11.6326589 24.555973

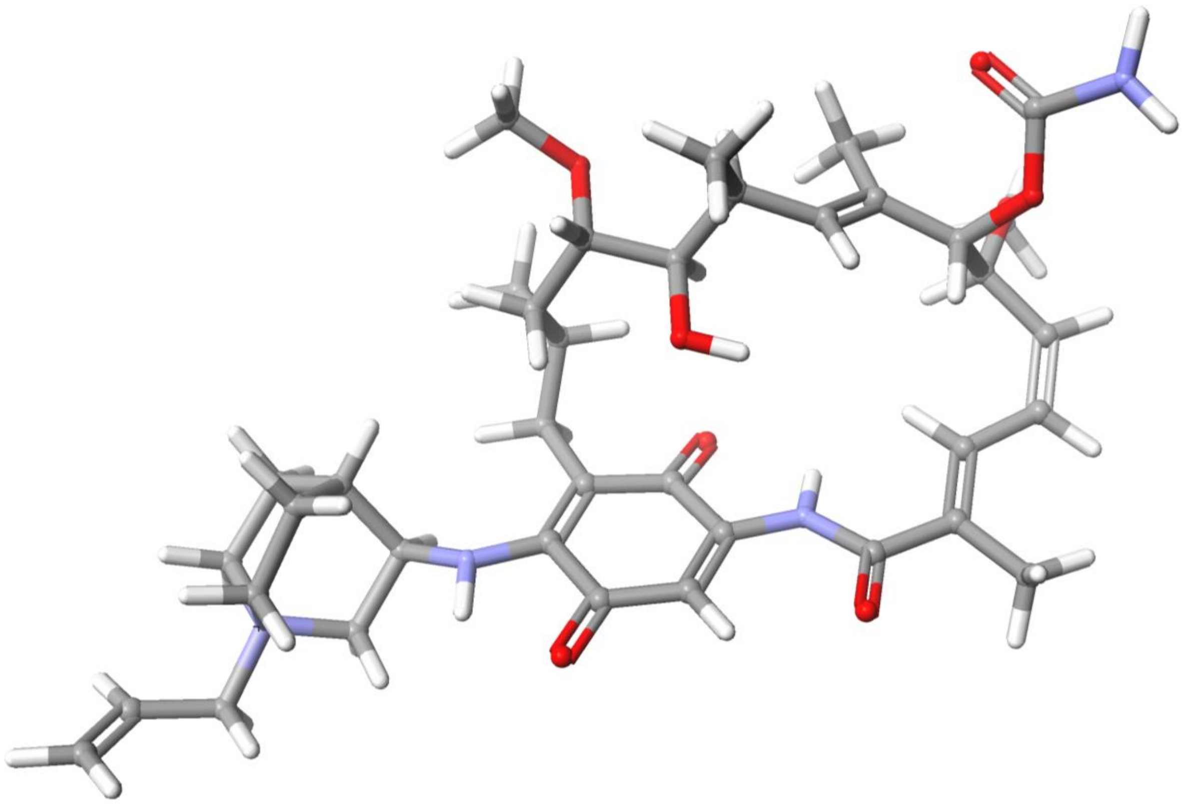

Figure 52S. Structure and xyz coordinates (see below) of derivative **9** (Fig. 2 – manuscript), calculated by DFT B88-LYP method [1]

XYZ coordinates of **9**

O 48.5759495 -3.2897712 19.3789778

O 42.5485155 -5.7649682 14.8073148

O 41.8306195 -4.2487032 17.0810518

O 40.1461235 -5.8578052 17.2727268

O 45.5403115 -7.3180642 20.8459048

H 45.4479275 -6.4887472 20.3292508

O 43.8129735 -10.5293242 20.5485198

N 50.2257685 -9.0362272 22.2138598

O 50.6548095 -6.5109292 22.4282678

O 47.8281565 -7.9746932 18.1059138

N 47.9917775 -5.3797142 18.5627428

H 47.5705405 -5.9163222 17.7940898

N 39.5981464 -3.4944715 17.0031367

C 47.9625215 -3.9600752 18.5311418

C 47.1704355 -3.3065552 17.4377308

C 46.2095595 -3.9822712 16.7245288

H 46.0245585 -5.0358372 16.9668838

C 45.3439825 -3.4290432 15.6876898

H 45.6090735 -2.4516422 15.2674748

C 44.2044305 -4.0197552 15.2262868

H 43.6226675 -3.4933662 14.4604378

C 43.5790155 -5.3294402 15.7082898

H 44.3657895 -6.1105492 15.7825218

C 42.9698685 -5.1860712 17.1505308

H 43.7002585 -4.5947312 17.7185148

C 42.7744455 -6.5002882 17.9210188

C 43.1500195 -6.4898042 19.2272288

H 43.4769755 -5.5313502 19.6627958

C 43.1378035 -7.6765902 20.1864058

H 42.5255635 -8.4818922 19.7533478

C 44.5862295 -8.2658032 20.2755288

H 44.8865475 -8.4983932 19.2374708

C 44.7321575 -9.5640692 21.1185488

H 44.4278135 -9.3353522 22.1603978

C 46.2071075 -10.0714802 21.1714278

H 46.2480025 -10.9554772 21.8388298

H 46.7742995 -9.2695022 21.6718648

C 46.8749285 -10.4197372 19.8040528

H 46.4472205 -9.7785162 19.0209738

C 48.4199245 -10.1440392 19.8023108

H 48.8822235 -10.8602302 20.4967248

H 48.7931745 -10.4011542 18.7945668

C 48.8505205 -8.7128712 20.1297778

C 49.6217365 -8.2998352 21.2024428

C 49.9110175 -6.8031012 21.4546268

C 49.3393325 -5.8014552 20.5869748

H 49.5208815 -4.7505762 20.8114158

C 48.6013105 -6.1954222 19.4945338

C 48.3946155 -7.6798752 19.1856218

C 47.4444445 -1.8169932 17.3131098

H 46.9850255 -1.3762612 16.4180008

H 48.5281105 -1.6218422 17.2912538

H 47.0502245 -1.2874342 18.1989628

C 43.0595155 -6.3514232 13.5892818

H 42.1809655 -6.6265172 12.9850488

H 43.6558435 -7.2624262 13.8003158

H 43.6873095 -5.6365972 13.0205258

C 40.5216035 -4.6935332 17.1441258

C 42.2781435 -7.7620392 17.2135538

H 43.0904235 -8.5071112 17.1096808

H 41.8812715 -7.5234572 16.2204468

H 41.4663655 -8.2352682 17.7881428

C 42.5310795 -7.2734462 21.5568238

H 43.1686045 -6.5450432 22.0864178

H 42.3960315 -8.1498732 22.2131998

H 41.5386955 -6.8152892 21.4106868

C 43.3747845 -11.5455532 21.4703378

H 42.6632085 -12.1797062 20.9181628

H 42.8598515 -11.1052742 22.3483118

H 44.2097945 -12.1811752 21.8283988

C 46.6350475 -11.8931132 19.3888578

H 47.0951735 -12.5938872 20.1134008

H 47.0706365 -12.1046422 18.3961868

H 45.5584555 -12.1187212 19.3407168

H 38.5866931 -3.6242996 17.0256496

H 39.989904 -2.5600154 16.8860133

C 50.7233995 -10.4133312 22.2107788

C 52.2101375 -10.4588282 22.7232458

N 52.3383615 -11.3013092 24.0088048

C 49.8815485 -11.3720802 23.1113528

C 49.8991145 -10.8544532 24.5711058

C 51.3716555 -10.7300122 25.0680248

C 50.5134655 -12.7859242 23.0510078

C 51.9326695 -12.7538592 23.6924668

H 50.8355217 -10.7422295 21.1634941

H 52.8814925 -10.9125532 21.9789148

H 52.5895635 -9.4536492 22.9609808

H 48.8465515 -11.4029642 22.7401128

H 49.3346835 -11.5389192 25.2242558

H 49.4091535 -9.8709162 24.6241138

H 51.5598145 -11.2849012 25.9970578

H 51.6730505 -9.6822652 25.2239478

H 49.8833335 -13.5070592 23.5960048

H 50.5777175 -13.1344392 22.0071598

H 51.9705575 -13.3014902 24.6436598

H 52.7088915 -13.1616282 23.0272468

C 53.8142545 -11.2213362 24.5087948

C 54.1564195 -12.1356352 25.6626508

H 54.4297655 -11.4656652 23.6270358

H 54.2149135 -13.2112152 25.4503958

C 54.5090865 -11.6852872 26.8823048

H 54.8303445 -12.3787952 27.6671288

H 54.5142485 -10.6179922 27.1373968

H 50.7913704 -8.3921909 22.7831879

H 53.9829355 -10.1660642 24.7711878

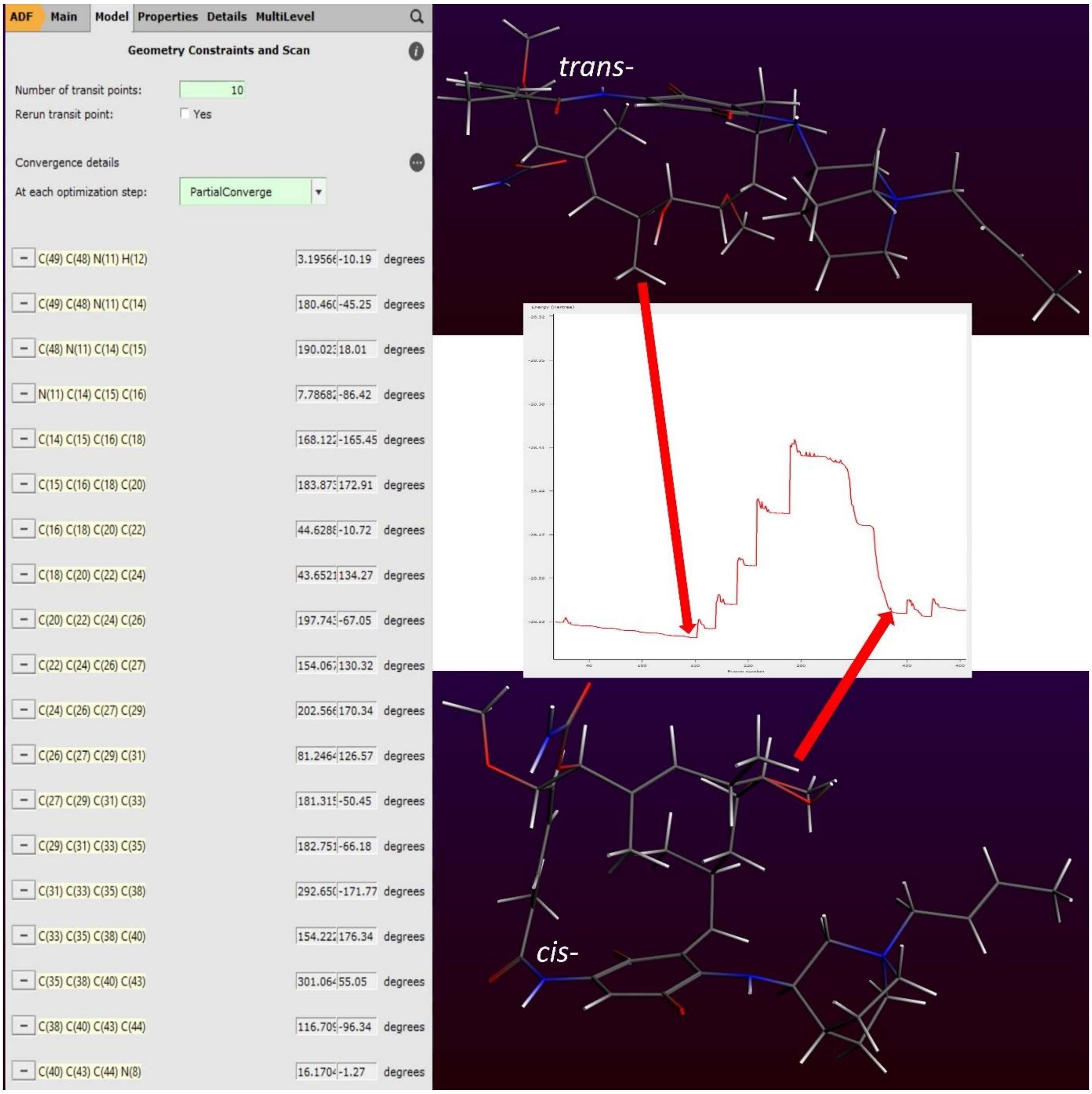

Figure 53S. Energy barrier calculations for atropisomerization of **10**, performed by BLYP GGA (DZP) DFT theoretical method (ADF package) [3,4]

X-ray crystallography

All crystals of the studied compounds were solvated and very unstable in the air. Diffraction data were collected at 130 K for crystals mounted on a plastic loop with small amount of perfluoropolyether. The diffraction measurements were carried out with a SuperNova diffractometer using hi-flux micro-focus Nova Cu-Kα radiation (λ=1.54184 Å). Data collection and reduction were performed with the CrysAlis Pro software.[6] An interesting phenomenon

was observed for crystals of **3**. These crystals consisted of two different monoclinic crystalline solvated phases, **3-1** (space group $P2_1$) and **3-2** (space group $I2$), that had a common unit cell vector **b**. The solvent content in both phases was different as indicated by the unit-cell volume [3838.8(2) $Å^3$ in **3-1** and 4382.9(7) $Å^3$ in **3-2** with four molecules of **3** per unit cell]. Thus the crystal structures of **3-1** and **3-2** were determined from the diffraction data obtained for the same crystal, with **3-2** constituting a minor crystal component and giving a rather poor diffraction (data resolution 0.9 Å). All structures were solved by directed methods using the SHELXT program.[7] The structures were refined by full-matrix least-squares method on F^2 with SHELXL-2018.[8] All calculations were carried out within OLEX-2.[9]

In **1**, **2** and **3-1** some solvent acetonitrile or acetone molecules with partial occupancy were located, however all four structures contained solvent accessible area (11%, 8%, 4% and 25% of the unit-cell volume in **1**, **2**, **3-2** and **3-2**, respectively) where solvent molecules could not be reliably identified. A disordered solvent contribution was extracted from structure factors using the 'Solvent mask' routine of Olex-2 with the probe radius of 1.2 Å and a resolution of 0.2 Å.

In case of **2**, **3-1** and **3-2** some disorder within a C(17) substituent of the geldanamycin skeleton was also observed. The hydrogen atom positions were determined geometrically and were refined in the riding-model approximation, with $U_{iso}(H) = 1.2\ U_{eq}(N,C)$ and $U_{iso}(H) = 1.5\ U_{eq}(O,C_{methyl})$.

The crystal data and some details of data collection and structure refinement are given in **Table 1S.** The molecular structures are illustrated in **Fig. 53S-56S** and crystal packing in **Fig. 57S-59S.**

CCDC 2035723-2035726 contain the supplementary crystallographic data for **1**, **2**, **3-1** and **3-2**.

Table 1S. Crystal data and details of structure refinement.

Compound reference	**1**	**2**	**3-1**	**3-2**
Chemical formula	$C_{32}H_{45}N_3O_8$· 0.721(C_2H_3N)· unknown solvent	$C_{31}H_{43}N_3O_{10}$· 0.686 (C_2H_3N)· unknown solvent	2($C_{34}H_{48}N_4O_9$)·C_3 H_6O· unknown solvent	$C_{34}H_{48}N_4O_9$· unknown solvent
Formula Mass	647.75	645.80	1371.60	656.76
Crystal system	Monoclinic	Triclinic	Monoclinic	Monoclinic
a/Å	15.42873(14)	7.9200(3)	16.7062(7)	18.7631(13)
b/ Å	7.88367(6)	15.1429(6)	7.9630(2)	7.9618(6)
c/ Å	16.13056(16)	15.9245(7)	29.7717(10)	29.620(4)
α/°	90	104.576(4)	90	90
β/°	106.726(1)	91.286(3)	104.243(4)	97.904(8)
γ/°	90	90.691(3)	90	90
Unit cell volume/Å^3	1879.03(3)	1847.61(13)	3838.8(2)	4382.9(7)
Temperature/K	130	130	131	131
Space group	$P2_1$	*P*1	$P2_1$	*I*2
No. of formula units per unit cell, Z	2	2	2	4
Radiation type	Cu $K\alpha$	Cu $K\alpha$	Cu $K\alpha$	Cu $K\alpha$
Absorption coefficient, μ/mm^{-1}	0.662	0.716	0.709	0.595
No. of reflections measured, Θ_{max}/°	29596, 66.6	18949, 66.6	29734, 66.6	15374, 58.93
No. of independent reflections	6627	8418	10720	5211
R_{int}	0.0376	0.0346	0.0432	0.0750
Final R_1 values (I> 2σ(I))	0.0342	0.0553	0.0767	0.1664
Final wR(F^2) values (I>2σ(I))	0.0916	0.0635	0.0897	0.1834
Final R_1 values (all data)	0.0356	0.1496	0.2086	0.3893
Final wR(F^2) values (all data)	0.0930	0.1566	0.2209	0.4107
Goodness of fit on F^2	1.059	1.056	1.045	1.516

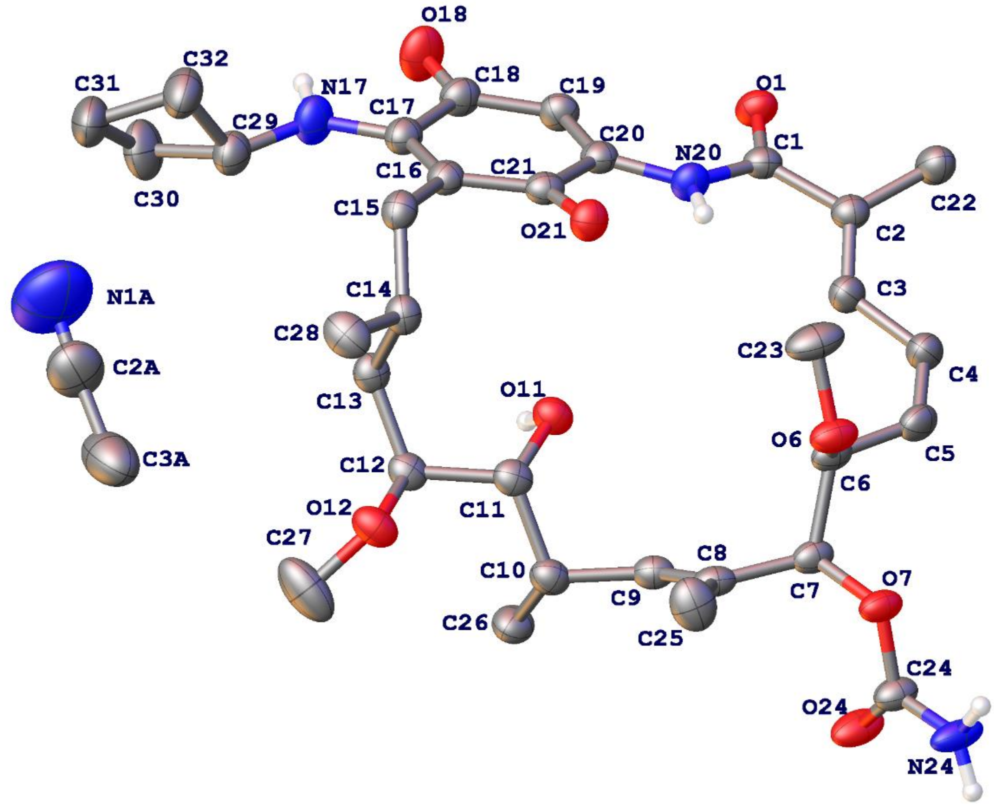

Figure 54S. Molecular structure of **1**. Displacement ellipsoids are shown at the 50% probability level. Hydrogen atoms from N-H and O-H groups are shown and those from C-H groups were omitted for clarity.

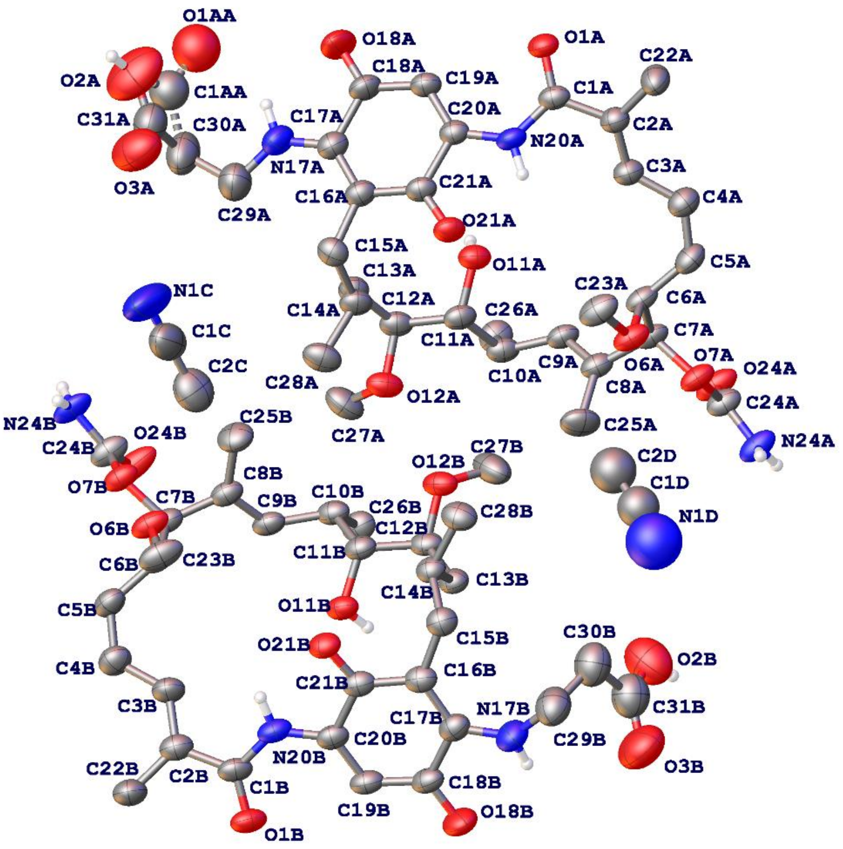

Figure 55S. Molecular structure of **2**. Displacement ellipsoids are shown at the 50% probability level. Hydrogen atoms from N-H and O-H groups are shown and those from C-H groups were omitted for clarity. The carboxylic group of C(17) substituent is disordered over two positions.

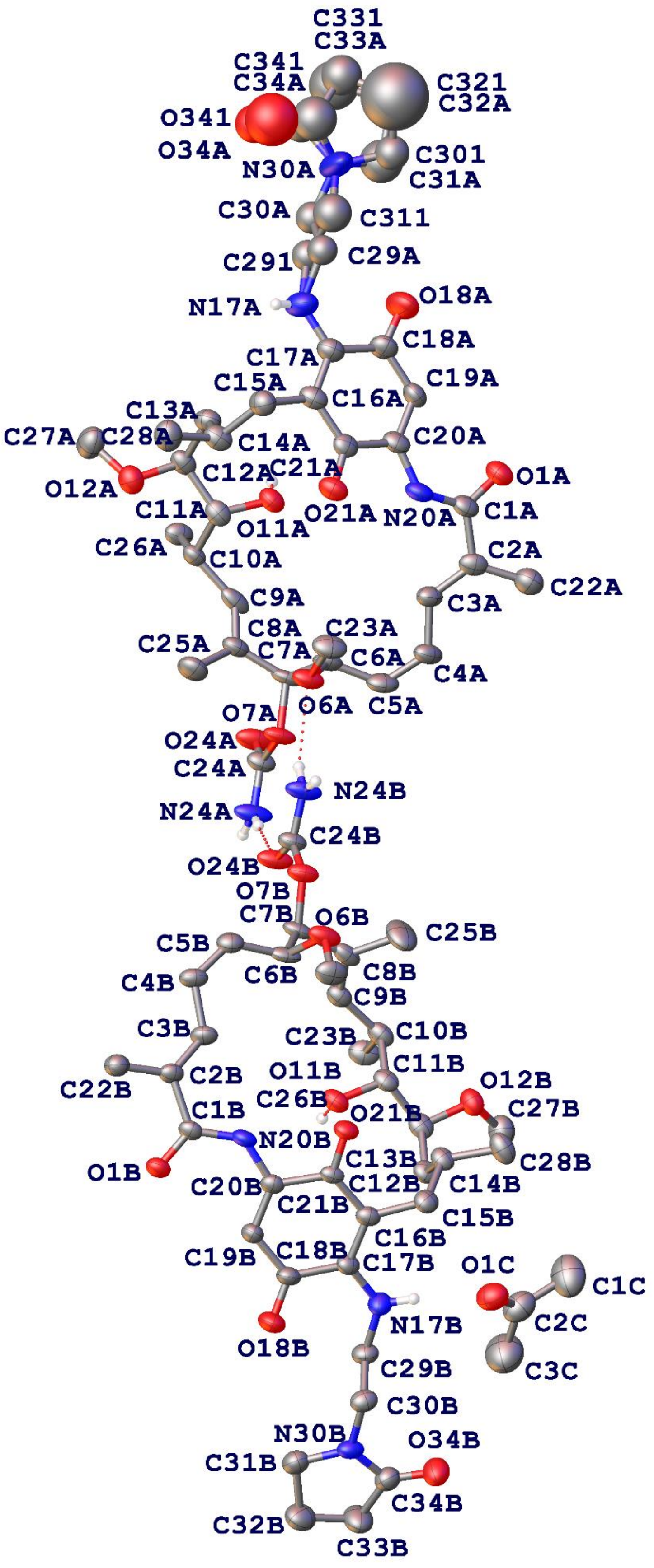
C331
C33A
C341
C34A
C321
C32A
O341
O34A
N30A
C301
C31A
C30A
C311
C291
C29A
N17A
O18A
C18A
C17A
C19A
C15A
C16A
C13A
C20A
C27A
C28A
C14A
C21A
C12A
O1A
O12A
C11A
O21A
N20A
C1A
O11A
C26A
C10A
C2A
C22A
C9A
C3A
C8A
C23A
C25A
C7A
C6A
C4A
O7A
O6A
C5A
O24A
C24A
N24B
N24A
C24B
O24B
O7B
C7B
O6B
C25B
C5B
C6B
C4B
C8B
C9B
C3B
C23B
C10B
C11B
O11B
O12B
C2B
C22B
C26B
C1B
O21B
C27B
N20B
C13B
C28B
O1B
C20B
C12B
C14B
C21B
C15B
C16B
C19B
C18B
C17B
O1C
C1C
C2C
N17B
O18B
C3C
C29B
C30B
N30B
O34B
C31B
C34B
C32B
C33B

Figure 56S. Molecular structure of **3 in 3_1**. Displacement ellipsoids are shown at the 50% probability level. Hydrogen atoms from N-H and O-H groups are shown and those from C-H groups were omitted for clarity. The C17 substituent of the A molecule is disordered over two positions.

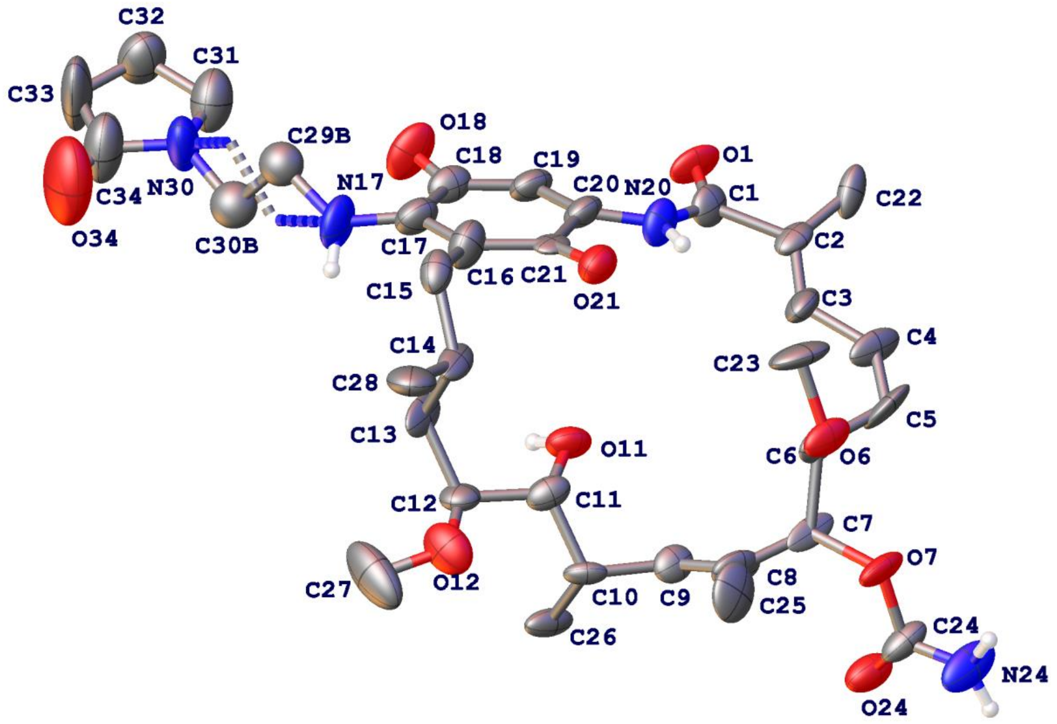

Figure 57S. Molecular structure of **3 in 3_2**. Displacement ellipsoids are shown at the 50% probability level. The hydrogen atoms from N-H and O-H groups are shown and those from C-H groups were omitted for clarity. The ethylene fragment of the C(17) substituent is disordered over two positions.

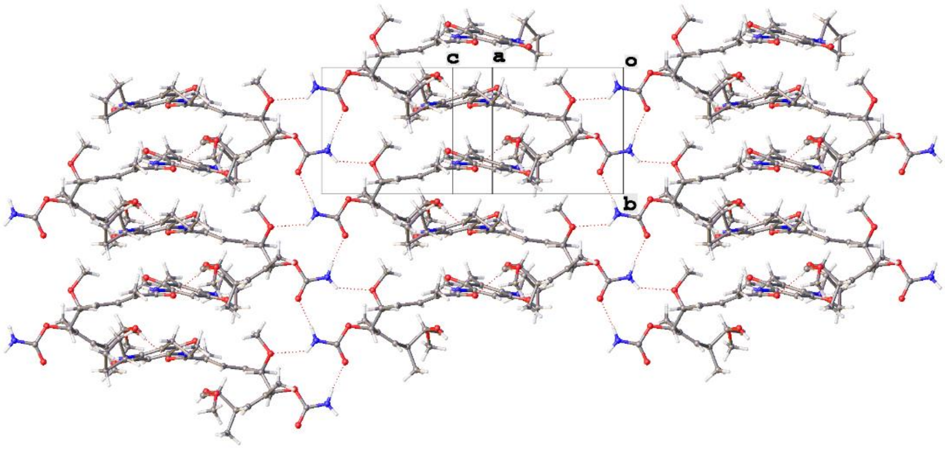

(a)

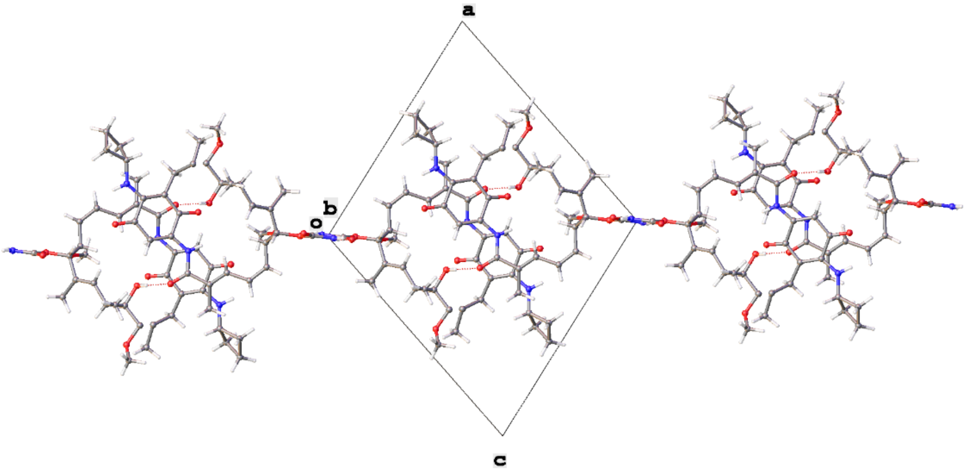

(b)

Figure 58S. Side (a) and top (b) views of a two dimensional supramolecular assembly formed reproducibly via hydrogen bonds in C(17) substituted geldanamycin derivatives.

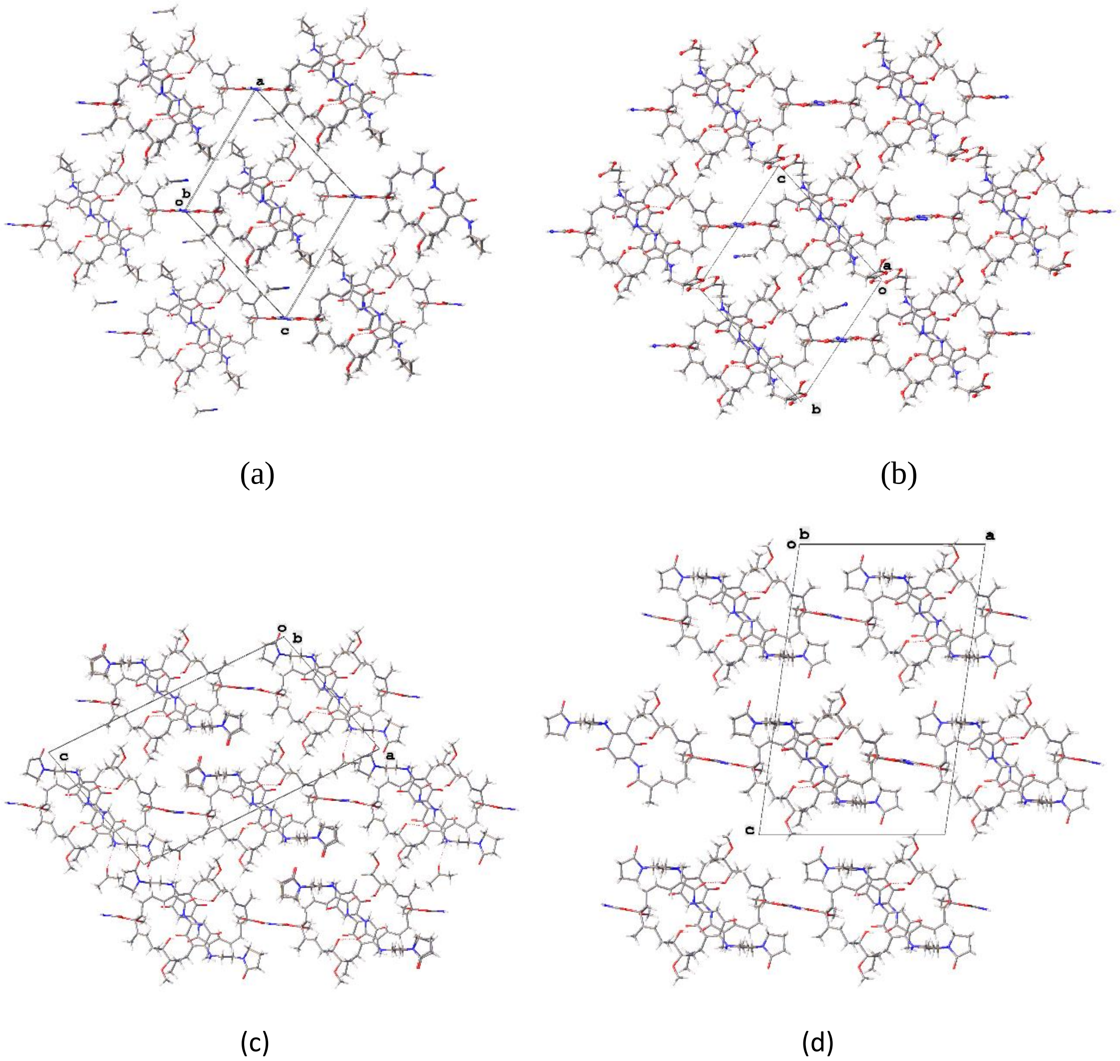

Figure 59S. Comparision of crystal packing of 2D supramolecular hydrogen-bonded assemblies in **1** (a), **2** (b), **3-1** (c) and **3-2** (d) viewed along the corresponding direction in the crystal lattice

Structure of GDM analogs in solution vs. solid

^{1}H-^{1}H NOESY contacts recorded for **1-13** showed the following significant spin-spin couplings: N(1)H···H(3), N(1)H···H(6), N(1)H···H(7), N(1)H···H(9) and N(1)H···O(11)H. These couplings indicate, that the proton of the lactam group, being in *trans*-configuration, is directed into the macrocyclic cavity, as shown in Fig. 60S In the case of the lactam *cis*–configuration the above contacts should not be observed in solution. The mutual orientation of the introduced

quinuclidine part relative to the benzoquinone ring was deduced by analyzing the contacts of the protons H(19) and H(15). In the ^{1}H-^{1}H NOESY spectra of **8** and its *N*-alkyl ammonium salts (**9-13**) we found the spin-spin couplings: H(19) ···H(13), H(19) ···H(15), H(19) ···H(22), H(19)···H(33), H(19) ···H(34), and H(15) ···H(29), H(15) ···H(32),

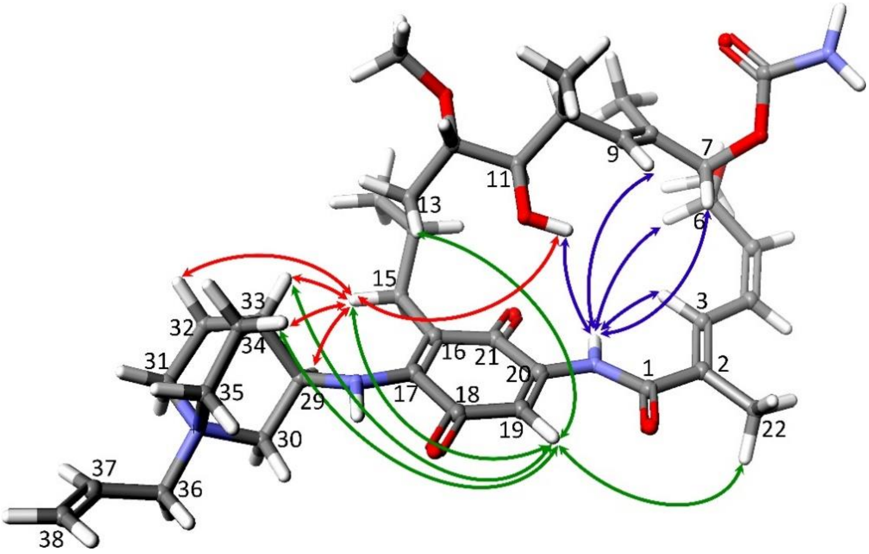

Figure 60S. General conformation of **GDM** quaternary ammonium salts in solution, determined on the basis of experimentally recorded key ^{1}H-^{1}H NOESY contacts, and calculated on that basis *via* B88 LYP (GGA) DFT method (*Scigress* package [1]); N(1)H contacts (violet), H(15) contacts (red), H(19) contacts (green).

H(15) ···H(33) and H(15) ···H(34) revealing an arrangement of the quinuclidine fragment relative to the amine-quinone moiety as shown in Fig. 60S The presence of molecular conformation showed in Fig. 60S in solution seems to be in line with the absence of any proton-proton contacts between *N*-alkyl substituents, the quinone and *ansa*-bridge parts, for all studied **GDM** analogs.

In the solid state, the structures of **GDM** derivatives **1-3** were determined by x-ray structural analysis. All these compounds crystallize as unstable solvates. Compound **3** is most interesting since two different solvated phases of this derivative were present within one crystal. In all studied crystal structures conformation of the molecular fragment consisting of the

benzoquinone core and the *ansa*-bridge is virtually identical and corresponds to the A conformer of **GDM** (Fig. 60S).[5]

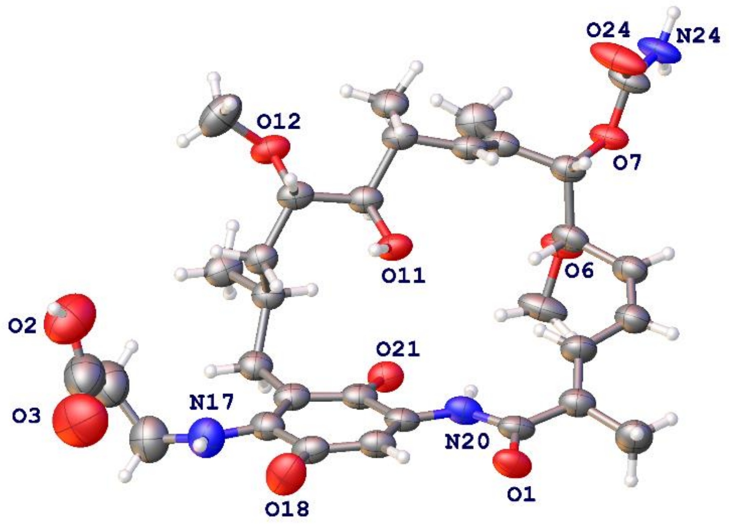

Figure 61S. Illustration of the A conformer of C(17) **GDM** analog on an example of one of the symmetry independent molecules in the crystal structure of **2**. The amino N(17)-H group is oriented towards the C(18)=O(18) carbonyl group.

We show that **GDM** and many of its C(17) derivatives tend to form isostructural 2D assemblies in crystals stabilized by a network of hydrogen bonds between the carbamate groups and between the *ansa*-chain OH and amide the carbonyl groups (see Fig. 57S)[5,10–12]. Molecules connected *via* hydrogen bonds form corrugated layers whereas their C(17) substituents are oriented towards the interlayer area. When the C(17) substituent contains groups able to participate in hydrogen bonding as proton donors and acceptors, for example in **2**, additional hydrogen bonds can be formed connecting the molecules into 3D assemblies. In the structure of **2** where the C(17) substituent bears a carboxylic group a typical dimeric motif is generated by the carboxylic groups belonging to adjacent layers (Fig. 58bS). The main structural difference between molecules **1-3** relates to the orientation of a substituent at the N(17) amino group relative to the benzoquinone C(18)=O(18) carbonyl group. In **1** and **2** the H atom of the

N(17)-H group is oriented towards O(18) whereas in both crystalline forms of **3** it points towards C(15). In the latter conformation the N(27)-H group is less shielded and thus able to bind solvent molecules *via* hydrogen bonds or to bind with the proton acceptor on a C(17) substituent[5].

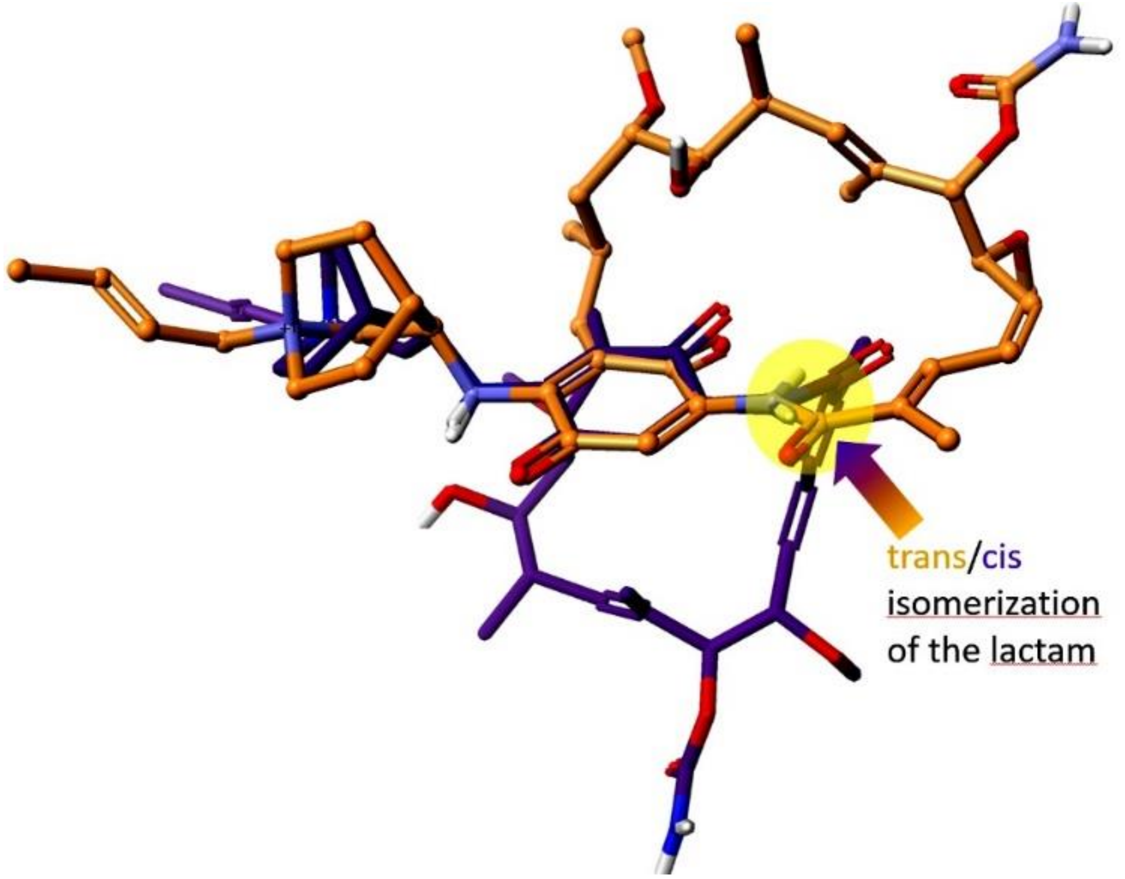

Figure 62S. Comparison of salt **10** conformations: bound with Hsp90 (*cis*-lactam, violet) and "free" in solution (*trans*-lactam, orange) calculated by B3LYP GGA (DZP) DFT (ADF package)[3,4], and visualized by Scigress (*Scigress* F.J. 2.6, EU 3.1.9)[1].

References:

[1] Scigress package FJ 2.6 /EU 3.1.9./ 2008-2019, Fujitsu, Japan, n.d.
[2] R.P.D. Bank, RCSB PDB - 3Q5J: Crystal structure of the amino-terminal domain of HSP90 from Leishmania major, LMJF33.0312:M1-K213 in the presence of 17-DMAP-geldanamycin, (n.d.). https://www.rcsb.org/structure/3Q5J (accessed May 7, 2020).
[3] ADF 2019.3, SCM, Theoretical Chemistry, Vrije Universiteit, Amsterdam, The Netherlands, (n.d.).

[4] G. te Velde, F.M. Bickelhaupt, E.J. Baerends, C.F. Guerra, S.J.A. van Gisbergen, J.G. Snijders, T. Ziegler, Chemistry with ADF, Journal of Computational Chemistry. 22 (2001) 931–967. https://doi.org/10.1002/jcc.1056.

[5] N. Skrzypczak, K. Pyta, P. Ruszkowski, M. Gdaniec, F. Bartl, P. Przybylski, Synthesis, structure and anticancer activity of new geldanamycin amine analogs containing C(17)- or C(20)- flexible and rigid arms as well as closed or open ansa-bridges, European Journal of Medicinal Chemistry. 202 (2020) 112624. https://doi.org/10.1016/j.ejmech.2020.112624.

[6] Rigaku Oxford Diffraction, Rigaku Corporation, Oxford, UK, 2018.

[7] G.M. Sheldrick, Crystal structure refinement with *SHELXL*, Acta Crystallogr C Struct Chem. 71 (2015) 3–8. https://doi.org/10.1107/S2053229614024218.

[8] G.M. Sheldrick, A short history of *SHELX*, Acta Crystallogr A Found Crystallogr. 64 (2008) 112–122. https://doi.org/10.1107/S0108767307043930.

[9] O.V. Dolomanov, L.J. Bourhis, R.J. Gildea, J.A.K. Howard, H. Puschmann, *OLEX2* : a complete structure solution, refinement and analysis program, J Appl Crystallogr. 42 (2009) 339–341. https://doi.org/10.1107/S0021889808042726.

[10] A. Baksh, B. Kepplinger, H.A. Isah, M.R. Probert, W. Clegg, C. Wills, M. Goodfellow, J. Errington, N. Allenby, M.J. Hall, Production of 17-O-demethyl-geldanamycin, a cytotoxic ansamycin polyketide, by Streptomyces hygroscopicus DEM20745, Natural Product Research. 31 (2017) 1895–1900. https://doi.org/10.1080/14786419.2016.1263854.

[11] R.C. Schnur, M.L. Corman, Tandem [3,3]-Sigmatropic Rearrangements in an Ansamycin: Stereospecific Conversion of an (S)-Allylic Alcohol to an (S)-Allylic Amine Derivative, J. Org. Chem. 59 (1994) 2581–2584. https://doi.org/10.1021/jo00088a047.

[12] W. Sun, Y.-P. Li, J. Jin, Z.-R. Li, G.-Z. Shan, Crystal structure of 17-(2’-(1’-oxa-4’-aza-heterocyclohexyl-1’-)ethylamino)- 17-demethoxygeldanamycin dihydrate, C34H54N4O11, Zeitschrift Für Kristallographie - New Crystal Structures. 230 (2015) 180–182. https://doi.org/10.1515/ncrs-2014-9073.

[13] S. Raman, M. Singh, U. Tatu, K. Suguna, First Structural View of a Peptide Interacting with the Nucleotide Binding Domain of Heat Shock Protein 90, Scientific Reports. 5 (2015) 1–10. https://doi.org/10.1038/srep17015.

[14] C.E. Stebbins, A.A. Russo, C. Schneider, N. Rosen, F.U. Hartl, N.P. Pavletich, Crystal Structure of an Hsp90–Geldanamycin Complex: Targeting of a Protein Chaperone by an Antitumor Agent, Cell. 89 (1997) 239–250. https://doi.org/10.1016/S0092-8674(00)80203-2.

[15] J.M. Jez, J.C.-H. Chen, G. Rastelli, R.M. Stroud, D.V. Santi, Crystal Structure and Molecular Modeling of 17-DMAG in Complex with Human Hsp90, Chemistry & Biology. 10 (2003) 361–368. https://doi.org/10.1016/S1074-5521(03)00075-9.

[16] M.-Q. Zhang, S. Gaisser, M. Nur-E-Alam, L.S. Sheehan, W.A. Vousden, N. Gaitatzis, G. Peck, N.J. Coates, S.J. Moss, M. Radzom, T.A. Foster, R.M. Sheridan, M.A. Gregory, S.M. Roe, C. Prodromou, L. Pearl, S.M. Boyd, B. Wilkinson, C.J. Martin, Optimizing Natural Products by Biosynthetic Engineering: Discovery of Nonquinone Hsp90 Inhibitors [†], J. Med. Chem. 51 (2008) 5494–5497. https://doi.org/10.1021/jm8006068.

[17] P. Thepchatri, T. Eliseo, D.O. Cicero, D. Myles, J.P. Snyder, Relationship Among Ligand Conformations in Solution, in the Solid State, and at the Hsp90 Binding Site: Geldanamycin and Radicicol, J. Am. Chem. Soc. 129 (2007) 3127–3134. https://doi.org/10.1021/ja064863p.

[18] Y.-S. Lee, M.G. Marcu, L. Neckers, Quantum Chemical Calculations and Mutational Analysis Suggest Heat Shock Protein 90 Catalyzes Trans-Cis Isomerization of Geldanamycin, Chemistry & Biology. 11 (2004) 991–998. https://doi.org/10.1016/j.chembiol.2004.05.010.
